# Lysis to Kill: Evaluation of the Lytic Abilities, and Genomics of Nine Bacteriophages Infective for *Gordonia* spp. and Their Potential Use in Activated Sludge Foam Biocontrol

**DOI:** 10.1371/journal.pone.0134512

**Published:** 2015-08-04

**Authors:** Zoe A. Dyson, Joseph Tucci, Robert J. Seviour, Steve Petrovski

**Affiliations:** 1 La Trobe Institute of Molecular Sciences, Bundoora, Victoria, Australia; 2 Department of Physiology, Anatomy and Microbiology, La Trobe University, Bundoora, Victoria, Australia; ContraFect Corporation, UNITED STATES

## Abstract

Nine bacteriophages (phages) infective for members of the genus *Gordonia* were isolated from wastewater and other natural water environments using standard enrichment techniques. The majority were broad host range phages targeting more than one *Gordonia* species. When their genomes were sequenced, they all emerged as double stranded DNA *Siphoviridae* phages, ranging from 17,562 to 103,424 bp in size, and containing between 27 and 127 genes, many of which were detailed for the first time. Many of these phage genomes diverged from the expected modular genome architecture of other characterized *Siphoviridae* phages and contained unusual lysis gene arrangements. Whole genome sequencing also revealed that infection with lytic phages does not appear to prevent spontaneous prophage induction in *Gordonia malaquae* lysogen strain BEN700. TEM sample preparation techniques were developed to view both attachment and replication stages of phage infection.

## Introduction

Many isolates of members of the actinobacterial genus *Gordonia* have been cultured from wastewater treatment plants [1] where they probably play a key role in degrading the more recalcitrant influent substrates [2, 3]. They include *Gordonia amarae*, an organism with a characteristic right-angled branching morphology, and among the first foam forming bacteria isolated and cultured [1, 4, 5]. Other *Gordonia* species and members of closely related genera share this distinctive morphology, and so in the absence of more precise identification, those with it are commonly referred to as *Gordonia amarae*-like organisms, or GALO [1].

Members of the *Corynebacteriales*, which include *Gordonia*, *Nocardia*, *Rhodococcus*, *Tsukamurella* and *Mycobacterium*, are often referred to collectively as the Mycolata because they alone synthesize long chain hydroxylated mycolic acids, organized as an exocellular outer membrane [6]. Their presence renders these cells highly hydrophobic. In activated sludge, high levels of these Mycolata stabilize foams formed on the surface of aeration tanks and clarifiers [1]. Formation of these stable foams is a global problem that impacts negatively on plant aesthetics, increases maintenance costs, and complicates sludge management [7]. Some of the Mycolata in these foams are opportunistic pathogens, thus posing a potential health hazard to plant operators from their aerosol dispersal [1, 7, 8].

Formation of these stable foams requires air bubbles, surface active agents, and hydrophobic particles, in this case the Mycolata cells [9]. A successful control strategy must be directed at the hydrophobic bacteria because neither air bubbles nor detergents can be eliminated from the activated sludge process [9]. Current foam control strategies are not effective universally, and no single method reliably controls all foams. This probably is a reflection of how little is known about the microbial ecology of these causative bacteria [1]. It was Thomas et al., [10] who first proposed that phage therapy could be exploited using the natural lytic cycles of phages as an attractive and environmentally friendly approach to selectively control their population levels without affecting other desirable bacteria in these systems.

Currently (February 2015) 228 phages targeting members of the genus *Mycobacterium* have had their genomes sequenced, and only four lytic *Gordonia* phage genome sequences are available. These are phages GTE2 [11], GTE7 [12], GRU1 and GTE5 [13]. All *Gordonia* phages isolated so far have distinctive genome sequences [11–13]. Yet with such a small sample size, it is not sensible to comment on the general characteristics of *Gordonia* phages and draw conclusions from these as to their suitability or otherwise for foam bio-control. Therefore, more *Gordonia* lytic phages are needed, including those from habitats other than activated sludge plants.

This study set out to increase the small existing library of *Gordonia* phages, and to characterize them in terms of their host ranges, morphologies, and genomics. Nine phages infective for members of this genus were isolated and their suitability for use in *Gordonia* foam biological control was investigated.

## Materials and Methods

No specific permission was required for sample collection from the water locations sampled as all samples were publically available for researchers to collect. All fieldwork conducted in this study did not involve endangered or protected species.

### Isolation and preliminary characterization of *Gordonia* phages

Host strains held in the La Trobe University culture collection used in this study, and the techniques for their growth are those detailed by Petrovski et al., [14], together with those listed in Table 1, which were grown in the same manner. All phages were isolated and subsequently purified from water samples collected from a variety of locations using enrichment pools of multiple host strains, as shown in Table 1 and described previously [14]. Phage host range specificity determinations were also carried out as described by [14].

**Table 1:** Isolation and characterization of nine *Gordonia* phage.

Phage	Sample	Strain	Lab ID	Enrichment pool members	Host range
**GMA2**	Activated sludge, Kyneton, Victoria, Australia	*G*. *malaqaue*	A448	See GTE8	*G*. *terrae* (CON34, GOR9, G238), *G*. *malaquae* (CON59, CON60, A554, A448), *G*. *hydrophobica* (CON65, CON66)
**GMA3**	Wastewater, Glenelg, South Australia, Australia	*G*. *malaquae*	BEN700	See GTE8	*G*. *terrae* (G238), *G*. *malaquae* (BEN700)
**GMA4**	Puddle water and sediment, Reservoir, Victoria, Australia	*G*. *malaquae*	BEN700	See GTE8	*G*. *malaquae* (BEN700)
**GMA5**	Activated sludge, Carrum (Eastern Treatment Plant), Victoria, Australia	*G*. *malaquae*	BEN700	See GTE8	*G*. *rubropertincta* (CON38), *G*. *terrae* (G238, G232), *G*. *malaquae* (BEN700)
**GMA6**	Activated sludge, Bendigo, Victoria, Australia	*G*. *malaquae*	CON67	See GTE8	*G*. *malaquae* (CON59, CON60, CON67, A554, A448, BEN700), *G*. *terrae* (G238)
**GMA7**	Activated sludge, Werribee, Victoria, Australia	*G*. *malaquae*	CON60	*G*. *terrae* (GOR9, G232, G238), *G*. *malaquae* (A554, A448, CON60, BEN700), *T*. *paurometabola* (CON61)	*G*. *terrae* (CON34, GOR9, G238), *G*. *rubropertincta* (CON38), *G*. *malaquae* (CON59, CON60, A554, A448, BEN700), *G*. *hydrophobica* (CON65, CON66)
**GRU3**	Wastewater, Inverell, Queensland, Australia	*G*. *rubropertincta*	CON38	See GTE8	*G*. *rubropertincta* (CON38), *G*. *terrae* (GOR9, G232)
**GTE6**	Activated sludge, Nambour, Queensland, Australia	*G*. *terrae*	CON34	*G*. *terrae* (CON34, BEN601, BEN604), *G*. *sputi* (CON48, CON49), *G*. *amarae* (CON44, CON9)	*G*. *terrae* (CON34, GOR9), *G*. *malaquae* (CON59, CON60, A554, A448), *G*. *hydrophobica* (CON65, CON66)
**GTE8**	Bendigo creek water, Bendigo, Victoria, Australia	*G*. *terrae*	G232	*G*. *terrae* (CON34, G238, G290, G255, G232, GOR9), *G*. *sputi* (CON48, CON49), *G*. *amarae* (CON44, CON9), *G*. *hydrophobica* (CON65, CON66), *G*. *desulfuricans* (CON69), *G*. *polyisoprenovorans* (CON71), *G*. *alkanivorans* (CON72), *G*. *malaquae* (A554, A448, BEN700, CON67), *T*. *inchonensis* (BEN701), *R*. *erythropolis* (BEN703) *G*. *aicheiensis* (CON22)	*N*. *asteroids* (CON12), *G*. *terrae* (CON34, GOR9, G232), *G*. *rubropertincta* (CON38)

### Transmission electron microscopy of virion morphology

Grids for visualization of virions were prepared with the negative stain uranyl acetate [14]. Both carbon and formvar coated grids were used (Electron Microscopy Sciences, Australia), with the exception of phage GTE6 which was examined on grids coated with formvar only. Prepared grids were subsequently examined with a JEOL JEM-100CX, JEOL JEM-2010HC, or a Tenaci Fei T30 Transmission Electron Microscope (Table 2).

**Table 2:** *Gordonia* phage virion measurements.

Phage name	Capsid diameter (nm)	Tail length (nm)
**GMA2** ^**b**^	61 ± 4	386 ± 3
**GMA4** ^**b**^	54 ± 2	244 ± 2
**GMA5** ^**b**^	37 ± 2	85 ± 9
**GMA6** ^**b**^	62 ± 2	143 ± 7
**GMA7** ^**c**^	63 ± 3	474 ± 9
**GRU3** ^**b**^	43 ± 2	93 ± 10
**GTE6** ^**a**^	48 ± 8	152 ± 12
**GTE8** ^**b**^	56 ± 2	239 ± 12

^a^ electron micrographs obtained using a JEOL JEM-100CX,

^b^ electron micrographs obtained using a Tenaci Fei T30,

^c^ electron micrographs obtained using a JEOL JEM-2010HC.

### Transmission Electron Microscopy to show phage infection

To visualize phage attachment, a single colony of *Gordonia malaquae* (CON67) was taken from a streak plate incubated at 30°C for 3 days. The cells were added to 20 μL of high titer GMA6 phage lysate (>10^10^ PFU/mL), and left to stand for 10 min to allow attachment before they were adsorbed onto the surface of carbon/formvar coated 200 mesh copper grids (Electron Microscopy Sciences, Australia). Grids were washed twice in sterile double-distilled water (ddH_2_O), and then negatively stained with 2% (w/v) uranyl acetate for 2 min. Excess liquid was absorbed onto filter paper and the grid was allowed to air dry. These grids were then examined under a JEOL JEM-2010HC Electron Microscope.

For phage assembly, a 1 mL aliquot of a *Gordonia terrae* (CON34) culture incubated at 30°C for three days in PYCA broth was removed carefully and added to 20 mL of PYCa broth together with 100 μL of high titer phage GTE6 suspension (>10^10^). This mixture was allowed to stand for 10 mins before further incubation at 30°C for three days. A 1.5 mL aliquot was then centrifuged (3,000 x g for 30 min) and the supernatant discarded. Pelleted cells were re-suspended and fixed in 2.5% (v/v) glutaraldehyde in 0.1 M phosphate buffer (pH 6.8–7.3), and incubated at 4°C overnight, then harvested (14,000 x g for 5 min) and washed in the same phosphate buffer 3 times, with 10 min between washes. Cells were post-fixed in 1% osmium tetroxide in 0.1 M phosphate buffer for 90 min and washed three times in sterile ddH_2_O. They were then dehydrated through an acetone series of increasing concentrations (30%, 50%, 70%, 90% and 100%) for 10 min each, prior to a final washing with 100% acetone with a molecular sieve (ProSciTech, Australia) (10 min). Dried specimens were infiltrated with Spurr’s epoxy resin (ProSciTech, Australia), initially with 50% resin, 50% dehydrated acetone, and incubated overnight at room temperature. The mixture was replaced by 100% Spurr’s resin with a further incubation of 1–2 h, before finally being replaced by fresh Spurr’s expoxy resin, and polymerised at 65°C overnight. Thin sections (100 nm) were cut with a glass knife on an LKB Microtome and post-stained with uranyl acetate and lead citrate. Sections were placed on 200 mesh copper grids and examined as described above.

### Mass spectroscopy

To identify phage structural proteins, purified virions >10^13^ PFU/mL were precipitated with (NH_4_)_2_SO_4_ followed with exposure to ZnCl_2_ to remove any residual polyethylene glycol from the previous step. Pellets were re-suspended in 8 M urea to a final volume of 100 μL prior to transfer to the Mass Spectroscopy and Proteomics facility at the La Trobe University Institute of Molecular Sciences. Here peptides reconstituted in 0.1% formic acid and 2% acetonitrile (buffer A) were loaded onto a trap column (C18 PepMap 300 μm i.d. × 2 cm trapping column, Thermo-Fisher Scientific) at 5 μL/min for 6 min and washed for 6 min before switching the precolumn in line with the analytical column (Vydac MS C18, 75 μm i.d. × 25 cm, Grace Davison). The separation of peptides was performed at 300 nL/min using a linear acetonitrile (ACN) gradient of buffer A and buffer B (0.1% formic acid, 80% ACN), starting from 5% buffer B to 40% over 60 min. Data were collected on an hybrid quadrupole/time-of-flight MS (MicroTOF-Q, Bruker, Germany) with a nano-electrospray ion source using Data Dependent Acquisition mode and *m*/*z 15*0–2500 as MS scan range. Nitrogen was used as the collision gas. The ionisation tip voltage and interface temperature were set at 4200 V and 205°C respectively. Collision Induced Dissociation (CID) MS/MS spectra were collected for the 3 most intense ions. Dynamic exclusion parameters were set as follows: repeat count 2, duration 60 s. The data were collected and analysed using Data Analysis Software (Bruker Daltonics, Bremen, Germany).

### Genome sequencing of *Gordonia* phages

Genomic DNA was extracted from phages GTE6, GMA2, and GMA6 and sequenced using a Roche GS FLX genome sequencer and titanium chemistry, as described in Petrovski, Seviour [14]. Genomic DNA extracted from all other phages in the same manner was prepared with an Illumina Nextera XT sample preparation kit as per manufacturers’ instructions. The prepared DNA libraries were sequenced on an Illumina MiSeq as a 150 bp paired end run.

### Genome annotation

The genome open reading frames (ORFs) were screened initially using Glimmer (v3.02), where ORFs with a minimum size of 90 bp were detected [15]. All predicted start codons were inspected for the presence of putative ribosomal binding sites and corrected as necessary. Sequence similarity searches were carried out against the GenBank database, as described by Petrovski et al. [11]. The presence of tRNA and tmRNA were also determined using both ARAGORN [16], and tRNAScan-SE [17]. Transmembrane domains were predicted with the DAS Transmembrane Prediction server [18].

Phage DNA when analyzed by gel electrophoresis gave results consistent with circularly permuted DNA genomes. Therefore, for consistency the genomes annotations were conducted starting with the DNA packaging operon.

### Nucleotide sequence accession number

The nucleotide sequences for all phages have been deposited GenBank under the following accession numbers; GTE6 (KR053200), GTE8 (KR053201), GMA2 (KR063281), GMA3 (KR063279), GMA4 (KR053199), GMA5 (KR053198), GMA6 (KR063280), GMA7 (KR063278), and GRU3 (KR053197).

## Results and Discussion

### Phage isolation and host range characterization

All *Gordonia* phages isolated previously were obtained from wastewater, with most coming from activated sludge plants on the east coast of Australia [10–13]. While most phage isolates described here were also from wastewater (Table 1), an additional two phages GMA4 and GTE8, were obtained from puddle sediment (Reservoir, Australia), and creek water (Bendigo, Australia), respectively.

One of these, phage GMA4, lysed a single Mycolata strain, *Gordonia malaquae* strain BEN700. Phage GMA3 lysed the same *G*. *malaquae* strain (BEN700), but, also *G*. *terrae* (G238). All the other phage’s lysed multiple *Gordonia* strains, with phage GMA7 attacking 11 strains from four different *Gordonia* species i.e. *G*. *terrae* (CON34, GOR9, G238), *G*. *rubropertincta* (CON38), *G*. *malaquae* (CON59, CON60, A554, A448, BEN700), and *G*. *hydrophobica* (CON65, CON66). As well as phage GTE8 lysing three strains of *Gordonia terrae* (CON34, GOR9, G232) and one of *G*. *rubropertincta* (CON38), it could also lyse *Nocardia asteroides* (CON12).

Phages able to lyse members of both these genera have been reported before. They include phage GRU1, which targets *Nocardia nova* strain CON47 and *Gordonia terrae* strains CON34, and G232 and also *Gordonia rubropertincta* strain CON38 [13]. This outcome might reflect the close phylogenetic relationship of these host bacteria.

Many of these overlap in their host ranges. For example phages GTE6, GMA2, GMA6, and GMA7 all lysed the same four strains of *G*. *malaquae* (CON59, CON60, A554, and A448), a property which might make them useful additions to any phage cocktail designed to target foaming caused by *G*. *malaquae*, especially if they use different host receptor sites. Phage GMA5 was lytic against two of eight *G*. *terrae* strains (G238, G232). A similar situation has been reported for other *Gordonia* phages, including GTE2 [11] that lysed only one of five *G*. *terrae* strains. No phages infective for *G*. *amarae* were obtained in this study.

### Virion morphology

**Fig 1:**
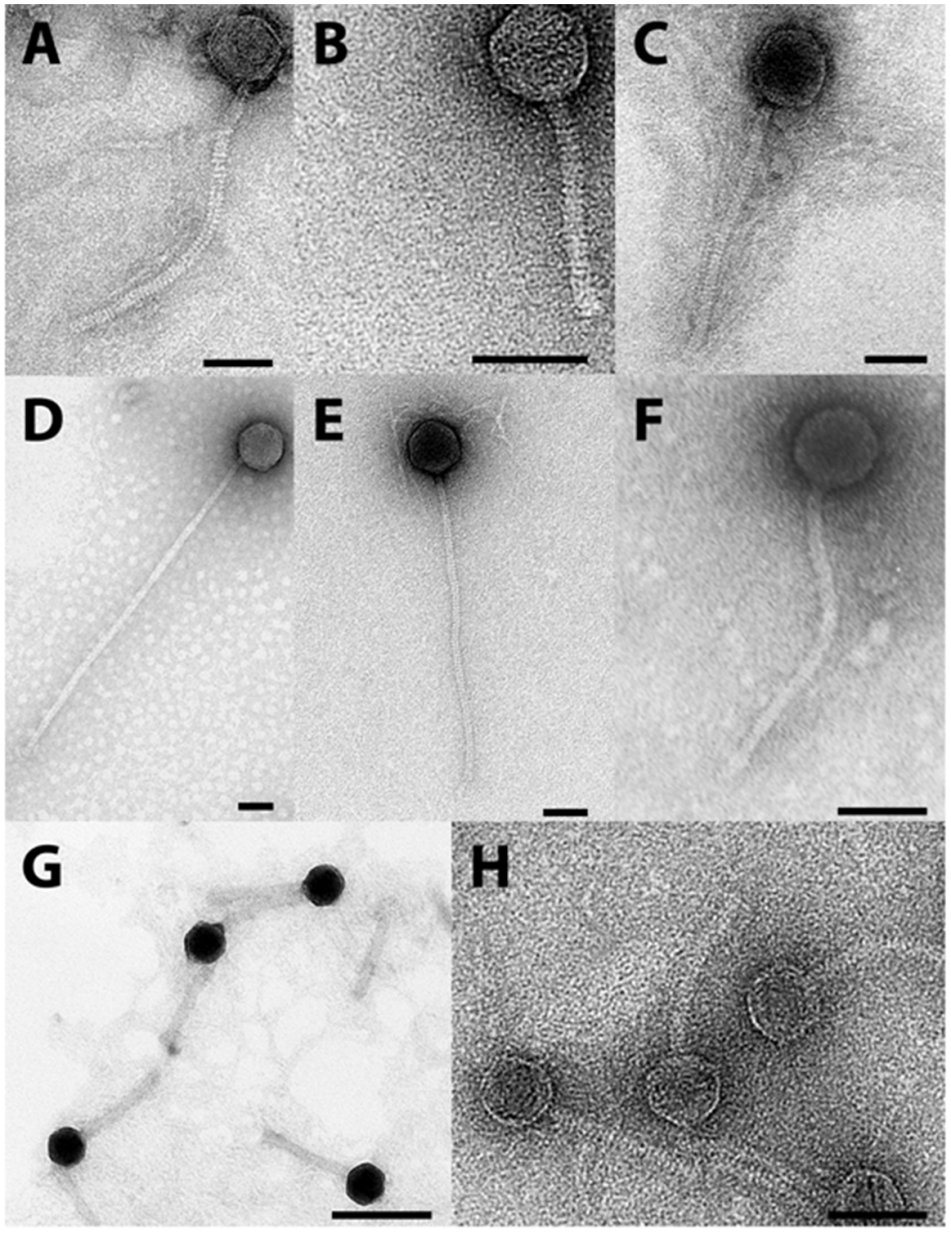
TEM *Gordonia* phage morphologies. (A) Phage GMA4 (B) Phage GRU3 (C) Phage GTE8 (D) Phage GMA7 (E) Phage GMA2 (F) Phage GTE6 (G) Phage GMA6 (H) Phage GMA5. Scale = 50 nm.

**Fig 2:**
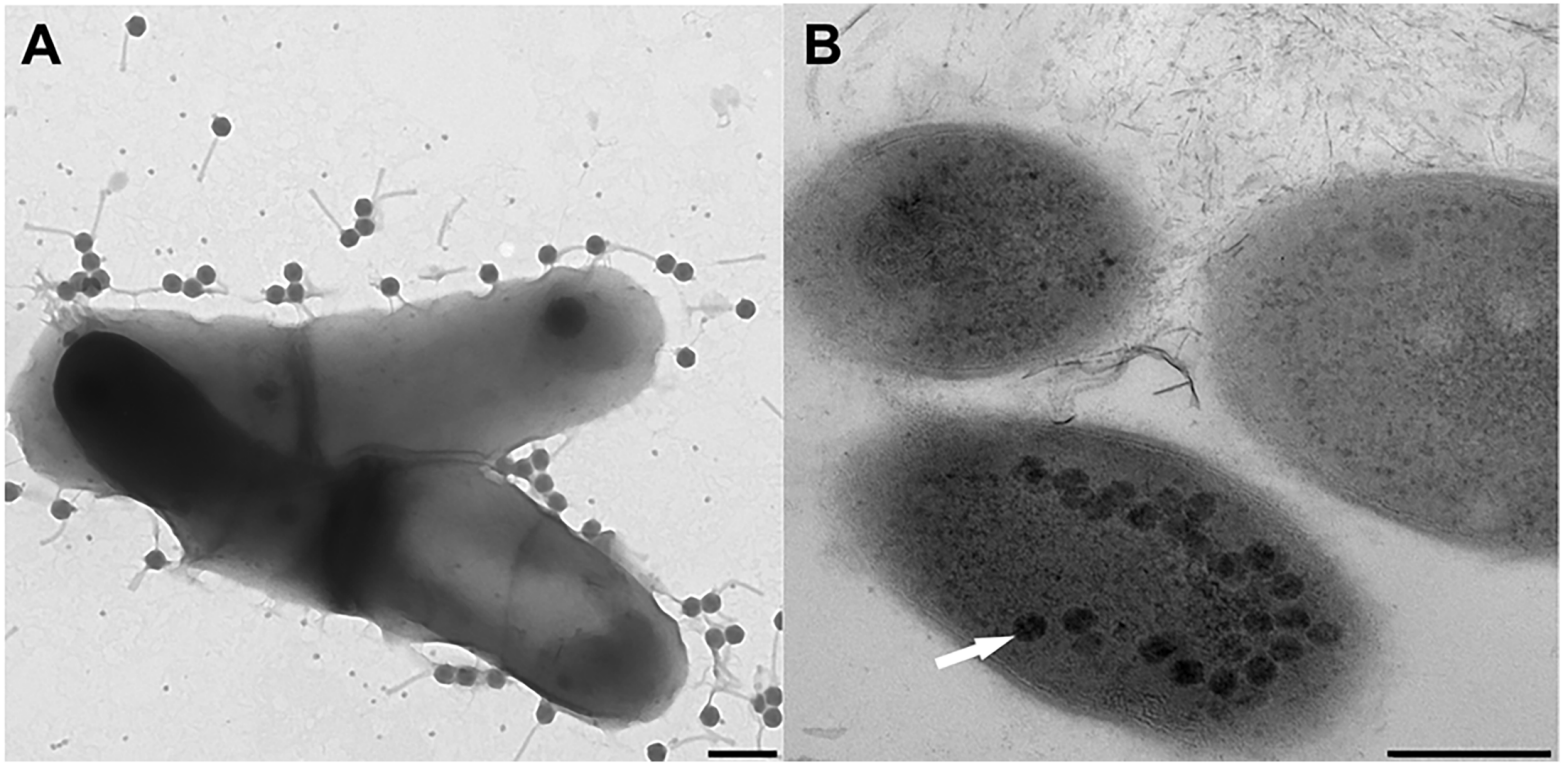
Stages in *Gordonia* phage infection cycles. (A) Attachment stage of phage infection cycle between phage GMA6 and host *Gordonia malaquae* strain CON67. Scale = 200 nm. (B) Replication of phage GTE6 inside *G*. *terrae* strain CON34 cells prior to cell lysis. Arrows indicate phage replicated inside bacterial cells. Scale = 200 nm.

All phages examined by TEM had both the isometric type B1 capsids (~ 37 to ~ 63 nm in diameter) and long, non-contractile tails (~ 85 to ~ 474 nm long) characteristic of members of the family *Siphoviridae*. Phage GMA3 was not examined by TEM, but based on its genome sequence which contained a gene encoding a long tape measure protein and its dsDNA genome, it too is most likely to be a member of the *Siphoviridae* [19]. Further details are provided in Fig 1, and Table 2. With TEM, the morphology of phage GMA6 was not that expected of a *Siphoviridae* member, since its tail appeared to be uncharacteristically thick and rigid (Fig 1). To resolve this concern, phage GMA6 virions were exposed briefly to *G*. *malaquae* strain CON67, it’s isolating host, and then examined by TEM. Images showed clearly (Fig 2a) that its phage tail can be flexible, confirming it as a *Siphoviridae* member. Furthermore, TEM (Fig 2a) shows that virion attachment can involve many phages simultaneously. Whether superinfections where more than one phage genome successfully invades the host cell, was not explored. We could also visualize post replication mature phage progeny within the host cell, thus Fig 2b shows mature GTE6 virions inside the host cells, prior to cell lysis and release of phage progeny.

### Structure and organization of *Gordonia* phages genomes

**Fig 3:**
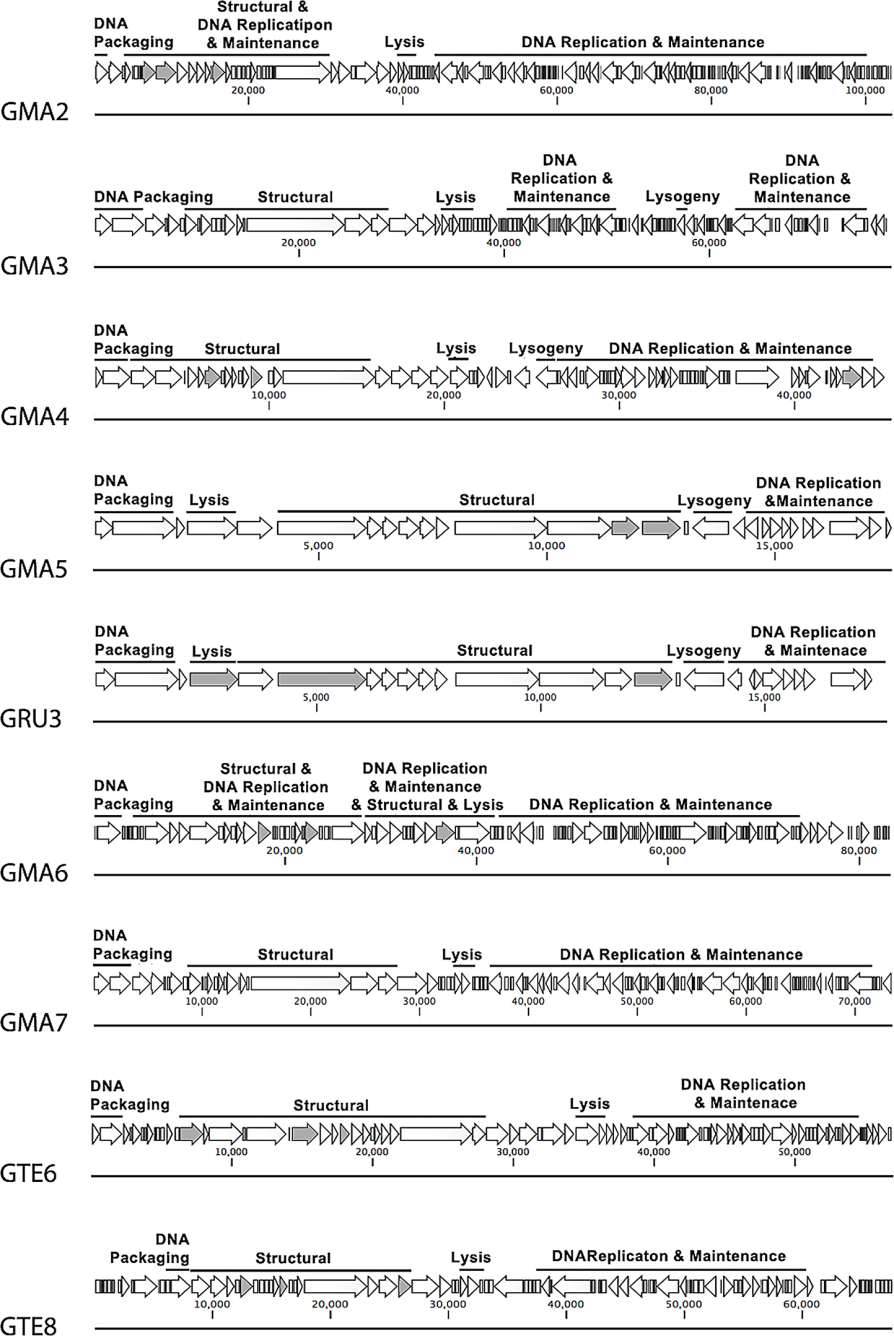
Genome map of nine *Gordonia* phages. Grey indicates structural genes identified with mass spectroscopy data.

When the assembled genomes for all nine *Gordonia* phages were examined, they were in most cases distinctively different to each other. Genome sizes ranged from 17, 562 to 103, 424 bp, and they contained between 27 to 127 putative *orf*s, arranged mostly in the modular architecture commonly seen in the *Siphoviridae* phages (Table 3, Fig 3). All contained putative genes orientated in both forward and reverse directions, with the one exception being phage GTE6, where all its genes were in a forward orientation. Only between 22 and 50% of the putative genes identified in the nine *Gordonia* phage genomes could be annotated functionally (Table 3, S1 Table). The G+C mol % contents of all phages ranged from 51.3 to 67.8 mol % (Table 3) and for the majority this value was close to that of the corresponding host cells [6].

**Table 3:** Summary of characters of the nine *Gordonia* phage genomes.

Phage name	Average coverage (fold)	Total read count	Genome size (bp)	G+C content (mol %)	No. putative tRNA	No. putative genes	No. putative genes in forwards orientation	No. functionally annotated putative genes	No. novel genes	No. palindromes	No. direct repeats	No. inverted repeats
**GMA2** ^**a**^ ^**c**^	1, 212	336, 750	103, 424	53.4	16	126	42	42	62	7	22	10
**GMA3** ^**b**^ ^**e**^ ^**d**^	1, 200	677, 981	77, 779	51.3	0	104	32	27	47	16	18	8
**GMA4** ^**b**^ ^**e**^ ^**d**^	1, 981	716, 641	45, 537	66.4	1	68	61	22	11	6	40	31
**GMA5** ^**b**^ ^**f**^ ^**d**^	6, 793	930, 480	17, 562	66.4	0	28	24	14	4	11	28	13
**GMA6** ^**a**^ ^**c**^	247	55, 269	83, 324	58.2	0	115	109	38	68	1	20	3
**GMA7** ^**b**^ ^**c**^	1, 603	947, 843	73, 419	56.6	1	101	32	23	5	18	14	5
**GRU3** ^**b**^ ^**e**^ ^**d**^	520	89, 131	17, 727	66.5	0	26	23	12	6	3	42	16
**GTE6** ^**a**^ ^**c**^	915	141, 321	56, 982	67.8	0	86	86	23	49	3	252	87
**GTE8** ^**b**^ ^**c**^	1, 605	777, 336	67, 617	66.0	0	94	67	23	22	5	48	36

^a^ sequenced using 454,

^b^ sequenced using Illumina,

^c^ reads assembled using CLC workbench (v6.5.1),

^d^ reads assembled using CLC workbench (v7.5.1),

^e^ reads assembled using Spades (v3.1.0),

^f^ reads assembled using ABySS (v1.3.7).

Most of these phage genomes contained no putative tRNAs, and no tmRNA could be identified in any of them (Table 3). Of those phages where tRNAs were seen, phage GMA7 contained 1 putative tRNA-Asn, GMA4 contained 1 putative tRNA-Trp, and phage GMA2 contained a tRNA cluster of 57, 883 to 60, 154 bp where 16 putative tRNA were located (S2 Table). Such tRNA clusters have been observed previously in *Mycobacterium* phages where they appear to be important in late lytic growth, where they may compensate for degradation and inadequacy of host tRNA [20].

The assembled DNA sequence of all nine phages were compared to one another and to other sequences deposited in public databases. Two phage’s isolated in this study, GMA4 and GMA5, were 77% homologous, suggesting a close evolutionary relationship. The genome sequence of GMA7 shared a 97% homology to the DNA sequence in phage GTE7, a polyvalent *Gordonia* phage [12]. Similarly, phage GTE8 shared a lower level of homology (81% and 83% respectively) to two closely related *Gordonia* phages GTE5 and GRU1 described previously [13]. The remainder of the genomes were substantially different to all other phage sequences deposited in GenBank.

### Evidence for potential spontaneous prophage induction events

Whole genome sequencing using next generation DNA sequencing technology allows a high level of genome coverage. During the phage isolation procedure, GMA3, GMA4 and GMA5 were isolated in the same host, *G*. *malaquae* (BEN700). After multiple rounds of purification, the phages were grown to high titre and DNA was isolated for sequencing. Upon sequencing and assembly, it was clear that the phage contigs obtained had >1200-fold coverage and a smaller contaminating contig appears in all three isolations of approximately 41 kb with a lower coverage, 17-fold to 227-fold (Table 4). PCR analysis of the genomic DNA of *G*. *malaquae* (Ben700) revealed the contaminating contig was present in the host presumably as a prophage and therefore named GMA1.

**Table 4:** Coverage of phage GMA1 in the assemblies of phages GMA3, GMA4 and GMA5.

Phage Sequenced	Length of contaminating contig	Average coverage	Total reads
GMA3	41,106	17-fold	5,097
GMA4	40,897	227-fold	70,089
GMA5	41,106	32-fold	10,135

This observation suggests that this putative lysogenic *G*. *malaquae* strain could tolerate co-infection with these three phages, as well as the previously described GTE2 phage [11]. Whether these phages interact while co-infecting is unknown.

### Sequence repeats in *Gordonia* phage genomes

Repeat structures have been reported previously in genome sequences of several related phages [13–14]. All nine phage genomes contain between 1 to 18 palindromic sequences of between 14 to 98 bp in length (S3 Table). Some of these are located in what appear to be intergenic areas, which might support their roles as putative *rho*-independent transcriptional terminators [21]. They also contained 14 to 252 direct repeats ranging in length between 14 and 425 bp (S4 Table). Also seen in these genomes were 3 to 87 inverted repeats of 56 to 14 bp long (S4 Table). Inverted repeats may indicate replication origins and transposable elements [22], but neither of these could be identified in any of these phages, and so their roles remain unknown.

### *Gordonia* phage DNA packaging modules

In *Siphoviridae* phage genomes, the large terminase subunit protein usually functions in a complex with a small terminase subunit, and together these act to mediate cleavage of the phage DNA at specific sites prior to packaging into the prohead [23–24]. The gene encoding the large terminase subunit was identified in all nine *Gordonia* phages examined here by either amino acid sequence homology to other known terminase genes (GMA4, GMA3, GMA5, GMA7, and GTE8) or the presence of the diagnostic conserved motif. In *Siphoviridae* phages the small terminase gene is typically located upstream, and is transcribed in the same direction as the large terminase [23–24]. In all phages except GMA2 this pattern could be recognized, and in some cases supported by amino acid sequence homology to other known small terminases (S1 Table).

### *Gordonia* phage structural protein genes and their proteomics

Phage structural protein genes are located typically adjacent to the DNA packaging module, usually beginning with head morphogenesis genes, followed by tail morphogenesis genes [19]. Some departures from this gene arrangement were seen in these nine phages. For example, in GMA6, only one (*orf8*) of the genes identified between the terminase genes (*orf2* and *orf3*) and the putative portal protein gene (*orf11*), could be assigned a putative function in encoding a nucleoside triphosphate pyrophosphohydrolase. This interpretation was based on the presence of the cd11541 motif. Seemingly involved in DNA maintenance, it was seen between the structural and packaging modules, a location different to the typical modulated genome architecture of *Siphoviridae* phages, where all genes of similar function are clustered together [25]. Furthermore, *orf14*, within the structural gene module of GMA6, appears to encode a HNH endonuclease based on its amino acid sequence homology to the diagnostic pfam01844 motif. Gene arrangements in phage GMA2 suggest that *orf21*, encoding a putative DNA methyltransferase is in the structural gene module.

In all nine phages the tape measure proteins were encoded by their longest gene, which is usual in *Siphoviridae* phages [19] (S1 Table, Fig 3). The only exception was in phage GRU3 where *orf6* encoding a putative phage head protein was slightly larger in size (657 amino acids) than *orf12* encoding its tape measure protein, 622 amino acids long. In most of these (GMA2, GMA3, GMA4, GMA6, GMA7, and GTE8), the two genes preceding that encoding the tape measure protein were identified as encoding putative tail assembly proteins, where the latter appeared to be translated using a conserved programmed frameshift, a common feature of *Siphoviridae* phages [26]. Usually the gene immediately upstream of these is that encoding the putative major tail protein [26].

Mass spectrometry data (S5 Table) seemed to suggest several structural genes are located outside the structural gene module. For example, in phage GMA4 *orf66* was located in the DNA replication module, with a translated protein sequence homologous to a hypothetical protein from *Aeromicrobium marinum*, but also a motif for a phage tail fiber protein (COG5301). Similarly, in GMA6, *orf43* was located within the DNA replication gene modules.

### *Gordonia* phage lysis gene modules are diverse

Lysin genes were identified in all nine *Gordonia* phages, but their locations and numbers varied, and as with many already discussed, they often appeared to disrupt the usual and expected modular genome architecture of *Siphoviridae* phages. Phages GMA5 and GRU3 contain a D-alanyl-D-alanine carboxypeptidase encoding gene (*orf5* in both) showing amino acid sequence homology to a hypothetical protein in *Gordonia soli*, located within what appears to be the phage structural module (S1 Table, Fig 3). A phage lysin motif within the structural gene module was reported for *Rhodococcus* phage RRH1, suggesting this is not an uncommon occurrence [27, 28].

In the genomes of GMA7 and GTE6 their lysin genes were adjacent to their structural protein encoding genes (*orf28 –orf29* and *orf38*, respectively), and unusually, both had additional lysin genes in their DNA replication gene modules (*orf41* and *orf58*, respectively). The same pattern was reported for phage GTE7, [12], to which GMA7 is genetically similar at a nucleotide level (97% identity, 95% coverage).

Phages GMA2, GMA3, and GMA6 also had unusual lysin gene arrangements, with higher numbers of such genes than the more common lysin A and B arrangement [29]. Phage GMA2 unusually possessed four putative lysin genes (*orf35 –orf38*), identified from their amino acid homologies and presence of the diagnostic pfam13529 (peptidase), pfam01510 (N-acetylmuramoyl-L-alanine amidase), and cd02619 (peptidase) motifs in Orf35, Orf36, and Orf38, respectively. Phage GMA6 also had four lysin genes (*orf34*, *orf37*, *orf40*, and *orf45*), many of which were separated by genes associated with DNA replication/maintenance and virion morphogenesis. A similar pattern was seen in phage GMA3, which contained three putative lysin genes (*orf22*, *orf24*, and *orf26*) separated by a putative nuclease gene (*orf25*), again associated with DNA replication/maintenance (S1 Table, Fig 3).

*Orf45* in GMA6 is a more complex lysin gene than those seen in all other actinophages. It alone encodes an unusually high number of different lysin motifs. These include an N-terminal BacA motif of a bacterial lysin from *Enterococcus faecalis* (cd06418), an N-acetylmuramoyl-L-alanine amidase motif (pfam01510) downstream of this, a peptidase motif (pfam01551) further downstream, and an additional C-terminal motif (pfam13810) of unknown function (S1 Table).

Holins could not be identified in phages GMA4, GMA5, and GRU3 by nucleotide or amino acid sequence homologies, nor by the criteria of Wang et al. [30], which state that expressed products should be less than 150 amino acid residues and contain two or more transmembrane regions. If holins are present in these two phages, it would seem that their genes are novel in their locations and/or translated amino acid sequences.

### DNA replication/maintenance genes

DNA replication modules in all other actinophage genomes sequenced so far are arranged in a modular architecture, where genes functioning in DNA replication/maintenance are located adjacent to lysin genes [11–14, 27, 28, 31, 32]. In GMA4, GMA7, and GMA2 phages, this region contains putative DNA-methylase encoding genes, of these GMA2 appears to possess at least two (*orf21* and *orf51*). If functional, they may play a role in protecting their DNA from host cell restriction attack [33]. Such enzymes have been identified in other *Gordonia* phages including GTE7 phages [12]. Metagenomic studies by Tamaki et al. [34] have suggested that methylase genes are more prevalent in phages within the activated sludge habitat from where most actinophages have come. Glycosyltransferase encoding genes are also seen in many phage genomes [35], including that of GMA2 (*orf4* and *orf22*), and all appear to have similar functions to phage methylases where they help protect phage DNA from digestion with restriction endonucleases from host RM systems [33]. These genes can also have other functions including serotype conversion in temperate phages [35], and so their purpose here remains unclear.

### Lysogeny and lysogenic conversion genes

Genomes of phages GMA3, GMA4, GMA5, and GRU3 all contain putative genes that are homologues of phage integrase genes (*orf75*, *orf29*, *orf17*, and *orf17* respectively), based on their product amino acid sequence similarities to those of known phage integrases, and the possession of the integrase specific motif pfam00589. If functional, their presence suggests the capability for a lysogenic lifecycle as well as a lytic one.

The GMA4 genome appears to encode several moron genes that may confer a selective advantage to its host. For example, it possesses a gene associated with phage resistance (*orf34*) [36]. The N-terminal region of Orf34 contains a Rha motif (pfam09669), thought to interfere with further phage infection of bacterial host strains lacking the integration host factor (IHF) [36]. It regulates expression of the *rha* gene, and so may confer resistance to further phage attack of any bacterial host infected by it in a lysogenic cycle [36].

### Unexpected features of the *Gordonia* phages

As mentioned, most of the nine *Gordonia* phages sequenced in this study had highly distinctive genomes, with high percentages of ORFans (5 to 59%) (S1 Table), for which no statistically significant identifications could be made against sequences held in GenBank. Yet their genes encode motifs suggestive of their putative function. For example, both GMA2 and GMA6 possess a cd00233 motif in their Orf14 and Orf13 putative proteins, respectively. The *orf14* and *orf13* genes appear to encode a VIP2 family actin-ADP-ribosylating toxin with high specificity against the insect pest corn rootworms, and sharing a statistically significant sequence similarity with enzymatic components of other binary toxins, including the *Clostridium botulinum* C2 toxin, *C*. *perfringens* iota toxin, *C*. *piroform*e toxin, *C*. *piroforme* toxin and *C*. *difficile* toxin [37].

Furthermore, phage GTE6 genome appeared to contain a gene (*orf12*), encoding a host cell surface-exposed lipoprotein since its expressed amino acid sequence shares homology with the pfram07553 motif. Such motifs are usually involved in superinfection exclusion, acting at the stage of DNA release from the phage head into the host cell. These motifs have been associated with Superinfection exclusion (Sie) systems in temperate phages, where they interfere with co-infections involving other phages [33, 38, 39]. Presence of such a motif in what appears to be an obligatory lytic phage is unexpected. Equally unexpected is that *orf21* in phage GTE6 encodes a putative Eppstein-Barr nuclear antigen (Orf21), showing 35% amino acid sequence similarity to that of *Saccharomonospora* phage PIS 136 in this region [40]. Whether this homology reflects a similar function for the pair, or a distant evolutionary relationship between them, is unknown. The *orf4* of phage GMA7 also appears to encode an unexpected motif (cd12820) normally associated with a putative adhesion virulence factor, forming a matrix on the bacterial outer membrane, which mediates binding to collagen and epithelial cells [41].

### Evolutionary ancestry of *Gordonia* phages

From the data presented here, it is clear that phages GTE8, isolated from creek water and GMA7, from activated sludge are genetically very similar to phages GTE5/GRU1, and GTE7, respectively. It is reasonable to propose that these similarities reflect a closely shared ancestral past. Similar comments apply to phage GMA5 and GRU3. Despite not sharing nucleotide sequence identity with phage GTE7 DNA, the expressed amino acid sequences of phage GMA3 expressed genes are highly similar to it. Nine genes of GMA3 were most similar to those from GTE7, while 23 other genes were most similar to those from phage ReqiDocB7, to which GTE7 genome is closely related at an amino acid level [12, 42]. As a similar closeness was not reflected at the nucleotide sequence level, one suggestion might be that more distant evolutionary relationships exist between GTE7, ReqiDocB7, and GMA3. GMA3 contains a gene showing homology to a putative RNA-binding gene from the chimpanzee *Pan troglodytes* (*orf91*), and a centromere protein F-like gene from the banana plant *Musa acuminata* (*orf74*). The expected values for these matches are borderline statistically significant (2e-04 and 6e-04, respectively), so whether these data reflect real distant evolutionary relationships remains unresolved.

Each individual *Gordonia* phage genome sequence was unique, but given the close genetic relationships between the Mycolata host genera, attempts were made to classify these according to the system of Hatfull, Jacobs-Sera [43] designed to show evolutionary relationships the Mycobacteriophages. It was not possible to place these *Gordonia* phages into any of their pre-existing clusters. For example, while GMA7 is highly similar to GTE7 at a nucleotide sequence level, and GMA3 contains genes encoding several putative proteins also similar to those of GTE7, none could be grouped with any Mycobacteriophages. Instead they emerge as singletons since none of the existing clusters embraced them.

### Suitability of these phages for use in foam bio-control

Of the nine phages examined in this study, GMA3, GMA5, GMA5 and GRU3 contain putative integrase genes, suggesting that they may undertake a lysogenic lifecycle. If these genes are functional, then these are probably undesirable candidates for standard phage therapy for activated sludge foam control.

Of the remaining five phages GMA2 and GMA6 both appear to contain a putative VIP2 family actin-ADP-ribosylating toxin gene, which target eukaryotic proteins upon infection. Consequently, neither phage would be considered being suitable for bio-control strategies. Their release into the environment may potentially result in the spread of these undesirable genes and an increased virulence of other bacteria hosts.

Other *Gordonia* phages GMA7, GTE6, and GTE8 appear to be obligatory lytic. Of these, GMA7 and GTE8 seem particularly attractive as both have impressive broad host ranges. For instance, GMA7 targets eleven strains of *Gordonia* including those of *G*. *terrae*, *G*. *malaquae*, *G*. *rubropertincta and G*. *hydrophobica*. Similarly, GTE8 targets several species including *G*. *terrae* (CON34, GOR9, G232) *and G*. *rubropertincta* (CON38) and *Nocardia asteroids* (strain CON12) (Table 1). Furthermore, phage GMA7 contains a putative DNA methylase gene (*orf38*) containing a pfam01555 motif. If this gene is functional, then this phage may evade cleavage by host defense RM systems [33, 38] and thus become an even more powerful addition to any phage therapy cocktail.

## Conclusions

Nine phages infective for members of the genus *Gordonia* were isolated from wastewater and natural water environments, several of which had broad host ranges. Methods for visualization of the phage infection cycle using TEM were successful and may prove to be useful in studies of mechanisms of phage infection. Whole genome sequencing of these phages revealed that their genomes were all distinctively different, failing to cluster with those of known Mycobacteriophages, based on both nucleotide and amino acid sequence similarities. Some are less modular in their genomic architecture than those characterized previously, and contain a higher number of lysin genes seen in other Actinophage genomes previously. Of these nine phages, three broad host range phages GMA7, GTE6, and GTE8 appear obligatory lytic and hence potentially suitable candidates for phage therapy cocktails to control activated sludge foaming.

## Supporting Information

S1 Table Genome annotations of nine phages infecting *Gordonia* spp. ^a^ ORFs were numbered consecutively, ^b^ The most closely related gene (only if named) and the name of the organism, ^c^ Percentage identity is based on the best match when a BLAST P analysis is performed, ^d^ The probability of obtaining a match by chance as determined by BLAST analysis. Only values less than 10^−4^ were considered significant, ^e^ Predicted function is based on amino acid identity, conserved motifs, and gene location within functional modules.
(DOCX)

S2 Table Putative tRNA detected in *Gordonia* phage genomes. (DOCX)

S3 Table Palindromes in the genome sequence of nine *Gordonia* spp. phages. (DOCX)

S4 Table Repeats in the genome sequences of nine *Gordonia* spp. phages. I indicates inverted repeat, D indicates direct repeat.
(DOCX)

S5 Table Summary of *Gordonia* phage structural genes identified by mass spectroscopy. (DOCX)
