# Supplementary material for: Lysis to Kill: Evaluation of the Lytic Abilities, and Genomics of Nine Bacteriophages Infective for Gordonia spp. and Their Potential Use in Activated Sludge Foam Biocontrol
Source: PLoS One. 2015 Aug 4;10(8):e0134512. doi: 10.1371/journal.pone.0134512 (PMC4524720; doi:10.1371/journal.pone.0134512)
Supplement: S1 Table — a ORFs were numbered consecutively, b The most closely related gene (only if named) and the name of the organism, c Percentage identity is based on the best match when a BLAST P analysis is performed, d The probability of obtaining a match by chance as determined by BLAST analysis. Only values less than 10−4 were considered significant, e Predicted function is based on amino acid identity, conserved motifs, and gene location within functional modules. (DOCX) [file pone.0134512.s001.docx]

| ORF ^a^ | Coordinates | Size (aa) | Significant match ^b^ | % identity ^c^ | E_0_ value ^d^ | Putative function ^e^ |
| --- | --- | --- | --- | --- | --- | --- |
| GMA2-*orf1* | 138..1817 | 560 | ATP-binding protein [*Kitasatospora mediocidica*] | 44 | 4e-90 | Terminase (pfam03237) |
| GMA2-*orf2* | 1862..3583 | 574 | - | - | - | - |
| GMA2-*orf3* | 3580..3978 | 133 | - | - | - | - |
| GMA2-*orf4* | 3971..4774 | 268 | hypothetical protein [*Geobacter bremensis*] | 24 | 4e-08 | Glycotransferase (pfam04724) |
| GMA2-*orf5* | 4814..5041 | 76 | - | - | - | - |
| GMA2-*orf6* | 5078..5734 | 219 | hypothetical protein [*Xanthomonas vasicola*] | 30 | 6e-10 | Mu protein F (pfam04233) |
| GMA2-*orf7* | 5731..5916 | 62 | - | - | - | - |
| GMA2-*orf8* | 5906..6217 | 104 | sporulation protein [*Bacillus* sp. 72] | 34 | 5e-05 | Unknown (pfam07098) |
| GMA2-*orf9* | 6225..7979 | 585 | hypothetical protein [*Frankia alni*] | 31 | 9e-51 | Phage structural protein |
| GMA2-*orf10* | 7981..10632 | 884 | hypothetical protein [*Rhodococcus fascians*] | 38 | 5e-15 | Phage structural protein |
| GMA2-*orf11* | 10696..12123 | 476 | hypothetical protein [*Streptomyces* sp. NRRL F-5135] | 55 | 1e-18 | - |
| GMA2-*orf12* | 12206..13207 | 334 | hypothetical protein [*Frankia alni*] | 50 | 8e-53 | - |
| GMA2-*orf13* | 13226..14263 | 346 | - | - | - | - |
| GMA2-*orf14* | 14328..15059 | 244 | - | - | - | VIP2; A family of actin-ADP-ribosylating toxin (cd00233) |
| GMA2-*orf15* | 15052..15183 | 44 | - | - | - | - |
| GMA2-*orf16* | 15295..16959 | 555 | mycobacteriophage protein [*Frankia alni*] | 40 | 6e-64 | Phage structural protein |
| GMA2-*orf17* | 17011..17706 | 232 | MULTISPECIES: hypothetical protein [*Actinomycetales*] | 45 | 9e-60 | - |
| GMA2-*orf18* | 17722..18366 | 215 | hypothetical protein [*Frankia alni*] | 33 | 1e-10 | - |
| GMA2-*orf19* | 18455..19078 | 208 | hypothetical protein [*Streptosporangium amethystogenes*] | 29 | 2e-08 | - |
| GMA2-*orf20* | 19143..19571 | 143 | - | - | - | - |
| GMA2-*orf21* | 19657..20244 | 196 | glycosyltransferase/methyltransferase [*Mycobacterium* phage Llama] | 56 | 9e-60 | Methyltransferase (pfam13578) |
| GMA2-*orf22* | 20237..20953 | 239 | hypothetical protein [*Mycobacterium* sp. UM_RHS] | 53 | 5e-84 | Glycosyl transferase (pfam00535) |
| GMA2-*orf23* | 21038..21652 | 205 | glycosyltransferase [*Mycobacterium* phage CaptainTrips] | 37 | 5e-30 | - |
| GMA2-*orf24* | 21753..22253 | 167 | - | - | - | - |
| GMA2-*orf25* | 22256..22675 | 140 | hypothetical protein [*Streptomyces* sp. SPB74] | 42 | 2e-09 | Putative major tail protein |
| GMA2-*orf26* | 22675..23118 | 148 | - | - | - | Putative tail assembly protein |
| GMA2-*orf27* | 23100..23402 | 101 | - | - | - | Putative tail assembly protein translated by conserved programmed translational frameshift |
| GMA2-*orf28* | 23414..30640 | 2409 | unnamed protein product [*Rhodococcus* phage REQ2] | 39 | 1e-100 | Tape measure protein (pfam03280; pfam13514; COG5412) |
| GMA2-*orf29* | 30651..31595 | 315 | hypothetical protein [*Rhodococcus fascians*] | 34 | 2e-46 | - |
| GMA2-*orf30* | 31614..33245 | 544 | hypothetical protein [*Rhodococcus fascians*] | 49 | 6e-171 | - |
| GMA2-*orf31* | 33254..33889 | 212 | hypothetical protein [*Rhodococcus fascians*] | 49 | 9e-12 | - |
| GMA2-*orf32* | 33886..36579 | 898 | hypothetical protein [*Gordonia sihwensis*] | 27 | 3e-55 | - |
| GMA2-*orf33* | 36581..38293 | 571 | hypothetical protein [*Gordonia soli*] | 70 | 1e-42 | - |
| GMA2-*orf34* | 38290..39237 | 316 | - | - | - | - |
| GMA2-*orf35* | 39327..40022 | 232 | hypothetical protein [*Streptomyces* sp. SM8] | 43 | 3e-40 | Lysin/Peptidase (pfam13529) |
| GMA2-*orf36* | 40019..40747 | 243 | hypothetical protein [*Nocardia otitidiscaviarum*] | 41 | 7e-39 | Lysin (pfam01510) |
| GMA2-*orf37* | 40766..41137 | 124 | N-acetylmuramoyl-L-alanine amidase [*Rhodococcus opacus*] | 49 | 1e-08 | Putative lysin |
| GMA2-*orf38* | 41134..41802 | 223 | papain cysteine protease family protein [*Mycobacterium xenopi* 3993] | 50 | 1e-64 | Lysin/Peptidase (cd02619) |
| GMA2-*orf39* | 41812..42174 | 121 | holin [*Mycobacterium* phage Milly] | 36 | 1e-15 | Putative holin |
| GMA2-*orf40* | 42184..42681 | 166 | hypothetical protein [*Rhodococcus fascians*] | 36 | 2e-11 | - |
| GMA2-*orf41* | 42741..43205 | 155 | hypothetical protein GTE7_gp030 [*Gordonia* phage GTE7] | 35 | 3e-10 | - |
| GMA2-*orf42* | 43216..43593 | 126 | - | - | - | - |
| GMA2-*orf43* | complement(43696..44082) | 129 | - | - | - | - |
| GMA2-*orf44* | complement(44075..44773) | 233 | thymidylate synthase, flavin-dependent [*Corynebacterium striatum* ATCC 6940] | 57 | 1e-84 | Thymidylate synthase (pfam02511) |
| GMA2-*orf45* | complement(44898..47093) | 732 | hypothetical protein [*Salinispora pacifica*] | 30 | 2e-24 | Cobalmin biosynthesis (pfam06213) |
| GMA2-*orf46* | complement(47109..47861) | 251 | - | - | - | - |
| GMA2-*orf47* | complement(47995..48456) | 154 | unnamed protein product [*Gordonia* phage GRU1] | 33 | 7e-11 | - |
| GMA2-*orf48* | complement(48474..50507) | 678 | ATPase AAA [*Amycolatopsis thermoflava*] | 40 | 3e-54 | AAA protein (pfam07728) |
| GMA2-*orf49* | complement(50611..51057) | 149 | WhiB family transcriptional regulator [*Corynebacterium callunae*] | 37 | 5e-07 | Whib (pfam02467) |
| GMA2-*orf50* | complement(51091..51465) | 125 | - | - | - | - |
| GMA2-*orf51* | complement(51538..52830) | 431 | M2.BsmFI [*Geobacillus stearothermophilus*] | 22 | 5e-06 | C-5 cytosine specific methylase (pfam00145) |
| GMA2-*orf52* | complement(52827..53375) | 183 | deoxycytidine-triphosphatase [*Bacillus subtilis*] | 35 | 1e-05 | dUTPase (pfam08761) |
| GMA2-*orf53* | complement(53525..54511) | 329 | - | - | - | - |
| GMA2-*orf54* | complement(54513..55700) | 396 | - | - | - | Glycosyltransferase (COG0438) |
| GMA2-*orf55* | complement(55700..56095) | 132 | hypothetical protein GTE7_gp062 [*Gordonia* phage GTE7] | 32 | 2e-04 | - |
| GMA2-*orf56* | complement(56092..57162) | 357 | - | - | - | - |
| GMA2-*orf57* | complement(57205..57738) | 178 | - | - | - | GIY-YIG (cd10443) |
| GMA2-*orf58* | complement(60616..60747) | 44 | - | - | - | - |
| GMA2-*orf59* | complement(60935..62569) | 545 | RNA-binding protein [*Streptomyces carneus*] | 53 | 0.0 | TROVE (pram05731) |
| GMA2-*orf60* | complement(62877..63482) | 202 | hypothetical protein [*Vibrio* phage VpKK5] | 31 | 1e-04 | Unknown (pfam05037) |
| GMA2-*orf61* | complement(63565..64314) | 250 | hypothetical protein [*Caldanaerobius polysaccharolyticus*] | 23 | 7e-11 | AAA protein (13479) |
| GMA2-*orf62* | complement(64418..65413) | 332 | - | - | - | Nuclease (pfam12705) |
| GMA2-*orf63* | complement(65506..65715) | 70 | - | - | - | - |
| GMA2-*orf64* | complement(65812..67776) | 655 | possible DNA helicase [*Aeromicrobium marinum*] | 26 | 2e-47 | Helicase (COG0553) |
| GMA2-*orf65* | complement(67844..68347) | 168 | - | - | - | - |
| GMA2-*orf66* | complement(68344..70128) | 595 | gp44 [*Mycobacterium* phage Bxz2] | 31 | 3e-68 | DNA polymerase I (COG0749) |
| GMA2-*orf67* | complement(70132..70752) | 207 | hypothetical protein [*Arthrobacter* sp. 135MFCol5.1] | 33 | 1e-10 | Nuclease (cd10443; cd00283) |
| GMA2-*orf68* | complement(70884..71072) | 63 | - | - | - | - |
| GMA2-*orf69* | complement(71187..72845) | 553 | - | - | - | - |
| GMA2-*orf70* | complement(72861..73346) | 162 | cell division protein DedD [*Streptomyces* sp. NRRL F-2580] | 37 | 2e-19 | Deamimase (pfam00383) |
| GMA2-*orf71* | complement(73339..73629) | 97 | (2Fe-2S)-binding protein [*Rhodococcus opacus*] | 36 | 3e-10 | - |
| GMA2-*orf72* | complement(73712..74554) | 281 | methyltransferase [*Streptomyces* sp. NRRL S-340] | 43 | 3e-23 | Thymidylate synthase (pfam00303) |
| GMA2-*orf73* | complement(74572..75363) | 264 | - | - | - | - |
| GMA2-*orf74* | complement(75371..75958) | 196 | hypothetical protein ACD_80C00175G0001 [uncultured bacterium (gcode 4)] | 38 | 1e-04 | Endonuclease (pfam01844) |
| GMA2-*orf75* | complement(76020..76604) | 195 | - | - | - | - |
| GMA2-*orf76* | complement(76664..77386) | 241 | hypothetical protein [*Gordonia soli*] | 38 | 5e-15 | - |
| GMA2-*orf77* | complement(77394..77513) | 40 | - | - | - | - |
| GMA2-*orf78* | complement(77510..78064) | 185 | - | - | - | - |
| GMA2-*orf79* | complement(78057..78731) | 225 | unnamed protein product [*Rhodococcus* phage REQ2] | 44 | 3e-40 | Phosphoesterase (COG4186) |
| GMA2-*orf80* | complement(78728..79051) | 108 | - | - | - | - |
| GMA2-*orf81* | complement(79048..79308) | 87 | - | - | - | - |
| GMA2-*orf82* | complement(79524..79829) | 102 | hypothetical protein [*Mycobacterium abscessus*] | 43 | 1e-08 | - |
| GMA2-*orf83* | complement(79814..80008) | 65 | - | - | - | - |
| GMA2-*orf84* | complement(80010..80351) | 114 | hypothetical protein [*Rhodococcus fascians*] | 69 | 3e-45 | - |
| GMA2-*orf85* | complement(80348..80551) | 68 | - | - | - | - |
| GMA2-*orf86* | complement(80749..81003) | 85 | - | - | - | - |
| GMA2-*orf87* | complement(81000..81263) | 88 | - | - | - | - |
| GMA2-*orf88* | complement(81347..81784) | 146 | MULTISPECIES: hypothetical protein [*Micrococcineae*] | 53 | 2e-38 | - |
| GMA2-*orf89* | complement(81781..81942) | 54 | - | - | - | - |
| GMA2-*orf90* | complement(82002..82721) | 240 | hypothetical protein [*Gordonia malaquae*] | 64 | 4e-30 | - |
| GMA2-*orf91* | complement(82725..83294) | 190 | - | - | - | - |
| GMA2-*orf92* | complement(83392..83565) | 58 | - | - | - | - |
| GMA2-*orf93* | complement(83562..84947) | 462 | DNA primase [*Fervidicella metallireducens* AeB] | 32 | 8e-11 | Bifunctional DNA primase/polymerase (pfam09250) |
| GMA2-*orf94* | complement(85005..87089) | 695 | hypothetical protein [*Escherichia coli*] | 25 | 5e-10 | Unknown (pfam13148) |
| GMA2-*orf95* | complement(87170..87367) | 66 | hypothetical protein [*Hellea balneolensis*] | 48 | 2e-08 | HTH DNA binding (pfam12728) |
| GMA2-*orf96* | complement(87394..87714) | 107 | - | - | - | - |
| GMA2-*orf97* | complement(88763..88972) | 70 | - | - | - | - |
| GMA2-*orf98* | complement(89408..89671) | 88 | - | - | - | - |
| GMA2-*orf99* | complement(89680..90450) | 257 | hypothetical protein TPA2_gp53 [*Tsukamurella* phage TPA2] | 48 | 3e-20 | - |
| GMA2-*orf100* | complement(91125..91364) | 80 | - | - | - | - |
| GMA2-*orf101* | complement(91583..91927) | 115 | - | - | - | - |
| GMA2-*orf102* | complement(91934..92176) | 81 | - | - | - | - |
| GMA2-*orf103* | complement(92154..92477) | 108 | hypothetical protein [*Mycobacterium marinum*] | 51 | 1e-15 | - |
| GMA2-*orf104* | complement(92474..92803) | 110 | hypothetical protein HMPREF1211_07474 [Streptomyces sp. HGB0020] | 39 | 1e-06 | - |
| GMA2-*orf105* | complement(92784..93677) | 298 | - | - | - | Chromosome segregation ATPase (COG1196) |
| GMA2-*orf106* | complement(93680..93946) | 89 | hypothetical protein PBI_LLAMA_56 [*Mycobacterium* phage Llama] | 42 | 2e-04 | - |
| GMA2-*orf107* | complement(93939..94211) | 91 | gp49 [*Mycobacterium* phage PMC] | 49 | 2e-13 | - |
| GMA2-*orf108* | complement(94208..94429) | 74 | - | - | - | - |
| GMA2-*orf109* | complement(94426..94866) | 147 | - | - | - | - |
| GMA2-*orf110* | complement(94967..95407) | 147 | gp056 [*Rhodococcus* phage ReqiDocB7] | 34 | 3e-05 | - |
| GMA2-*orf111* | complement(95493..95606) | 38 | - | - | - | - |
| GMA2-*orf112* | complement(95579..97174) | 532 | ADP-ribosylation/Crystallin J1 [*Mycobacterium rhodesiae*] | 44 | 6e-26 | ADP-ribosylglycohydrolase (pfam03747) |
| GMA2-*orf113* | complement(97225..97428) | 68 | - | - | - | - |
| GMA2-*orf114* | complement(97531..97956) | 142 | - | - | - | - |
| GMA2-*orf115* | complement(97985..98689) | 235 | - | - | - | Nucleotideyltransferase (pfam01909) |
| GMA2-*orf116* | complement(98704..98919) | 72 | - | - | - | - |
| GMA2-*orf117* | complement(98912..99322) | 137 | - | - | - | - |
| GMA2-*orf118* | complement(99358..99921) | 188 | - | - | - | Whib (pfam02467; pfam07900) |
| GMA2-*orf119* | complement(99959..100168) | 70 | - | - | - | - |
| GMA2-*orf120* | complement(100181..100528) | 116 | - | - | - | - |
| GMA2-*orf121* | complement(100688..100930) | 81 | - | - | - | - |
| GMA2-*orf122* | complement(100931..101578) | 216 | hypothetical protein BADFISH_56 [*Mycobacterium* phage Badfish] | 28 | 6e-09 | - |
| GMA2-*orf123* | complement(101652..102011) | 120 | - | - | - | - |
| GMA2-*orf124* | complement(102069..102752) | 228 | - | - | - | - |
| GMA2-*orf125* | complement(102784..103044) | 87 | - | - | - | - |
| GMA2-*orf126* | complement(103105..103344) | 80 | - | - | - | - |
| GMA3-*orf1* | 105..1739 | 545 | putative small terminase [*Gordonia* phage GTE7] | 30 | 2e-16 | Putative small terminase subunit |
| GMA3-*orf2* | 1740..4886 | 1049 | TerL [*Rhodococcus* phage ReqiDocB7] | 48 | 1e-112 | Large terminase subunit (cd01335) |
| GMA3-*orf3* | 4995..6896 | 634 | Mu gp29-like protein [*Rhodococcus* phage ReqiDocB7] | 38 | 1e135 | Unknown (pfam06074) |
| GMA3-*orf4* | 6899..7189 | 97 | - | - | - | - |
| GMA3-*orf5* | 7211..8362 | 384 | gp010 [*Rhodococcus* phage ReqiDocB7] | 42 | 3e-70 | - |
| GMA3-*orf6* | 8376..8843 | 156 | gp011 [*Rhodococcus* phage ReqiDocB7] | 43 | 2e-32 | - |
| GMA3-*orf7* | 8860..10044 | 395 | putative major capsid protein [*Gordonia* phage GTE7] | 46 | 3e-108 | Phage major capsid protein (pfam03864) |
| GMA3-*orf8* | 10068..10358 | 97 | - | - | - | - |
| GMA3-*orf9* | 10402..11406 | 335 | gp015 [*Rhodococcus* phage ReqiDocB7] | 32 | 1e-12 | Unknown (pfam07030) |
| GMA3-*orf10* | 11406..11906 | 167 | hypothetical protein GTE7_gp013 [*Gordonia* phage GTE7] | 31 | 6e-04 | - |
| GMA3-*orf11* | 11907..12410 | 168 | gp017 [*Rhodococcus* phage ReqiDocB7] | 33 | 8e-18 | - |
| GMA3-*orf12* | 12429..12743 | 105 | - | - | - | - |
| GMA3-*orf13* | 12749..13774 | 342 | gp019 [*Rhodococcus* phage ReqiDocB7] | 49 | 2e-104 | Putative major tail protein |
| GMA3-*orf14* | 13894..14499 | 202 | pre-TMP frameshift protein [*Rhodococcus* phage ReqiDocB7] | 35 | 4e-25 | Putative tail assembly protein |
| GMA3-*orf15* | 14481..14756 | 92 | truncated pre-TMP frameshift protein [*Rhodococcus* phage ReqiDocB7] | 36 | 1e-07 | Putative tail assembly protein translated by conserved programmed translational frameshift |
| GMA3-*orf16* | 14859..24419 | 3187 | tape measure protein [*Rhodococcus* phage ReqiDocB7] | 34 | 5e-174 | Tape measure protein (pfam10145; pfam12889; COG5412; pfam01464) |
| GMA3-*orf17* | 24422..27004 | 861 | gp023 [*Rhodococcus* phage ReqiDocB7] | 47 | 0.0 | - |
| GMA3-*orf18* | 27013..28752 | 580 | gp024 [*Rhodococcus* phage ReqiDocB7] | 50 | 0.0 | Putative phage tail protein (pfam13550) |
| GMA3-*orf19* | 28749..31493 | 915 | hypothetical protein [*Gordonia sihwensis*] | 28 | 1e-58 | - |
| GMA3-*orf20* | 31495..33216 | 574 | hypothetical protein [*Gordonia soli*] | 64 | 1e-37 | - |
| GMA3-*orf21* | 33213..33800 | 196 | hypothetical protein [*Gordonia soli*] | 39 | 1e-12 | - |
| GMA3-*orf22* | 33881..34582 | 234 | twin-arginine translocation pathway signal [*Mycobacterium phlei*] | 48 | 1e-59 | Lysin (cd06418) |
| GMA3-*orf23* | 34586..34939 | 118 | - | - | - | - |
| GMA3-*orf24* | 34939..35589 | 217 | hypothetical protein [*Rhodococcus fascians*] | 65 | 6e-69 | Putative lysozyme |
| GMA3-*orf25* | 35593..36075 | 161 | hypothetical protein FG87_22005 [*Nocardia* sp. W9851] | 36 | 6e-24 | Nuclease (pfam13392) |
| GMA3-*orf26* | 36068..36415 | 116 | putative peptidase [*Gordonia* phage GTE7] | 46 | 7e-12 | Putative lysin |
| GMA3-*orf27* | 36415..36849 | 145 | hypothetical protein GTE7_gp026 [*Gordonia* phage GTE7] | 48 | 2e-21 | Puative holin |
| GMA3-*orf28* | 36944..37429 | 162 | hypothetical protein [*Rhodococcus fascians*] | 26 | 2e-04 | - |
| GMA3-*orf29* | 37413..37865 | 151 | hypothetical protein [*Salinispora arenicola*] | 29 | 1e-05 | - |
| GMA3-*orf30* | 37862..38239 | 126 | hypothetical protein [*Nocardia araoensis*] | 29 | 1e-04 | - |
| GMA3-*orf31* | 38249..38593 | 115 | - | - | - | - |
| GMA3-*orf32* | 38590..39348 | 253 | hypothetical protein [*Rhodococcus opacus*] | 41 | 1e-45 | PE-PPE (pfam08237) |
| GMA3-*orf33* | complement(39394..39585) | 64 | - | - | - | - |
| GMA3-*orf34* | complement(39572..39685) | 38 | - | - | - | - |
| GMA3-*orf35* | complement(39682..39822) | 47 | - | - | - | - |
| GMA3-*orf36* | complement(39826..40110) | 95 | - | - | - | - |
| GMA3-*orf37* | complement(40113..40325) | 71 | - | - | - | - |
| GMA3-*orf38* | complement(40325..40837) | 171 | hypothetical protein GTE7_gp041 [*Gordonia* phage GTE7] | 47 | 5e-36 | Nucleoside Triphosphate Pyrophosphohydroplase (cd11542) |
| GMA3-*orf39* | complement(40830..41243) | 138 | hypothetical protein GTE7_gp101 [*Gordonia* phage GTE7] | 48 | 2e-30 | - |
| GMA3-*orf40* | complement(41240..41542) | 101 | hypothetical protein F989_02392 [*Acinetobacter parvus* NIPH 1103] | 39 | 6e-07 | - |
| GMA3-*orf41* | complement(41529..41789) | 87 | hypothetical protein PBI_HAWKEYE_80 [*Mycobacterium* phage Hawkeye] | 48 | 2e-14 | - |
| GMA3-*orf42* | complement(41794..42519) | 242 | hypothetical protein [*Rhodococcus fascians*] | 48 | 3e-58 | Thymidylate synthase (pfam02511) |
| GMA3-*orf43* | complement(42516..42854) | 113 | - | - | - | - |
| GMA3-*orf44* | complement(43209..44399) | 397 | gp033 [*Rhodococcus* phage ReqiDocB7] | 30 | 2e-55 | Nuclease (COG2887) |
| GMA3-*orf45* | complement(44400..44606) | 69 | - | - | - | - |
| GMA3-*orf46* | complement(44610..44762) | 51 | gp037 [*Rhodococcus* phage ReqiDocB7] | 43 | 6e-04 | - |
| GMA3-*orf47* | complement(44762..44971) | 70 | - | - | - | - |
| GMA3-*orf48* | complement(44973..45185) | 71 | transcriptional regulator [*Mycobacterium abscessus*] | 40 | 3e-08 | WhiB transcription factor (pfam02467) |
| GMA3-*orf49* | complement(45224..45505) | 94 | - | - | - | - |
| GMA3-*orf50* | complement(45516..46070) | 185 | gp042 [*Rhodococcus* phage ReqiDocB7] | 33 | 2e -10 | Holliday Junction Resolvase/RusA (pfam05866) |
| GMA3-*orf51* | complement(46067..46372) | 102 | - | - | - | - |
| GMA3-*orf52* | complement(46369..46569) | 67 | - | - | - | - |
| GMA3-*orf53* | complement(46542..47669) | 376 | DnaN [*Rhodococcus* phage ReqiDocB7] | 23 | 5e-20 | DNA Polymerase III beta subunit (COG0592) |
| GMA3-*orf54* | complement(47666..48004) | 113 | - | - | - | - |
| GMA3-*orf55* | complement(48010..48480) | 157 | hypothetical protein [*Gordonia otitidis*] | 48 | 1e-27 | Unknown (pfam10686) |
| GMA3-*orf56* | complement(48480..49106) | 209 | oligoribonuclease [*Corynebacterium genitalium*] | 38 | 3e-29 | Nuclease (pfam00929) |
| GMA3-*orf57* | complement(49106..49348) | 81 | - | - | - | HTH DNA binding protein (pfam12728) |
| GMA3-*orf58* | complement(49351..50868) | 506 | helicase [*Rhodococcus* phage ReqiDocB7] | 49 | 4e-162 | Helicase (COG0553) |
| GMA3-*orf59* | complement(50871..51248) | 126 | - | - | - | - |
| GMA3-*orf60* | complement(51248..51466) | 73 | - | - | - | - |
| GMA3-*orf61* | complement(51469..51882) | 138 | hypothetical protein [*Gordonia sihwensis*] | 36 | 7e-12 | Unknown (pfam05305) |
| GMA3-*orf62* | complement(52079..52285) | 69 | - | - | - | - |
| GMA3-*orf63* | complement(52397..53080) | 228 | gp051 [*Rhodococcus* phage ReqiDocB7] | 40 | 2e-21 | - |
| GMA3-*orf64* | complement(53329..53586) | 86 | - | - | - | - |
| GMA3-*orf65* | complement(53600..54439) | 280 | - | - | - | - |
| GMA3-*orf66* | complement(54429..54884) | 152 | hypothetical protein GTE7_gp056 [*Gordonia* phage GTE7] | 29 | 7e-04 | - |
| GMA3-*orf67* | complement(54868..54996) | 43 | - | - | - | - |
| GMA3-*orf68* | complement(54993..55214) | 74 | - | - | - | - |
| GMA3-*orf69* | complement(55207..55536) | 110 | - | - | - | - |
| GMA3-*orf70* | complement(55533..55790) | 86 | - | - | - | - |
| GMA3-*orf71* | complement(55777..55950) | 58 | - | - | - | - |
| GMA3-*orf72* | complement(55952..56224) | 91 | - | - | - | - |
| GMA3-*orf73* | complement(56241..56648) | 136 | PREDICTED: centromere protein F-like [*Musa acuminata* subsp. malaccensis] | 28 | 6e-04 | - |
| GMA3-*orf74* | complement(56641..56823) | 61 | - | - | - | - |
| GMA3-*orf75* | complement(56823..57620) | 266 | recombinase XerC [*Thermococcus* sp. AM4] | 34 | 4e-22 | Integrase (pfam00589) |
| GMA3-*orf76* | complement(57695..58531) | 279 | - | - | - | - |
| GMA3-*orf77* | complement(58541..58870) | 110 | - | - | - | - |
| GMA3-*orf78* | complement(58867..59571) | 235 | gp064 [*Rhodococcus* phage ReqiDocB7] | 34 | 3e-10 | - |
| GMA3-*orf79* | complement(59664..59888) | 75 | hypothetical protein [*Rhodococcus* sp. UNC363MFTsu5.1] | 40 | 1e-08 | - |
| GMA3-*orf80* | complement(59888..60112) | 75 | - | - | - | - |
| GMA3-*orf81* | complement(60109..60351) | 81 | - | - | - | - |
| GMA3-*orf82* | complement(60344..60877) | 178 | - | - | - | - |
| GMA3-*orf83* | complement(60883..61623) | 247 | hypothetical protein [*Gordonia otitidis*] | 37 | 1e-29 | - |
| GMA3-*orf84* | complement(61626..61793) | 56 | - | - | - | - |
| GMA3-*orf85* | complement(61762..62001) | 80 | - | - | - | - |
| GMA3-*orf86* | complement(61991..62260) | 90 | - | - | - | - |
| GMA3-*orf87* | complement(62260..64299) | 680 | vWFA [*Rhodococcus* phage ReqiDocB7] | 36 | 5e-42 | von Willebrand factor (pfam13519) |
| GMA3-*orf88* | complement(64299..65969) | 557 | ATPase family protein [*Gordonia* phage GTE7] | 46 | 2e-121 | AAA protein (pfam07728) |
| GMA3-*orf89* | complement(66035..66172) | 46 | - | - | - | - |
| GMA3-*orf90* | complement(66406..66882) | 159 | PREDICTED: RNA-binding motif protein, X-linked-like-2 [*Pan troglodytes*] | 39 | 2e-04 | - |
| GMA3-*orf91* | complement(67333..68085) | 251 | - | - | - | - |
| GMA3-*orf92* | complement(68228..68671) | 148 | gp091 [*Rhodococcus* phage ReqiDocB7] | 35 | 4e-11 | - |
| GMA3-*orf93* | complement(68717..69091) | 125 | gp162 [*Mycobacterium* phage Wildcat] | 46 | 8e-12 | - |
| GMA3-*orf94* | complement(69177..69422) | 82 | - | - | - | - |
| GMA3-*orf95* | complement(69410..69646) | 79 | - | - | - | - |
| GMA3-*orf96* | complement(69678..70583) | 302 | - | - | - | - |
| GMA3-*orf97* | complement(70650..70766) | 39 | - | - | - | - |
| GMA3-*orf98* | complement(71182..71544) | 121 | hypothetical protein A306_06092 [*Columba livia*] | 58 | 9e-13 | - |
| GMA3-*orf99* | complement(72890..73144) | 85 | - | - | - | - |
| GMA3-*orf100* | complement(73141..75102) | 654 | gp102 [*Rhodococcus* phage ReqiDocB7] | 29 | 2e-44 | Primase (TIGR01391) |
| GMA3-*orf101* | complement(75099..75464) | 122 | hypothetical protein COCSUDRAFT_57208 [*Coccomyxa subellipsoidea* C-169] | 35 | 4e-09 | - |
| GMA3-*orf102* | complement(75728..76318) | 197 | - | - | - | - |
| GMA3-*orf103* | complement(76315..77079) | 225 | gp105 [*Rhodococcus* phage ReqiDocB7] | 30 | 6e-23 | - |
| GMA3-*orf104* | complement(77179..77301) | 41 | - | - | - | - |
| GMA4-*orf1* | 79..492 | 138 | hypothetical protein LIKA_5 [*Streptomyces* phage Lika] | 60 | 2e-44 | Putative small terminase subunit |
| GMA4-*orf2* | 489..2087 | 533 | terminase [*Streptomyces auratus*] | 56 | 0.0 | Putative large terminase subunit |
| GMA4-*orf3* | 2101..3495 | 465 | hypothetical protein [*Gordonia malaquae*] | 97 | 0.0 | Portal protein (pfam05133) |
| GMA4-*orf4* | 3492..5039 | 516 | hypothetical protein [*Gordonia malaquae*] | 99 | 0.0 | Capsid maturation protease (PRK14694) |
| GMA4-*orf5* | 5343..5924 | 194 | hypothetical protein [*Gordonia malaquae*] | 94 | 7e-113 | Unknown (pfam14265) |
| GMA4-*orf6* | 5937..6311 | 125 | hypothetical protein [*Gordonia malaquae*] | 98 | 8e-73 | - |
| GMA4-*orf7* | 6326..7234 | 303 | hypothetical protein [*Gordonia malaquae*] | 93 | 0.0 | Phage structural protein |
| GMA4-*orf8* | 7238..7465 | 76 | hypothetical protein [*Gordonia malaquae*] | 92 | 1e-31 | - |
| GMA4-*orf9* | 7458..7847 | 130 | hypothetical protein [*Gordonia malaquae*] | 96 | 2e-81 | Phage protein (pfam09355) |
| GMA4-*orf10* | 7847..8170 | 108 | hypothetical protein [*Gordonia malaquae*] | 99 | 1e-68 | - |
| GMA4-*orf11* | 8224..8490 | 89 | hypothetical protein [*Gordonia malaquae*] | 76 | 6e-33 | - |
| GMA4-*orf12* | 8487..8873 | 129 | hypothetical protein [*Gordonia malaquae*] | 98 | 3e-83 | - |
| GMA4-*orf13* | 8955..9626 | 224 | hypothetical protein [*Gordonia malaquae*] | 92 | 1e-142 | Putative major tail structural protein |
| GMA4-*orf14* | 9946..10263 | 106 | hypothetical protein [*Gordonia malaquae*] | 99 | 3e-67 | Putative tail assembly protein |
| GMA4-*orf15* | 10245..10748 | 168 | hypothetical protein [*Gordonia malaquae*] | 98 | 1e-89 | Putative tail assembly protein translated by conserved programmed translational frameshift |
| GMA4-*orf16* | 10768..16050 | 1761 | hypothetical protein [*Gordonia malaquae*] | 97 | 0.0 | Tape measure protein (pfam05701; COG5412; pfam01464) |
| GMA4-*orf17* | 16043..16957 | 305 | hypothetical protein [*Gordonia malaquae*] | 99 | 0.0 | - |
| GMA4-*orf18* | 16957..18117 | 387 | hypothetical protein [*Gordonia malaquae*] | 98 | 0.0 | Unknown(pfam14594) |
| GMA4-*orf19* | 18117..19190 | 358 | hypothetical protein [*Gordonia malaquae*] | 97 | 0.0 | - |
| GMA4-*orf20* | 19192..20256 | 355 | hypothetical protein [*Gordonia malaquae*] | 96 | 0.0 | - |
| GMA4-*orf21* | 20328..21401 | 358 | hypothetical protein [*Gordonia rubripertincta*] | 58 | 1e-120 | Lysin (pfam01510; pfam08310) |
| GMA4-*orf22* | 21398..21685 | 96 | hypothetical protein [*Gordonia malaquae*] | 93 | 2e-56 | Glutaredoxin (pfam00462) |
| GMA4-*orf23* | 21682..21882 | 67 | DNA polymerase I [*Leifsonia xyli*] | 46 | 8e-07 | - |
| GMA4-*orf24* | 21879..22325 | 149 | hypothetical protein [*Gordonia malaquae*] | 95 | 1e-91 | - |
| GMA4-*orf25* | complement(22399..22749) | 117 | hypothetical protein [*Gordonia malaquae*] | 84 | 1e-46 | - |
| GMA4-*orf26* | 22913..23575 | 221 | hypothetical protein [*Mycobacterium colombiense*] | 37 | 7e-06 | Unknown (pfam05305) |
| GMA4-*orf27* | 23590..23799 | 70 | - | - | - | - |
| GMA4-*orf28* | complement(23993..24892) | 300 | hypothetical protein [*Rhodococcus pyridinivorans*] | 38 | 4e-33 | - |
| GMA4-*orf29* | complement(25217..26449) | 411 | phage integrase family protein [*Rhodococcus pyridinivorans*] | 43 | 8e-82 | Integrase (pfam00589) |
| GMA4-*orf30* | complement(26442..26648) | 69 | hypothetical protein [*Gordonia malaquae*] | 74 | 8e-28 | Unknown (pfam11662) |
| GMA4-*orf31* | complement(26645..27079) | 145 | unnamed protein product [*Rhodococcus* phage REQ2] | 64 | 3e-54 | Unknown (pfam06114) |
| GMA4-*orf32* | complement(27088..27585) | 166 | ribosomal protein S13 [*Corynebacterium falsenii* DSM 44353] | 37 | 1e-20 | Ribosomal protein S13 |
| GMA4-*orf33* | 27730..27987 | 86 | hypothetical protein [*Corynebacterium ulcerans*] | 45 | 7e-09 | HTH DNA Binding (pfam01381) |
| GMA4-*orf34* | 28046..28852 | 269 | hypothetical protein [*Mycobacterium abscessus*] | 64 | 4e-59 | Anti-repressor/Rha regulatory protein (pfam03374; pfam09669) |
| GMA4-*orf35* | 28849..29106 | 86 | - | - | - | - |
| GMA4-*orf36* | 29103..29303 | 67 | DNA-binding protein [*Streptomyces sclerotialus*] | 41 | 4e-08 | HTH DNA Binding (pfam12728) |
| GMA4-*orf37* | 29315..29482 | 56 | - | - | - | - |
| GMA4-*orf38* | 29479..29760 | 94 | hypothetical protein [*Mycobacterium avium*] | 39 | 8e-12 | - |
| GMA4-*orf39* | 29757..30101 | 115 | gp54 [*Mycobacterium* phage Charlie] | 38 | 4e-13 | - |
| GMA4-*orf40* | 30065..30856 | 264 | hypothetical protein [*Rhodococcus opacus*] | 42 | 2e-55 | - |
| GMA4-*orf41* | 30857..31489 | 211 | hypothetical protein [*Rhodococcus* sp. 29MFTsu3.1] | 33 | 3e-11 | - |
| GMA4-*orf42* | 31658..32065 | 136 | gp58 [*Mycobacterium* phage Dori] | 52 | 2e-39 | - |
| GMA4-*orf43* | 32065..32415 | 117 | gp82 [*Mycobacterium* phage Bxb1] | 38 | 5e-12 | - |
| GMA4-*orf44* | 32408..32542 | 45 | - | - | - | - |
| GMA4-*orf45* | 32539..32883 | 115 | - | - | - | - |
| GMA4-*orf46* | 32880..33362 | 161 | hypothetical protein [*Gordonia malaquae*] | 98 | 1e-35 | - |
| GMA4-*orf47* | 33410..33589 | 60 | - | - | - | - |
| GMA4-*orf48* | 33582..33890 | 103 | hypothetical protein [*Gordonia malaquae*] | 95 | 7e-46 | - |
| GMA4-*orf49* | 33848..34048 | 67 | MULTISPECIES: hypothetical protein [*Streptomyces*] | 55 | 6e-05 | - |
| GMA4-*orf50* | 34041..34310 | 90 | hypothetical protein [*Gordonia alkanivorans*] | 40 | 7e-09 | - |
| GMA4-*orf51* | complement(34307..34519) | 71 | hypothetical protein [*Gordonia malaquae*] | 97 | 5e-41 | - |
| GMA4-*orf52* | 34584..34763 | 60 | hypothetical protein EN35_20025 [*Rhodococcus qingshengii*] | 34 | 1e-04 | - |
| GMA4-*orf53* | 34736..34858 | 41 | - | - | - | - |
| GMA4-*orf54* | 34872..35675 | 268 | DNA methylase N-4 [*Corynebacterium aurimucosum*] | 73 | 2e-138 | DNA Methylase (pfam01555) |
| GMA4-*orf55* | 35668..35970 | 101 | hypothetical protein [*Gordonia malaquae*] | 100 | 3e-56 | - |
| GMA4-*orf56* | 35967..36167 | 67 | hypothetical protein [*Gordonia malaquae*] | 100 | 6e-39 | DNA binding (cd00569) |
| GMA4-*orf57* | 36164..36322 | 53 | - | - | - | - |
| GMA4-*orf58* | 36626..39130 | 835 | hypothetical protein [*Mycobacterium avium*] | 47 | 0.0 | Primase (pfam08706) |
| GMA4-*orf59* | 39801..40205 | 135 | - | - | - | - |
| GMA4-*orf60* | 40216..40572 | 119 | hypothetical protein [*Rhodococcus equi*] | 53 | 6e-27 | - |
| GMA4-*orf61* | 40576..40734 | 53 | - | - | - | - |
| GMA4-*orf62* | 40731..41486 | 252 | hypothetical protein [*Rhodococcus* sp. UNC363MFTsu5.1] | 42 | 6e-51 | - |
| GMA4-*orf63* | 41745..41891 | 49 | hypothetical protein [*Gordonia malaquae*] | 55 | 8e-08 | - |
| GMA4-*orf64* | 42024..42347 | 108 | hypothetical protein [*Nocardia farcinica*] | 33 | 5e-06 | - |
| GMA4-*orf65* | 42338..42712 | 125 | - | - | - | - |
| GMA4-*orf66* | 42724..43833 | 370 | hypothetical protein [*Aeromicrobium marinum*] | 47 | 1e-59 | Phage related tail structural protein (COG5310) |
| GMA4-*orf67* | 43834..4449043840..44490 | 219217 | HNH homing endonuclease domain protein [*Mycobacterium* phage Hamulus] | 445 | 4e-37 | Endonuclease (pfam13392; pfam07463) |
| GMA4-*orf68* | 44497..45141 | 215 | hypothetical protein [*Amycolatopsis taiwanensis*] | 29 | 2e-06 | - |
| GMA5-*orf1* | 93..500 | 136 | hypothetical protein [*Gordonia neofelifaecis*] | 72 | 9e-43 | Small terminase subunit |
| GMA5-*orf2* | 466..1860 | 465 | putative phage terminase protein [*Gordonia neofelifaecis*] | 68 | 0.0 | Large terminase subunit (pfam03354) |
| GMA5-*orf3* | 1869..2066 | 66 | hypothetical protein [*Gordonia neofelifaecis*] | 52 | 3e-09 | - |
| GMA5-*orf4* | 2108..3199 | 364 | hypothetical protein [*Gordonia neofelifaecis*] | 68 | 5e-158 | Portal protein (pfam04860) |
| GMA5-*orf5* | 3196..3984 | 263 | hypothetical protein [*Gordonia soli*] | 51 | 7e-67 | Lysin - D-alanyl-D-alanine carboxypeptidase (pfam13539) |
| GMA5-*orf6* | 4085..6046 | 654 | hypothetical protein [*Gordonia neofelifaecis*] | 63 | 0.0 | - |
| GMA5-*orf7* | 6050..6391 | 114 | unnamed protein product [*Rhodococcus* phage RRH1] | 46 | 3e-19 | - |
| GMA5-*orf8* | 6391..6732 | 114 | hypothetical protein [*Gordonia neofelifaecis*] | 63 | 2e-35 | - |
| GMA5-*orf9* | 6746..7207 | 154 | hypothetical protein [*Gordonia neofelifaecis*] | 76 | 3e-77 | - |
| GMA5-*orf10* | 7204..7551 | 116 | hypothetical protein [*Gordonia neofelifaecis*] | 47 | 4e-22 | Phage protein HK97/gp10 family- possibly tail morphogenesis (TIGR01725) |
| GMA5-*orf11* | 7567..7860 | 98 | hypothetical protein [*Gordonia neofelifaecis*] | 60 | 7e-30 | - |
| GMA5-*orf12* | 7975..10002 | 676 | TP901 family phage tail tape measure protein , putative [*Gordonia neofelifaecis*] | 54 | 1e-176 | Tape measure protein (COG5412) |
| GMA5-*orf13* | 9999..11417 | 473 | unnamed protein product [*Rhodococcus* phage RRH1] | 25 | 2e-23 | - |
| GMA5-*orf14* | 11420..12025 | 202 | unnamed protein product [*Rhodococcus* phage RRH1] | 30 | 1e-15 | Phage structural protein |
| GMA5-*orf15* | 12085..12933 | 283 | bacteriophage protein [*Mycobacterium thermoresistibile*] | 41 | 2e-59 | Phage structural protein (pfam08237) |
| GMA5-*orf16* | complement(13003..13107) | 35 | - | - | - | - |
| GMA5-*orf17* | complement(13184..13993) | 270 | integrase [*Gordonia neofelifaecis*] | 69 | 2e-122 | Integrase (pfam00589) |
| GMA5-*orf18* | complement(14074..14349) | 92 | hypothetical protein [*Salinispora arenicola*] | 62 | 5e-24 | - |
| GMA5-*orf19* | complement(14346..14633) | 96 | putative DNA-binding protein [*Gordonia neofelifaecis*] | 49 | 2e-16 | HTH DNA binding domain (pfam12844) |
| GMA5-*orf20* | 14712..14891 | 60 | hypothetical protein [*Tomitella biformata*] | 51 | 1e-07 | HTH DNA binding domain (pfam12728) |
| GMA5-*orf21* | 14885..15145 | 87 | hypothetical protein [*Gordonia neofelifaecis*] | 43 | 2e-11 | - |
| GMA5-*orf22* | 15142..15327 | 62 | hypothetical protein [*Gordonia neofelifaecis*] | 49 | 2e-06 | - |
| GMA5-*orf23* | 15324..15518 | 65 | hypothetical protein [*Gordonia malaquae*] | 69 | 2e-20 | Hin/HTH DNA binding domain (cd00569) |
| GMA5-*orf24* | 15607..15828 | 74 | - | - | - | - |
| GMA5-*orf25* | 15816..16079 | 88 | - | - | - | - |
| GMA5-*orf26* | 16193..17065 | 291 | DNA polymerase III subunit epsilon [*Gordonia neofelifaecis*] | 53 | 1e-51 | DNA polymerase III subunit epsilon (COG0847) |
| GMA5-*orf27* | 17062..17340 | 93 | unnamed protein product [*Rhodococcus* phage RRH1] | 62 | 8e-31 | HNH endonuclease (pfam01844) |
| GMA5-*orf28* | 17424..17558 | 45 | - | - | - | - |
| GMA6-*orf1* | 21..206 | 62 | - | - | - | - |
| GMA6-*orf2* | 203..373 | 57 | - | - | - | Putative small terminase subunit |
| GMA6-*orf3* | 370..2850 | 827 | large terminase subunit [*Methanobacterium* phage psiM2] | 38 | 2e-54 | Large terminase subunit (pfam03237; PRK14715; smart00306) |
| GMA6-*orf4* | 2942..3256 | 105 | - | - | - | - |
| GMA6-*orf5* | 3281..3460 | 60 | - | - | - | - |
| GMA6-*orf6* | 3457..3711 | 85 | - | - | - | - |
| GMA6-*orf7* | 3711..4031 | 107 | - | - | - | - |
| GMA6-*orf8* | 4028..4612 | 195 | gp40 [*Mycobacterium* phage Che12] | 47 | 2e-24 | Nucleoside Triphosphate Pyrophosphohydrolase (cd11541) |
| GMA6-*orf9* | 4609..4851 | 81 | - | - | - | - |
| GMA6-*orf10* | 4844..5236 | 131 | - | - | - | - |
| GMA6-*orf11* | 5324..7891 | 856 | hypothetical protein [*Streptomyces* sp. Amel2xE9] | 42 | 3e-79 | Portal protein (pfam04860; pfam04233) |
| GMA6-*orf12* | 7888..8862 | 325 | hypothetical protein [*Meiothermus chliarophilus*] | 31 | 1e-16 | RNA ligase (pfam13563) |
| GMA6-*orf13* | 8862..9977 | 372 | - | - | - | VIP2; A family of actin-ADP-ribosylating toxin (cd00233) |
| GMA6-*orf14* | 9974..13003 | 1010 | hypothetical protein [*Tomitella biformata*] | 44 | 1e-08 | HNH endonuclease (pfam01844) |
| GMA6-*orf15* | 13000..13599 | 200 | hypothetical protein [*Rhodococcus fascians*] | 56 | 1e-11 | - |
| GMA6-*orf16* | 13599..14291 | 231 | hypothetical protein [*Gordonia soli*] | 37 | 1e-28 | - |
| GMA6-*orf17* | 14291..14863 | 191 | hypothetical protein [*Segniliparus rugosus*] | 48 | 9e-12 | - |
| GMA6-*orf18* | 14860..15507 | 216 | hypothetical protein [*Gordonia neofelifaecis*] | 55 | 5e-37 | - |
| GMA6-*orf19* | 15585..17039 | 485 | hypothetical protein [*Streptomyces rimosus*] | 32 | 3e-35 | Prohead protease (pfam04586) |
| GMA6-*orf20* | 17179..18546 | 456 | capsid protein [*Streptomyces* sp. PRh5] | 36 | 9e-73 | Phage capsid structural protein (pfam05065) |
| GMA6-*orf21* | 18597..18785 | 63 | - | - | - | - |
| GMA6-*orf22* | 18785..19093 | 103 | - | - | - | - |
| GMA6-*orf23* | 19099..19305 | 69 | - | - | - | - |
| GMA6-*orf24* | 19372..19947 | 192 | - | - | - | - |
| GMA6-*orf25* | 19951..20526 | 192 | - | - | - | - |
| GMA6-*orf26* | 20625..20996 | 124 | phage protein, HK97 gp10 family [*Sideroxydans lithotrophicus*] | 37 | 3e-05 | Virion morphogenesis protein (pfam05069) |
| GMA6-*orf27* | 20993..21688 | 232 | - | - | - | - |
| GMA6-*orf28* | 21703..22032 | 110 | - | - | - | - |
| GMA6-*orf29* | 22036..23484 | 483 | hypothetical protein [*Kribbella catacumbae*] | 43 | 4e-50 | Phage tail sheath structural protein (pfam04984) |
| GMA6-*orf30* | 23527..23934 | 136 | phage tail protein [*Algoriphagus marincola*] | 15 | 5e-38 | Putative major tail protein (pfam06841) |
| GMA6-*orf31* | 24035..24604 | 190 | hypothetical protein [*Kribbella catacumbae*] | 26 | 6e-05 | Putative tail assembly protein |
| GMA6-*orf32* | 24586..24801 | 72 | - | - | - | Putative tail assembly protein translated by conserved programmed translational frameshift |
| GMA6-*orf33* | 24842..28261 | 1140 | peptidase M23 [*Staphylococcus* sp. URHA0057] | 26 | 3e-29 | Tape measure protein (COG5412) |
| GMA6-*orf34* | 28264..29061 | 266 | hypothetical protein [*Kribbella catacumbae*] | 23 | 1e-04 | Lysin (pfam01476) |
| GMA6-*orf35* | 29073..29516 | 148 | hypothetical protein [*Streptomyces albus*] | 41 | 2e-20 | Endonuclease (pfam13392) |
| GMA6-*orf36* | 29518..30879 | 454 | unnamed protein product [*Rhodococcus* phage REQ2] | 63 | 9e-49 | - |
| GMA6-*orf37* | 30895..32241 | 449 | hydrolase Nlp/P60 [*Gordonia rhizosphera*] | 51 | 2e-29 | Cell wall hydrolase (COG0791) |
| GMA6-*orf38* | 32254..32694 | 147 | - | - | - | - |
| GMA6-*orf39* | 32691..33092 | 134 | - | - | - | - |
| GMA6-*orf40* | 33089..33412 | 108 | hypothetical protein [*Kribbella catacumbae*] | 33 | 6e-09 | Lysozyme (pfam04965) |
| GMA6-*orf41* | 33414..34571 | 386 | hypothetical protein [*Kribbella catacumbae*] | 39 | 3e-68 | Unknown (COG3299) |
| GMA6-*orf42* | 34564..35736 | 391 | hypothetical protein [*Kribbella catacumbae*] | 29 | 1e-19 | - |
| GMA6-*orf43* | 35754..37736 | 661 | hypothetical protein [*Kribbella catacumbae*] | 32 | 7e-04 | Phage structural protein |
| GMA6-*orf44* | 37736..38137 | 134 | - | - | - | - |
| GMA6-*orf45* | 38130..41393 | 1088 | hypothetical protein [*Mycobacterium* sp. URHD0025] | 57 | 1e-120 | Lysin - Peptidase (cd06418; pfam01510; pfam01551; pfam13810) |
| GMA6-*orf46* | 41390..41899 | 170 | hypothetical protein [*Corynebacterium argentoratense*] | 38 | 1e-06 | Putative holin |
| GMA6-*orf47* | 41896..42207 | 104 | - | - | - | - |
| GMA6-*orf48* | 42191..42718 | 176 | membrane protein [*Rhodococcus* sp. JVH1] | 35 | 9e-06 | - |
| GMA6-*orf49* | complement(42746..42877) | 44 | - | - | - | - |
| GMA6-*orf50* | complement(43215..43670) | 152 | unnamed protein product [*Synechococcus* phage S-CBS2] | 37 | 8e-19 | Recombination endonuclease (pfam02945) |
| GMA6-*orf51* | complement(43667..44602) | 312 | helicase DnaB [*Caldicellulosiruptor kronotskyensis*] | 27 | 1e-23 | DNA Primase (COG0358) |
| GMA6-*orf52* | complement(44611..46011) | 467 | DNA helicase [*Acidothermus cellulolyticus*] | 34 | 6e-28 | Replicative helicase (COG03050) |
| GMA6-*orf53* | complement(46243..46431) | 63 | - | - | - | - |
| GMA6-*orf54* | complement(46511..46978) | 156 | - | - | - | - |
| GMA6-*orf55* | 48033..48371 | 113 | - | - | - | - |
| GMA6-*orf56* | 48391..48603 | 71 | - | - | - | - |
| GMA6-*orf57* | 48596..48976 | 127 | hypothetical protein [*Mycobacterium genavense*] | 31 | 2e-06 | Unknown (pfam05305) |
| GMA6-*orf58* | 48979..49254 | 92 | - | - | - | - |
| GMA6-*orf59* | 49254..49418 | 55 | - | - | - | - |
| GMA6-*orf60* | 49469..49927 | 153 | - | - | - | - |
| GMA6-*orf61* | 49979..51178 | 400 | ATPase AAA [*Thioalkalivibrio thiocyanodenitrificans*] | 33 | 5e-22 | AAA protein (pfam07728) |
| GMA6-*orf62* | 51221..53134 | 638 | von Willebrand factor A [*Pelobacter propionicus*] | 21 | 7e-04 | Von Willlebrand factor/Cobalmin biosybthesis (pfam06213;pfam13519) |
| GMA6-*orf63* | 53274..53819 | 182 | - | - | - | - |
| GMA6-*orf64* | 53816..54250 | 145 | - | - | - | - |
| GMA6-*orf65* | 54263..54556 | 98 | hypothetical protein CRB1_33 [*Mycobacterium* phage CRB1] | 42 | 4e-08 | - |
| GMA6-*orf66* | 54553..54771 | 73 | hypothetical protein PBI_RHYNO_66 [*Mycobacterium* phage RhynO] | 45 | 9e-06 | - |
| GMA6-*orf67* | 55029..55838 | 270 | - | - | - | - |
| GMA6-*orf68* | 55843..56382 | 180 | - | - | - | - |
| GMA6-*orf69* | 56385..56627 | 81 | - | - | - | HTH DNA binding (pfam13411) |
| GMA6-*orf70* | 56628..56978 | 117 | - | - | - | - |
| GMA6-*orf71* | 57069..57743 | 225 | - | - | - | - |
| GMA6-*orf72* | 57807..58559 | 251 | - | - | - | - |
| GMA6-*orf73* | 58715..58966 | 84 | - | - | - | - |
| GMA6-*orf74* | 59155..59451 | 99 | - | - | - | - |
| GMA6-*orf75* | 59453..59935 | 161 | - | - | - | - |
| GMA6-*orf76* | 59922..60326 | 135 | - | - | - | - |
| GMA6-*orf77* | 60323..60514 | 64 | - | - | - | - |
| GMA6-*orf78* | 60511..60807 | 99 | - | - | - | - |
| GMA6-*orf79* | 60811..61245 |  | hypothetical protein [Streptomyces albus] | 41 | 1e-20 | Endonuclease (pfam13392) |
| GMA6-*orf80* | 61160..64054 | 965 | DNA polymerase III alpha subunit [*Halanaerobium saccharolyticum*] | 32 | 1e-117 | DNA polymerase III (COG0587) |
| GMA6-*orf81* | 64168..64497 | 110 | HNH domain protein [*Mycobacterium* phage Goku] | 49 | 6e-23 | Endonuclease (pfam13392) |
| GMA6-*orf82* | 64501..64749 | 83 | - | - | - | - |
| GMA6-*orf83* | 64766..64912 | 49 | - | - | - | - |
| GMA6-*orf84* | 64909..65130 | 74 | - | - | - | - |
| GMA6-*orf85* | 65127..65333 | 69 | - | - | - | - |
| GMA6-*orf86* | 65333..65557 | 75 | - | - | - | - |
| GMA6-*orf87* | 65491..65952 | 154 | - | - | - | - |
| GMA6-*orf88* | 65953..67116 | 388 | recombinase RecA [*Hirschia maritima*] | 38 | 5e-60 | Recombinase (pfam00154) |
| GMA6-*orf89* | 67162..67716 | 185 | hypothetical protein [Sphingobium chungbukense] | 44 | 6e-23 | Endonuclease (pfam13392) |
| GMA6-*orf90* | 67738..67881 | 48 | - | - | - | - |
| GMA6-*orf91* | 67874..68185 | 104 | - | - | - | - |
| GMA6-*orf92* | 68182..68454 | 91 | - | - | - | - |
| GMA6-*orf93* | 68445..69272 | 276 | hypothetical protein TCA2_4616 [*Paenibacillus* sp. TCA20] | 29 | 9e-08 | - |
| GMA6-*orf94* | 69278..69442 | 55 | - | - | - | - |
| GMA6-*orf95* | 69442..69879 | 146 | MedDCM-OCT-S33-C31-cds10 [*Candidatus* Actinomarina minuta] | 42 | 4e-17 | - |
| GMA6-*orf96* | 70127..70558 | 144 | - | - | - | - |
| GMA6-*orf97* | 70563..71153 | 197 | - | - | - | Holliday junction resolvase (PRK00039) |
| GMA6-*orf98* | 71158..72720 | 521 | hypothetical protein [*Microbacterium* sp. UCD-TDU] | 47 | 2e-34 | Nuclease (pfam02195; pfam14386) |
| GMA6-*orf99* | 72859..73206 | 116 | - | - | - | valyl-tRNA synthetase (PRK14900) |
| GMA6-*orf100* | 73320..73802 | 161 | - | - | - | Metalloenzyme protein (cd08070) |
| GMA6-*orf101* | 73830..74732 | 301 | hypothetical protein [*Streptomyces sulphureus*] | 38 | 1e-35 | - |
| GMA6-*orf102* | 74814..75557 | 248 | - | - | - | - |
| GMA6-*orf103* | 75571..76686 | 372 | hypothetical protein [*Aeromicrobium marinum*] | 42 | 8e-41 | Phage-related tail fibre protein (COG5301) |
| GMA6-*orf104* | 76794..78356 | 521 | - | - | - | - |
| GMA6-*orf105* | 78755..78997 | 81 | - | - | - | - |
| GMA6-*orf106* | 79073..79291 | 73 | - | - | - | - |
| GMA6-*orf107* | 79682..80032 | 117 | - | - | - | - |
| GMA6-*orf108* | 80146..81057 | 304 | - | - | - | - |
| GMA6-*orf109* | 81475..81708 | 78 | - | - | - | - |
| GMA6-*orf110* | 81714..81839 | 42 | - | - | - | - |
| GMA6-*orf111* | 81832..82194 | 121 | gp58 [*Mycobacterium* phage Pipefish] | 54 | 3e-28 | - |
| GMA6-*orf112* | 82328..82540 | 71 | - | - | - | - |
| GMA6-*orf113* | 82537..82827 | 97 | putative regulator [*Tsukamurella* phage TPA2] | 61 | 1e-06 | - |
| GMA6-*orf114* | 82827..82988 | 54 | - | - | - | - |
| GMA6-*orf115* | 83005..83148 | 48 | - | - | - | - |
| GMA7-*orf1* | 42..1499 | 486 | putative small terminase [*Gordonia* phage GTE7] | 99 | 0.0 | Putative smalll terminase subunit |
| GMA7-*orf2* | 1492..3459 | 656 | terminase large subunit [*Gordonia* phage GTE7] | 100 | 0.0 | Putative large terminase subunit |
| GMA7-*orf3* | 3574..5289 | 572 | hypothetical protein GTE7_gp003 [*Gordonia* phage GTE7] | 99 | 0.0 | Unknown (pfam06074) |
| GMA7-*orf4* | 5276..6424 | 383 | hypothetical protein GTE7_gp004 [*Gordonia* phage GTE7] | 63 | 6e-157 | YadA-like, left handed beta roll protein (cd12820) |
| GMA7-*orf5* | 6434..6661 | 76 | hypothetical protein GTE7_gp005 [*Gordonia* phage GTE7] | 48 | 9.e-09 | - |
| GMA7-*orf6* | 6743..6994 | 84 | hypothetical protein GTE7_gp006 [*Gordonia* phage GTE7] | 99 | 5e-55 | - |
| GMA7-*orf7* | 7009..8187 | 393 | hypothetical protein GTE7_gp007 [*Gordonia* phage GTE7] | 99 | 0.0 | - |
| GMA7-*orf8* | 8221..8700 | 160 | hypothetical protein GTE7_gp008 [*Gordonia* phage GTE7] | 98 | 3e-110 | - |
| GMA7-*orf9* | 8712..9914 | 401 | putative major capsid protein [*Gordonia* phage GTE7] | 99 | 0.0 | Phage major capsid protein (pfam03864) |
| GMA7-*orf10* | 9931..10125 | 65 | hypothetical protein GTE7_gp010 [*Gordonia* phage GTE7] | 100 | 6e-36 | - |
| GMA7-*orf11* | 10200..10454 | 85 | hypothetical protein GTE7_gp011 [*Gordonia* phage GTE7] | 96 | 7e-35 | - |
| GMA7-*orf12* | 10464..10979 | 172 | hypothetical protein GTE7_gp012 [*Gordonia* phage GTE7] | 100 | 2e-121 | - |
| GMA7-*orf13* | 11072..11467 | 132 | hypothetical protein GTE7_gp013 [*Gordonia* phage GTE7] | 98 | 8e-86 | - |
| GMA7-*orf14* | 11464..11949 | 162 | hypothetical protein GTE7_gp014 [*Gordonia* phage GTE7] | 99 | 3e-113 | - |
| GMA7-*orf15* | 11962..12267 | 102 | hypothetical protein GTE7_gp015 [*Gordonia* phage GTE7] | 99 | 3e-63 | - |
| GMA7-*orf16* | 12271..13290 | 340 | hypothetical protein GTE7_gp016 [*Gordonia* phage GTE7] | 99 | 0.0 | Major tail protein |
| GMA7-*orf17* | 13487..14077 | 197 | putative tail assembly protein [*Gordonia* phage GTE7] | 98 | 2e-137 | Putative tail assembly protein |
| GMA7-*orf18* | 14059..14337 | 93 | hypothetical protein GTE7_gp018 [*Gordonia* phage GTE7] | 97 | 5e-39 | Putative tail assembly protein translated by conserved programmed translational frameshift |
| GMA7-*orf19* | 14475..23615 | 3047 | phage tape measure protein [*Gordonia* phage GTE7] | 97 | 0.0 | Tape measure protein (pfam10145; COG1196; COG5412; pfam01464) |
| GMA7-*orf20* | 23615..26158 | 848 | hypothetical protein GTE7_gp020 [*Gordonia* phage GTE7] | 99 | 0.0 | - |
| GMA7-*orf21* | 26160..27899 | 580 | hypothetical protein GTE7_gp021 [*Gordonia* phage GTE7] | 99 | 0.0 | Tail protein (pfam13550) |
| GMA7-*orf22* | 27899..30667 | 923 | hypothetical protein GTE7_gp022 [*Gordonia* phage GTE7] | 98 | 0.0 | - |
| GMA7-*orf23* | 30667..31671 | 335 | hypothetical protein GTE7_gp023 [*Gordonia* phage GTE7] | 96 | 0.0 | - |
| GMA7-*orf24* | 31671..31964 | 98 | hypothetical protein GTE7_gp024 [*Gordonia* phage GTE7] | 98 | 2e-61 | - |
| GMA7-*orf25* | 31966..32343 | 126 | hypothetical protein GTE7_gp025 [*Gordonia* phage GTE7] | 100 | 2e-81 | - |
| GMA7-*orf26* | 32429..32848 | 140 | hypothetical protein GTE7_gp026 [*Gordonia* phage GTE7] | 100 | 6e-95 | - |
| GMA7-*orf27* | 32848..33171 | 108 | hypothetical protein GTE7_gp027 [*Gordonia* phage GTE7] | 97 | 1e-69 | - |
| GMA7-*orf28* | 33168..33803 | 212 | lysozyme [*Gordonia* phage GTE7] | 99 | 3e-155 | Lysin (pfam01510) |
| GMA7-*orf29* | 33800..34627 | 276 | putative peptidase [*Gordonia* phage GTE7] | 99 | 0.0 | Lysin/Peptidase (pfam01551) |
| GMA7-*orf30* | 34824..35057 | 78 | - | - | - | Putative holin |
| GMA7-*orf31* | 35045..35515 | 157 | hypothetical protein GTE7_gp030 [*Gordonia* phage GTE7] | 98 | 5e-108 | - |
| GMA7-*orf32* | 35508..35840 | 111 | hypothetical protein GTE7_gp031 [*Gordonia* phage GTE7] | 97 | 1e-67 | - |
| GMA7-*orf33* | complement(35837..36241) | 135 | hypothetical protein GTE7_gp032 [*Gordonia* phage GTE7] | 99 | 2e-88 | - |
| GMA7-*orf34* | complement(36225..36341) | 39 | - | - | - | - |
| GMA7-*orf35* | complement(36338..37540) | 401 | hypothetical protein GTE7_gp033 [*Gordonia* phage GTE7] | 99 | 0.0 | Nuclease (pfam12705) |
| GMA7-*orf36* | complement(37757..38170) | 138 | hypothetical protein GTE7_gp034 [*Gordonia* phage GTE7] | 100 | 8e-84 | - |
| GMA7-*orf37* | complement(38252..38365) | 38 | hypothetical protein GTE7_gp035 [*Gordonia* phage GTE7] | 100 | 3e-16 | - |
| GMA7-*orf38* | complement(38788..39507) | 240 | DNA methylase [*Gordonia* phage GTE7] | 99 | 1e-174 | DNA methylase (pfam01555) |
| GMA7-*orf39* | complement(39504..39770) | 89 | hypothetical protein GTE7_gp038 [*Gordonia* phage GTE7] | 97 | 2e-48 | - |
| GMA7-*orf40* | complement(39767..40000) | 78 | hypothetical protein GTE7_gp039 [*Gordonia* phage GTE7] | 99 | - | - |
| GMA7-*orf41* | complement(39997..40896) | 300 | lysinB protein [*Gordonia* phage GTE7] | 100 | 0.0 | Lysin/cutinase (pfam01083) |
| GMA7-*orf42* | complement(40898..41383) | 162 | hypothetical protein GTE7_gp041 [*Gordonia* phage GTE7] | 99 | 4e-113 | Nucleoside Triphosphate Pyrophosphohydrolase (cd11542) |
| GMA7-*orf43* | complement(41380..41505) | 42 | hypothetical protein GTE7_gp042 [*Gordonia* phage GTE7] | 100 | 3e-20 | - |
| GMA7-*orf44* | complement(41535..42110) | 192 | hypothetical protein GTE7_gp043 [*Gordonia* phage GTE7] | 100 | 4e-140 | - |
| GMA7-*orf45* | complement(42103..42255) | 51 | hypothetical protein GTE7_gp044 [*Gordonia* phage GTE7] | 98 | 3e-26 | - |
| GMA7-*orf46* | complement(42269..42634) | 122 | hypothetical protein GTE7_gp045 [*Gordonia* phage GTE7] | 99 | 9e-82 | Unknown (pfam14359) |
| GMA7-*orf47* | complement(42624..43763) | 380 | DNA polymerase III beta subunit [*Gordonia* phage G TE7] | 99 | 0.0 | DNA poymearse III beta clamp (COG0592) |
| GMA7-*orf48* | complement(43964..44584) | 207 | exonuclease [*Gordonia* phage GTE7] | 100 | 6e-149 | Exonuclease (pfam00929) |
| GMA7-*orf49* | complement(44581..44766) | 62 | DNA binding protein [*Gordonia* phage GTE7] | 98 | 3e-35 | HTH DNA binding (pfam12728) |
| GMA7-*orf50* | complement(45045..45296) | 84 | hypothetical protein GTE7_gp049 [*Gordonia* phage GTE7] | 100 | 4e-52 | - |
| GMA7-*orf51* | complement(45296..46927) | 544 | helicase [*Gordonia* phage GTE7] | 99 | 0.0 | Helicase (COG0553) |
| GMA7-*orf52* | complement(46931..47533) | 201 | hypothetical protein GTE7_gp051 [*Gordonia* phage GTE7] | 92 | 1e-130 | - |
| GMA7-*orf53* | complement(47654..48700) | 349 | hypothetical protein GTE7_gp052 [*Gordonia* phage GTE7] | 99 | 0.0 | - |
| GMA7-*orf54* | complement(48834..49136) | 101 | hypothetical protein GTE7_gp053 [*Gordonia* phage GTE7] | 100 | 2e-66 | - |
| GMA7-*orf55* | complement(49133..49471) | 113 | hypothetical protein GTE7_gp054 [*Gordonia* phage GTE7] | 100 | 9e-78 | - |
| GMA7-*orf56* | complement(49477..49665) | 63 | hypothetical protein GTE7_gp055 [*Gordonia* phage GTE7] | 97 | 3e-33 | - |
| GMA7-*orf57* | complement(49665..50288) | 208 | hypothetical protein GTE7_gp056 [*Gordonia* phage GTE7] | 99 | 1e-151 | - |
| GMA7-*orf58* | complement(50297..50746) | 150 | hypothetical protein GTE7_gp057 [*Gordonia* phage GTE7] | 96 | 1e-101 | - |
| GMA7-*orf59* | complement(50784..51029) | 82 | hypothetical protein GTE7_gp058 [*Gordonia* phage GTE7] | 100 | 8e-53 | - |
| GMA7-*orf60* | complement(51045..51902) | 286 | hypothetical protein GTE7_gp059 [*Gordonia* phage GTE7] | 88 | 0.0 | - |
| GMA7-*orf61* | complement(51895..52239) | 115 | hypothetical protein GTE7_gp060 [*Gordonia* phage GTE7] | 99 | 1e-73 | - |
| GMA7-*orf62* | complement(52243..52506) | 88 | hypothetical protein GTE7_gp061 [*Gordonia* phage GTE7] | 98 | 6e-53 | - |
| GMA7-*orf63* | complement(52668..53402) | 245 | hypothetical protein GTE7_gp062 [*Gordonia* phage GTE7] | 97 | 1e-177 | - |
| GMA7-*orf64* | complement(53399..53545) | 49 | hypothetical protein GTE7_gp063 [*Gordonia* phage GTE7] | 98 | 3e-22 | - |
| GMA7-*orf65* | complement(53545..53802) | 86 | hypothetical protein GTE7_gp064 [*Gordonia* phage GTE7] | 99 | 2e-55 | - |
| GMA7-*orf66* | complement(53977..54399) | 141 | hypothetical protein GTE7_gp065 [*Gordonia* phage GTE7] | 99 | 2e-94 | - |
| GMA7-*orf67* | complement(54513..55064) | 184 | hypothetical protein GTE7_gp066 [*Gordonia* phage GTE7] | 99 | 2e-131 | - |
| GMA7-*orf68* | complement(55074..55277) | 68 | hypothetical protein GTE7_gp067 [*Gordonia* phage GTE7] | 99 | 6e-39 | - |
| GMA7-*orf69* | complement(55489..55728) | 80 | hypothetical protein GTE7_gp068 [*Gordonia* phage GTE7] | 99 | 5e-50 | - |
| GMA7-*orf70* | complement(55725..55952) | 76 | hypothetical protein GTE7_gp069 [*Gordonia* phage GTE7] | 99 | 2e-44 | - |
| GMA7-*orf71* | complement(55949..57772) | 608 | hypothetical protein GTE7_gp071 [*Gordonia* phage GTE7] | 97 | 0.0 | Cobalmin biosynthesis von willebrand factor (pfam11775) |
| GMA7-*orf72* | complement(57807..59429) | 541 | ATPase family protein [*Gordonia* phage GTE7] | 98 | 0.0 | AAA protein (pfam07728) |
| GMA7-*orf73* | complement(59511..60140) | 210 | hypothetical protein GTE7_gp073 [*Gordonia* phage GTE7] | 98 | 6e-151 | - |
| GMA7-*orf74* | complement(60152..60526) | 125 | hypothetical protein GTE7_gp075 [*Gordonia* phage GTE7] | 100 | 8e-86 | - |
| GMA7-*orf75* | complement(60584..60712) | 43 | hypothetical protein GTE7_gp076 [*Gordonia* phage GTE7] | 95 | 1e-20 | - |
| GMA7-*orf76* | complement(60699..61589) | 297 | hypothetical protein GTE7_gp077 [*Gordonia* phage GTE7] | 97 | 0.0 | - |
| GMA7-*orf77* | complement(61570..61806) | 79 | hypothetical protein GTE7_gp078 [*Gordonia* phage GTE7] | 97 | 7e-48 | - |
| GMA7-*orf78* | complement(61803..61985) | 61 | hypothetical protein GTE7_gp079 [*Gordonia* phage GTE7] | 98 | 2e-33 | - |
| GMA7-*orf79* | complement(61982..62356) | 125 | hypothetical protein GTE7_gp080 [*Gordonia* phage GTE7] | 100 | 2e-85 | - |
| GMA7-*orf80* | complement(62617..62874) | 86 | hypothetical protein GTE7_gp083 [*Gordonia* phage GTE7] | 95 | 2e-51 | - |
| GMA7-*orf81* | complement(63157..64095) | 313 | hypothetical protein GTE7_gp084 [*Gordonia* phage GTE7] | 98 | 0.0 | - |
| GMA7-*orf82* | complement(64219..64500) | 94 | hypothetical protein GTE7_gp085 [*Gordonia* phage GTE7] | 94 | 2e-42 | - |
| GMA7-*orf83* | complement(64497..64619) | 41 | hypothetical protein GTE7_gp086 [*Gordonia* phage GTE7] | 98 | 7e-18 | - |
| GMA7-*orf84* | complement(64592..64843) | 84 | hypothetical protein GTE7_gp087 [*Gordonia* phage GTE7] | 100 | 4e-53 | - |
| GMA7-*orf85* | complement(64858..65061) | 68 | hypothetical protein GTE7_gp088 [*Gordonia* phage GTE7] | 100 | 4e-27 | - |
| GMA7-*orf86* | complement(65115..65279) | 55 | hypothetical protein GTE7_gp089 [*Gordonia* phage GTE7] | 53 | 1e-08 | - |
| GMA7-*orf87* | complement(65276..65578) | 101 | hypothetical protein GTE7_gp090 [*Gordonia* phage GTE7] | 40 | 4e-14 | - |
| GMA7-*orf88* | complement(65690..65980) | 97 | hypothetical protein GTE7_gp092 [*Gordonia* phage GTE7] | 34 | 2e-05 | - |
| GMA7-*orf89* | complement(66094..66327) | 78 | - | - | - | - |
| GMA7-*orf90* | complement(66414..66899) | 162 | hypothetical protein GTE7_gp091 [*Gordonia* phage GTE7] | 59 | 8e-48 | - |
| GMA7-*orf91* | complement(67218..67400) | 61 | - | - | - | - |
| GMA7-*orf92* | complement(67467..67901) | 145 | - | - | - | - |
| GMA7-*orf93* | complement(68439..68672) | 78 | hypothetical protein GTE7_gp095 [*Gordonia* phage GTE7] | 96 | 3e-44 | - |
| GMA7-*orf94* | complement(68674..69123) | 150 | hypothetical protein GTE7_gp096 [*Gordonia* phage GTE7] | 99 | 8e-100 | - |
| GMA7-*orf95* | complement(69120..69380) | 87 | hypothetical protein GTE7_gp097 [*Gordonia* phage GTE7] | 97 | 2e-53 | - |
| GMA7-*orf96* | complement(69377..71620) | 748 | putative primase [*Gordonia* phage GTE7] | 98 | 0.0 | Putative primase (pfam13148) |
| GMA7-*orf97* | complement(71617..71850) | 78 | hypothetical protein GTE7_gp099 [*Gordonia* phage GTE7] | 95 | 9e-46 | - |
| GMA7-*orf98* | complement(71825..71938) | 38 | hypothetical protein GTE7_gp100 [*Gordonia* phage GTE7] | 95 | 4.e-07 | - |
| GMA7-*orf99* | complement(71935..72309) | 125 | hypothetical protein GTE7_gp101 [*Gordonia* phage GTE7] | 99 | 7e-84 | - |
| GMA7-*orf100* | complement(72341..72520) | 60 | hypothetical protein GTE7_gp102 [*Gordonia* phage GTE7] | 100 | 2e-36 | - |
| GMA7-*orf101* | complement(72517..73353) | 279 | hypothetical protein GTE7_gp103 [*Gordonia* phage GTE7] | 97 | 0.0 | Unknown (COG4951) |
| GTE6-*orf1* | 65..625 | 187 | - | - | - | Putative small terminase subunit |
| GTE6-*orf2* | 622..2220 | 533 | hypothetical protein [*Streptomyces sulphureus*] | 40 | 5e-108 | Large terminase subunit (pfam03237) |
| GTE6-*orf3* | 2277..2726 | 150 | - | - | - | - |
| GTE6-*orf4* | 2723..2923 | 67 | - | - | - | - |
| GTE6-*orf5* | 2923..3546 | 208 | - | - | - | - |
| GTE6-*orf6* | 3543..3758 | 72 | - | - | - | - |
| GTE6-*orf7* | 3774..3965 | 64 | - | - | - | - |
| GTE6-*orf8* | 3969..4424 | 152 | - | - | - | - |
| GTE6-*orf9* | 4474..4629 | 52 | - | - | - | - |
| GTE6-*orf10* | 4626..4880 | 85 | unnamed protein product [*Gordonia* phage GTE5] | 52 | 2e-08 | - |
| GTE6-*orf11* | 4914..5237 | 108 | - | - | - | - |
| GTE6-*orf12* | 5362..5802 | 147 | membrane protein [*Rhodococcus equi* 103S] | 70 | 1e-38 | Host cell surface-exposed lipoprotein (pfam07553) |
| GTE6-*orf13* | 5949..6287 | 113 | hypothetical protein [*Streptomyces violaceusniger*] | 37 | 2e-10 | Unknown (pfam07098) |
| GTE6-*orf14* | 6302..7978 | 559 | hypothetical protein [*Salinispora pacifica*] | 30 | 6e-47 | Phage structural protein |
| GTE6-*orf15* | 7975..8361 | 129 | - | - | - | - |
| GTE6-*orf16* | 8361..10763 | 801 | capsid maturation protease [*Mycobacterium* phage Bernal13] | 43 | 3e-20 | Head morphogenesis (pfam04233) |
| GTE6-*orf17* | 10760..10981 | 74 | - | - | - | - |
| GTE6-*orf18* | 11027..13879 | 951 | gp12 [*Rhodococcus* phage ReqiPine5] | 56 | 1e-38 | RNA ligase (pfam13563) |
| GTE6-*orf19* | 14009..14155 | 49 | - | - | - | - |
| GTE6-*orf20* | 14261..16150 | 630 | hypothetical protein [*Streptomyces sulphureus*] | 33 | 6e-57 | Phage structural protein |
| GTE6-*orf21* | 16242..17072 | 277 | Epstein-Barr nuclear antigen 1 [*Saccharomonospora* phage PIS 136] | 35 | 8e-13 | Putative Eppstein-Barr nuclear antigen 1 |
| GTE6-*orf22* | 17086..17577 | 164 | - | - | - | - |
| GTE6-*orf23* | 17681..18376 | 232 | hypothetical protein [*Salinispora tropica*] | 39 | 7e-44 | Phage structural protein |
| GTE6-*orf24* | 18480..19277 | 266 | hypothetical protein [*Mycobacterium abscessus*] | 35 | 5e-30 | - |
| GTE6-*orf25* | 19274..19837 | 188 | hypothetical protein [*Salinispora tropica*] | 28 | 3e-04 | - |
| GTE6-*orf26* | 19842..20135 | 98 | - | - | - | - |
| GTE6-*orf27* | 20119..20550 | 144 | hypothetical protein [*Streptomyces sulphureus*] | 45 | 6e-18 | Putative tail component (pfam04883) |
| GTE6-*orf28* | 20594..21208 | 205 | - | - | - | - |
| GTE6-*orf29* | 21222..21899 | 226 | - | - | - | - |
| GTE6-*orf30* | 21946..27066 | 1707 | hypothetical protein [*Gordonia* sp. KTR9] | 33 | 3e-111 | Tape measure protein (pfam06737; COG5280) |
| GTE6-*orf31* | 27069..28046 | 326 | tail protein [*Mycobacterium intracellulare*] | 27 | 1e-11 | Putative tail protein |
| GTE6-*orf32* | 28048..29667 | 540 | hypothetical protein [*Mycobacterium abscessus*] | 30 | 2e-73 | - |
| GTE6-*orf33* | 29712..30401 | 230 | - | - | - | - |
| GTE6-*orf34* | 30398..31678 | 427 | hypothetical protein ISGA_1789 [*Gordonia* sp. NB4-1Y] | 51 | 4e-91 | - |
| GTE6-*orf35* | 31697..32035 | 113 | - | - | - | - |
| GTE6-*orf36* | 32032..33609 | 526 | hypothetical protein [*Rhodococcus equi*] | 38 | 4e-40 | - |
| GTE6-*orf37* | 33622..34326 | 235 | hypothetical protein [*Mycobacterium thermoresistibile*] | 33 | 2e-20 | - |
| GTE6-*orf38* | 34411..36045 | 545 | hypothetical protein [*Gordonia rhizosphera*] | 62 | 6e-112 | Lysin (pfam01510) |
| GTE6-*orf39* | 36058..36567 | 170 | hypothetical protein [*Rhodococcus equi*] | 42 | 8e-18 | Putative holin |
| GTE6-*orf40* | 36569..37060 | 164 | unnamed protein product [*Gordonia* phage GRU1] | 60 | 3e-48 | - |
| GTE6-*orf41* | 37057..37491 | 145 | hypothetical protein [*Gordonia sihwensis*] | 37 | 2e-22 | - |
| GTE6-*orf42* | 37596..38033 | 146 | - | - | - | - |
| GTE6-*orf43* | 38035..38283 | 83 | hypothetical protein [*Rhodococcus opacus*] | 45 | 2e-08 | - |
| GTE6-*orf44* | 38280..38465 | 62 | hypothetical protein GTE7_gp102 [*Gordonia* phage GTE7] | 41 | 6e-04 | - |
| GTE6-*orf45* | 38462..39616 | 385 | hypothetical protein [*Mycobacterium abscessus*] | 35 | 3e-44 | Recombinase (pfam09588) |
| GTE6-*orf46* | 39613..39906 | 98 | - | - | - | - |
| GTE6-*orf47* | 39899..40960 | 354 | recombinase RecT [*Mycobacterium avium*] | 47 | 2e-93 | Recombinase (pfam03837) |
| GTE6-*orf48* | 40982..41434 | 151 | - | - | - | - |
| GTE6-*orf49* | 41493..41639 | 49 | - | - | - | - |
| GTE6-*orf50* | 41632..41817 | 65 | hypothetical protein [*Allofustis seminis*] | 35 | 1e-04 | HTH DNA Binding (pfam12728) |
| GTE6-*orf51* | 41823..42017 | 65 | hypothetical protein [*Allofustis seminis*] | 35 | 1e-04 | HTH DNA Binding (pfam12728) |
| GTE6-*orf52* | 42014..42178 | 55 | - | - | - | - |
| GTE6-*orf53* | 42181..43176 | 332 | unnamed protein product [*Rhodococcus* phage REQ3] | 45 | 5e-18 | - |
| GTE6-*orf54* | 43173..43394 | 74 | hypothetical protein 32HC_68 [*Mycobacterium* phage 32HC] | 39 | 9e-04 | - |
| GTE6-*orf55* | 43476..43847 | 124 | - | - | - | - |
| GTE6-*orf56* | 43844..44005 | 54 | - | - | - | - |
| GTE6-*orf57* | 44002..44412 | 137 | - | - | - | Putative protein serine/threonine phosphatase (PRK14559) |
| GTE6-*orf58* | 44409..45188 | 260 | putative cutinase [*Gordonia* phage GTE2] | 36 | 1e-36 | Putative cutinase/lysin |
| GTE6-*orf59* | 45185..45565 | 127 | - | - | - | - |
| GTE6-*orf60* | 45562..46041 | 160 | - | - | - | - |
| GTE6-*orf61* | 46038..46202 | 55 | - | - | - | - |
| GTE6-*orf62* | 46212..46838 | 209 | RuvC [*Mycobacterium* phage Bernardo] | 33 | 2e-14 | Putative Holliday Junction Resolvase |
| GTE6-*orf63* | 46835..47095 | 87 | - | - | - | - |
| GTE6-*orf64* | 47088..47441 | 118 | - | - | - | - |
| GTE6-*orf65* | 47434..47742 | 103 | - | - | - | - |
| GTE6-*orf66* | 47732..48301 | 190 | - | - | - | - |
| GTE6-*orf67* | 48354..49775 | 474 | - | - | - | - |
| GTE6-*orf68* | 49772..50149 | 126 | - | - | - | - |
| GTE6-*orf69* | 50250..50750 | 167 | - | - | - | - |
| GTE6-*orf70* | 50747..51001 | 85 | - | - | - | - |
| GTE6-*orf71* | 51011..51286 | 92 | hypothetical protein E3_0905 [*Rhodococcus* phage E3] | 47 | 7e-12 | - |
| GTE6-*orf72* | 51283..51579 | 99 | - | - | - | - |
| GTE6-*orf73* | 51576..52175 | 200 | - | - | - | - |
| GTE6-*orf74* | 52172..52378 | 69 | - | - | - | - |
| GTE6-*orf75* | 52375..52836 | 154 | gp87 [*Mycobacterium* phage Anaya] | 47 | 1e-33 | Polynucleotide kinase (PHA02530) |
| GTE6-*orf76* | 52833..53069 | 79 | - | - | - | - |
| GTE6-*orf77* | 53066..53278 | 71 | - | - | - | - |
| GTE6-*orf78* | 53343..53906 | 188 | - | - | - | - |
| GTE6-*orf79* | 53903..54592 | 230 | DNA polymerase III subunit epsilon [*Rhodococcus* sp. P27] | 41 | 3e-48 | DNA Polymerase III epsilon subunit (pfam00929) |
| GTE6-*orf80* | 54589..54744 | 52 | - | - | - | - |
| GTE6-*orf81* | 54741..54896 | 52 | - | - | - | - |
| GTE6-*orf82* | 54893..55081 | 63 | - | - | - | - |
| GTE6-*orf83* | 55078..55488 | 137 | - | - | - | - |
| GTE6-*orf84* | 55485..55895 | 137 | - | - | - | - |
| GTE6-*orf85* | 55892..56542 | 217 | - | - | - | - |
| GTE6-*orf86* | 56599..56850 | 84 | hypothetical protein [*Streptomyces* sp. AW19M42] | 36 | 7e-05 | - |
| GTE8-*orf1* | 51..518 | 156 | unnamed protein product [*Gordonia* phage GRU1] | 51 | 3e-40 | - |
| GTE8-*orf2* | 515..745 | 77 | - | - | - | - |
| GTE8-*orf3* | 742..1041 | 100 | unnamed protein product [*Gordonia* phage GRU1] | 71 | 8e-41 | - |
| GTE8-*orf4* | 1049..1438 | 130 | unnamed protein product [*Gordonia* phage GRU1] | 35 | 9e-11 | - |
| GTE8-*orf5* | 1435..1704 | 90 | unnamed protein product [*Gordonia* phage GRU1] | 50 | 6e-18 | - |
| GTE8-*orf6* | 1966..2262 | 99 | unnamed protein product [*Gordonia* phage GTE5] | 59 | 1e-17 | - |
| GTE8-*orf7* | 2259..2990 | 244 | unnamed protein product [*Gordonia* phage GRU1] | 53 | 4e-74 | - |
| GTE8-*orf8* | 3094..3345 | 84 | unnamed protein product [*Gordonia* phage GTE5] | 89 | 2e-45 | - |
| GTE8-*orf9* | 3345..5333 | 663 | hypothetical protein [*Rhodococcus* sp. p52] | 58 | 0.0 | - |
| GTE8-*orf10* | 5351..5530 | 60 | unnamed protein product [*Gordonia* phage GTE5] | 91 | 1e-19 | - |
| GTE8-*orf11* | 5527..5994 | 156 | unnamed protein product [*Gordonia* phage GTE5] | 86 | 4e-93 | - |
| GTE8-*orf12* | 5991..6422 | 144 | terS gene product [*Gordonia* phage GTE5] | 88 | 5e-88 | Putative small terminase subunit (pfam01844) |
| GTE8-*orf13* | 6443..8170 | 576 | terL gene product [*Gordonia* phage GTE5] | 81 | 0.0 | Large terminase subunit (pfam03354) |
| GTE8-*orf14* | 8211..9800 | 530 | unnamed protein product [*Gordonia* phage GRU1] | 75 | 0.0 | Portal protein (pfam05133) |
| GTE8-*orf15* | 9797..11185 | 463 | unnamed protein product [*Gordonia* phage GTE5] | 72 | 0.0 | - |
| GTE8-*orf16* | 11182..11877 | 232 | unnamed protein product [*Gordonia* phage GRU1] | 57 | 6e75 | - |
| GTE8-*orf17* | 11895..12320 | 142 | unnamed protein product [*Gordonia* phage GRU1] | 69 | 1e-95 | Head decorator protein (pfam02924) |
| GTE8-*orf18* | 12360..13430 | 357 | unnamed protein product [*Gordonia* phage GTE5] | 74 | 0.0 | Major capsid structural protein (pfam03864) |
| GTE8-*orf19* | 13433..13885 | 151 | unnamed protein product [*Gordonia* phage GRU1] | 54 | 4e-40 | - |
| GTE8-*orf20* | 13932..14387 | 152 | unnamed protein product [*Gordonia* phage GTE5] | 75 | 3e-78 | - |
| GTE8-*orf21* | 14387..14779 | 131 | unnamed protein product [*Gordonia* phage GTE5] | 76 | 9e-66 | - |
| GTE8-*orf22* | 14772..15137 | 122 | unnamed protein product [*Gordonia* phage GTE5] | 76 | 1e-52 | - |
| GTE8-*orf23* | 15134..15649 | 172 | unnamed protein product [*Gordonia* phage GTE5] | 81 | 4e-97 | - |
| GTE8-*orf24* | 15678..16361 | 228 | unnamed protein product [*Gordonia* phage GRU1] | 89 | 1e-146 | Phage structural protein |
| GTE8-*orf25* | 16446..16829 | 128 | unnamed protein product [*Gordonia* phage GRU1] | 53 | 4e-33 | Major tail protein |
| GTE8-*orf26* | 16862..17176 | 105 | unnamed protein product [*Gordonia* phage GTE5] | 56 | 1e-34 | Putative tail assembly protein |
| GTE8-*orf27* | 17158..17667 | 170 | unnamed protein product [*Gordonia* phage GTE5] | 66 | 4e-61 | Putative tail assembly protein translated by conserved programmed translational frameshift |
| GTE8-*orf28* | 17751..23138 | 1796 | unnamed protein product [*Gordonia* phage GRU1] | 74 | 0.0 | Tape measure protein (COG5412; pfam06736) |
| GTE8-*orf29* | 23138..24079 | 314 | unnamed protein product [*Gordonia* phage GRU1] | 78 | 4e-172 | - |
| GTE8-*orf30* | 24084..25697 | 538 | unnamed protein product [*Gordonia* phage GRU1] | 85 | 0.0 | - |
| GTE8-*orf31* | 25738..26865 | 376 | unnamed protein product [*Gordonia* phage GTE5] | 77 | 0.0 | Phage structural protein |
| GTE8-*orf32* | 26866..29232 | 789 | unnamed protein product [*Gordonia* phage GRU1] | 83 | 0.0 | Carbohydrate binding domian (pfam02015) |
| GTE8-*orf33* | 29229..30287 | 353 | unnamed protein product [*Gordonia* phage GRU1] | 69 | 7e-112 | - |
| GTE8-*orf34* | 30263..30565 | 101 | unnamed protein product [*Gordonia* phage GRU1] | 78 | 2e-49 | - |
| GTE8-*orf35* | 30569..30913 | 115 | unnamed protein product [*Gordonia* phage GTE5] | 78 | 1e-57 | - |
| GTE8-*orf36* | 30971..31603 | 211 | unnamed protein product [*Gordonia* phage GRU1] | 89 | 1e-137 | Lysin - Peptidase (pfam13529) |
| GTE8-*orf37* | 31603..32625 | 341 | unnamed protein product [*Gordonia* phage GRU1] | 80 | 0.0 | Lysin - Chitinase (pfam00182) |
| GTE8-*orf38* | 32622..32933 | 104 | unnamed protein product [*Gordonia* phage GTE5] | 57 | 2e-33 | Putative holin |
| GTE8-*orf39* | 33002..33436 | 145 | unnamed protein product [*Gordonia* phage GRU1] | 61 | 1e-55 | - |
| GTE8-*orf40* | 33411..33839 | 143 | unnamed protein product [*Gordonia* phage GTE5] | 81 | 6e-77 | Portal vertex protein (PHA02531) |
| GTE8-*orf41* | complement(33836..36385) | 850 | unnamed protein product [*Gordonia* phage GRU1] | 71 | 0.0 | Bifunctional primase/polymerase (pfam09250; COG3378) |
| GTE8-*orf42* | complement(36397..36801) | 135 | unnamed protein product [*Gordonia* phage GRU1] | 44 | 6e-35 | - |
| GTE8-*orf43* | complement(36758..37105) | 116 | - | - | - | - |
| GTE8-*orf44* | complement(37102..37374) | 91 | unnamed protein product [*Gordonia* phage GRU1] | 68 | 5e-38 | - |
| GTE8-*orf45* | complement(37371..37847) | 159 | unnamed protein product [*Gordonia* phage GTE5] | 51 | 1e-40 | DNA Binding (pfam12728) |
| GTE8-*orf46* | complement(37878..38609) | 244 | unnamed protein product [*Gordonia* phage GRU1] | 56 | 2e-88 | - |
| GTE8-*orf47* | complement(38606..38830) | 75 | unnamed protein product [*Gordonia* phage GRU1] | 46 | 1e-10 | - |
| GTE8-*orf48* | complement(38830..42138) | 1103 | unnamed protein product [*Gordonia* phage GTE5] | 74 | 0.0 | DNA Polymerase II alpha subunit (COG0587) |
| GTE8-*orf49* | complement(42135..42431) | 99 | - | - | - | - |
| GTE8-*orf50* | complement(42428..42589) | 54 | hypothetical protein [*Rhodococcus fascians*] | 44 | 5e-06 | - |
| GTE8-*orf51* | complement(42710..42940) | 77 | unnamed protein product [*Gordonia* phage GRU1] | 71 | 7e-28 | - |
| GTE8-*orf52* | complement(42947..43411) | 155 | unnamed protein product [*Gordonia* phage GRU1] | 37 | 1e-17 | - |
| GTE8-*orf53* | complement(43535..44425) | 297 | unnamed protein product [*Gordonia* phage GRU1] | 52 | 2e-61 | - |
| GTE8-*orf54* | complement(44458..45279) | 274 | unnamed protein product [*Gordonia* phage GTE5] | 61 | 3e-99 | AAAprotein (pfam13479) |
| GTE8-*orf55* | complement(45335..46534) | 400 | unnamed protein product [*Gordonia* phage GRU1] | 71 | 0.0 | - |
| GTE8-*orf56* | complement(46531..46842) | 104 | - | - | - | - |
| GTE8-*orf57* | complement(46880..47056) | 59 | unnamed protein product [*Gordonia* phage GTE5] | 76 | 9e-09 | - |
| GTE8-*orf58* | complement(47053..47388) | 112 | unnamed protein product [*Gordonia* phage GTE5] | 66 | 1e-44 | - |
| GTE8-*orf59* | complement(47385..47633) | 83 | - | - | - | - |
| GTE8-*orf60* | complement(47633..49561) | 643 | unnamed protein product [*Gordonia* phage GRU1] | 76 | 0.0 | Helicase (COG0553) |
| GTE8-*orf61* | complement(49612..50187) | 192 | unnamed protein product [*Gordonia* phage GTE5] | 34 | 5e-12 | - |
| GTE8-*orf62* | complement(50184..50369) | 62 | unnamed protein product [*Gordonia* phage GRU1] | 49 | 6e-07 | - |
| GTE8-*orf63* | complement(50412..50618) | 69 | - | - | - | - |
| GTE8-*orf64* | complement(50618..50743) | 42 | unnamed protein product [*Gordonia* phage GRU1] | 63 | 6e-10 | - |
| GTE8-*orf65* | complement(50740..51213) | 158 | unnamed protein product [*Gordonia* phage GRU1] | 83 | 5e-95 | - |
| GTE8-*orf66* | complement(51213..51692) | 160 | - | - | - | Cytosolic phospholipase (cd7201) |
| GTE8-*orf67* | complement(51696..52721) | 342 | unnamed protein product [*Gordonia* phage GRU1] | 65 | 2e-146 | - |
| GTE8-*orf68* | 53037..53228 | 64 | - | - | - | - |
| GTE8-*orf69* | 53292..54173 | 294 | - | - | - | - |
| GTE8-*orf70* | 54170..54565 | 132 | unnamed protein product [*Gordonia* phage GRU1] | 52 | 5e-34 | - |
| GTE8-*orf71* | 54601..54894 | 98 | - | - | - | - |
| GTE8-*orf72* | 54913..55671 | 253 | - | - | - | - |
| GTE8-*orf73* | 55760..56416 | 219 | - | - | - | - |
| GTE8-*orf74* | 56418..56585 | 56 | unnamed protein product [*Gordonia* phage GRU1] | 64 | 9e-14 | - |
| GTE8-*orf75* | 56582..57067 | 162 | gp85 [*Mycobacteriophage Astro*] | 30 | 1e-06 | - |
| GTE8-*orf76* | 57064..57753 | 230 | unnamed protein product [*Gordonia* phage GRU1] | 47 | 6e-51 | - |
| GTE8-*orf77* | 57782..58288 | 169 | - | - | - | - |
| GTE8-*orf78* | 58285..58428 | 48 | - | - | - | - |
| GTE8-*orf79* | 58496..58765 | 90 | unnamed protein product [*Gordonia* phage GTE5] | 57 | 1e-12 | - |
| GTE8-*orf80* | 58798..59136 | 113 | - | - | - | - |
| GTE8-*orf81* | 59310..60392 | 361 | hypothetical protein GOALK_093_00330 [Gordonia alkanivorans NBRC 16433] | 37 | 9e-23 | HNH endonuclease (pfam01844) |
| GTE8-*orf82* | 60392..60949 | 186 | unnamed protein product [*Gordonia* phage GRU1] | 68 | 1e-72 | - |
| GTE8-*orf83* | 61537..61938 | 134 | - | - | - | - |
| GTE8-*orf84* | 61935..63851 | 639 | unnamed protein product [*Gordonia* phage GRU1] | 78 | 2e-07 | - |
| GTE8-*orf85* | 63971..64687 | 239 | unnamed protein product [*Gordonia* phage GRU1] | 69 | 7e-104 | - |
| GTE8-*orf86* | 64780..64983 | 68 | unnamed protein product [*Gordonia* phage GTE5] | 75 | 4e-23 | - |
| GTE8-*orf87* | 64976..65326 | 117 | - | - | - | - |
| GTE8-*orf88* | 65323..65595 | 91 | - | - | - | - |
| GTE8-*orf89* | 65585..65725 | 47 | - | - | - | - |
| GTE8-*orf90* | 65718..65939 | 74 | - | - | - | - |
| GTE8-*orf91* | 66184..66642 | 153 | unnamed protein product [*Gordonia* phage GRU1] | 84 | 1e-90 | - |
| GTE8-*orf92* | 66639..66941 | 101 | - | - | - | - |
| GTE8-*orf93* | 66938..67309 | 124 | unnamed protein product [*Gordonia* phage GTE5] | 33 | 2e-07 | - |
| GTE8-*orf94* | 67306..67617 | 104 | - | - | - | - |
| GRU3-*orf1* | 65..502 | 146 | hypothetical protein [*Corynebacterium diphtheriae*] | 36 | 2e-09 | Putative small terminase subunit |
| GRU3-*orf2* | 495..1916 | 474 | putative phage terminase protein [*Gordonia neofelifaecis*] | 54 | 1e-133 | Large terminase subunit (pfam03237) |
| GRU3-*orf3* | 1924..2109 | 62 | hypothetical protein [*Gordonia neofelifaecis*] | 48 | 4e-08 | - |
| GRU3-*orf4* | 2171..3247 | 359 | hypothetical protein [*Gordonia neofelifaecis*] | 66 | 7e-148 | Portal structural protein (pfam04860) |
| GRU3-*orf5* | 3244..4032 | 263 | hypothetical protein [*Gordonia soli*] | 51 | 2e-65 | Lysin - D-alanyl-D-alanine carboxypeptidase (pfam13539) |
| GRU3-*orf6* | 4133..6103 | 657 | hypothetical protein [*Gordonia neofelifaecis*] | 60 | 0.0 | Caudovirales prohead protease structural protein (pfam04586) |
| GRU3-*orf7* | 6107..6448 | 114 | unnamed protein product [*Rhodococcus* phage RRH1] | 45 | 1e-19 | - |
| GRU3-*orf8* | 6449..6787 | 113 | hypothetical protein [*Gordonia neofelifaecis*] | 58 | 5e-35 | - |
| GRU3-*orf9* | 6800..7261 | 154 | hypothetical protein [*Gordonia neofelifaecis*] | 75 | 6e-76 | - |
| GRU3-*orf10* | 7270..7608 | 113 | hypothetical protein [*Gordonia neofelifaecis*] | 51 | 2e-24 | Phage protein (TIGR01725) |
| GRU3-*orf11* | 7630..7923 | 98 | hypothetical protein [*Gordonia neofelifaecis*] | 68 | 1e-39 | - |
| GRU3-*orf12* | 8095..9960 | 622 | TP901 family phage tail tape measure protein, putative [*Gordonia neofelifaecis*] | 58 | 0.0 | Tape measure protein |
| GRU3-*orf13* | 9957..11411 | 485 | hypothetical protein [*Nocardia otitidiscaviarum*] | 31 | 7e-41 | - |
| GRU3-*orf14* | 11426..12031 | 202 | hypothetical protein [*Nocardia otitidiscaviarum*] | 48 | 5e-53 | - |
| GRU3-*orf15* | 12090..12938 | 283 | bacteriophage protein [*Mycobacterium thermoresistibile*] | 42 | 2e-60 | PE-PPE structural protein (pfam08237) |
| GRU3-*orf16* | complement(13012..13116) | 35 | - | - | - | - |
| GRU3-*orf17* | complement(13195..14088) | 298 | integrase [*Gordonia neofelifaecis*] | 70 | 2e-124 | Integrase (pfam00589) |
| GRU3-*orf18* | complement(14162..14485) | 108 | putative DNA-binding protein [*Gordonia neofelifaecis*] | 54 | 7e-28 | HTH DNA binding (pfam12844) |
| GRU3-*orf19* | complement(14648..14773) | 42 | - | - | - | - |
| GRU3-*orf20* | 14761..14949 | 63 | hypothetical protein [*Rhodococcus fascians*] | 55 | 9e-08 | HTH DNA binding (pfam12728) |
| GRU3-*orf21* | 14946..15404 | 153 | - | - | - | - |
| GRU3-*orf22* | 15404..15646 | 81 | hypothetical protein [*Gordonia neofelifaecis*] | 38 | 6e-05 | - |
| GRU3-*orf23* | 15646..15870 | 75 | - | - | - | - |
| GRU3-*orf24* | 15858..16127 | 90 | - | - | - | - |
| GRU3-*orf25* | 16472..17227 | 252 | DNA polymerase III subunit epsilon [*Gordonia neofelifaecis*] | 56 | 1e-155 | DNA polymerase III subunit epsilon (COG0847) |
| GRU3-*orf26* | 17220..17402 | 61 | - | - | - | - |
|  |  |  |  |  |  |  |
|  |  |  |  |  |  |  |
|  |  |  |  |  |  |  |
|  |  |  |  |  |  |  |
|  |  |  |  |  |  |  |
|  |  |  |  |  |  |  |

^a^ ORFs were numbered consecutively, ^b^ The most closely related gene (only if named) and the name of the organism, ^c^ Percentage identity is based on the best match when a BLAST P analysis is performed, ^d^ The probability of obtaining a match by chance as determined by BLAST analysis. Only values less than 10^-4^ were considered significant, ^e^ Predicted function is based on amino acid identity, conserved motifs, and gene location within functional modules.
