# Supplementary material for: Lysis to Kill: Evaluation of the Lytic Abilities, and Genomics of Nine Bacteriophages Infective for Gordonia spp. and Their Potential Use in Activated Sludge Foam Biocontrol
Source: PLoS One. 2015 Aug 4;10(8):e0134512. doi: 10.1371/journal.pone.0134512 (PMC4524720; doi:10.1371/journal.pone.0134512)
Supplement: S2 Table — (DOCX) [file pone.0134512.s002.docx]

**Table S2: Putative tRNA detected in *Gordonia* spp. phage genomes**

| Phage | No. tRNA | tRNA present | Coordinates | Size (bp) | G+C (mol %) |
| --- | --- | --- | --- | --- | --- |
| GMA2 | 16 | tRNA-Thr(tgt) | Complement(57883..57955) | 73 | 52.1 |
|  |  | tRNA-Glu(ttc) | Complement(58076..58153) | 78 | 59.0 |
|  |  | tRNA-Ser(gct) | Complement(58157..58240) | 84 | 58.3 |
|  |  | tRNA-Ser(tga) | Complement(58319..58403) | 85 | 54.1 |
|  |  | tRNA-Lys(ctt) | Complement(58406..58478) | 73 | 53.4 |
|  |  | tRNA-Lys(ttt) | Complement(58481..58552) | 72 | 58.3 |
|  |  | tRNA-Glu(ctc) | Complement(58733..58806) | 74 | 48.6 |
|  |  | tRNA-Leu(tag) | Complement(58894..58978) | 85 | 55.3 |
|  |  | tRNA-Asp(gtc) | Complement(58993..59068) | 76 | 57.9 |
|  |  | tRNA-Tyr(gta) | Complement(59077..59160) | 84 | 58.3 |
|  |  | tRNA-Pro(tgg) | Complement(59301..59375) | 75 | 49.3 |
|  |  | tRNA-Ile(gat) | Complement(59483..59557) | 75 | 52.0 |
|  |  | tRNA-Met(cat) | Complement(59632..59706) | 75 | 54.7 |
|  |  | tRNA-Asn(gtt) | Complement(59744..59816) | 73 | 53.4 |
|  |  | tRNA-Gln(ttg) | Complement(59821..59895) | 75 | 62.7 |
|  |  | tRNA-Trp(cca) | Complement(60083..60154) | 72 | 51.4 |
| GMA4 | 1 | tRNA-Try(gta) | 5132..5213 | 82 | 62.2 |
| GMA7 | 1 | tRNA-Asn (gtt) | 13362..13435 | 74 | 47.3 |
