# Supplementary material for: Lysis to Kill: Evaluation of the Lytic Abilities, and Genomics of Nine Bacteriophages Infective for Gordonia spp. and Their Potential Use in Activated Sludge Foam Biocontrol
Source: PLoS One. 2015 Aug 4;10(8):e0134512. doi: 10.1371/journal.pone.0134512 (PMC4524720; doi:10.1371/journal.pone.0134512)
Supplement: S3 Table — (DOCX) [file pone.0134512.s003.docx]

**Table S3: Palindromes in the genome sequence of 9 *Gordonia* spp. phages**

| Phage-Repeat number | Size (bp) | Coordinates | Sequence alignment |
| --- | --- | --- | --- |
| GMA2-P1 | 48 | 57783-57830 | GAGCTGGAGATGATCTCAATCGTAGATGATAGAAATCATCTCCAGCTC |
|  |  | 57830-57783 | GAGCTGGAGATGATTTCTATCATCTACGATTGAGATCATCTCCAGCTC |
| GMA2-P2 | 47 | 60886-60930 | AGAGGATGCCGATAGACGCGACGATATCACGTCTATCGGC--CCTCT |
|  |  | 60930-60886 | AGAGG--GCCGATAGACGTGATATCGTCGCGTCTATCGGCATCCTCT |
| GMA2-P3 | 43 | 62824-62866 | TCACGTCTTCGGTCTAAAGTAAACCCTTAGACCGAAGACGAGA |
|  |  | 62866-62824 | TCTCGTCTTCGGTCTAAGGGTTTACTTTAGACCGAAGACGTGA |
| GMA2-P4 | 39 | 50539-50577 | AATCTACGTTGAACGAGCTTGTTTCGTTCAACGTAGATT |
|  |  | 50577-50539 | AATCTACGTTGAACGAAACAAGCTCGTTCAACGTAGATT |
| GMA2-P5 | 32 | 47948-47979 | CTCTACACGCGAGCCGAAGCTCGCGTGTAGAG |
|  |  | 47979-47948 | CTCTACACGCGAGCTTCGGCTCGCGTGTAGAG |
| GMA2-P6 | 16 | 15010-15025 | AGAATCACGTGATTCT |
|  |  | 15025-15010 | AGAATCACGTGATTCT |
| GMA2-P7 | 16 | 22496-22511 | CGTAGCCGCGGCTACG |
|  |  | 22511-22496 | CGTAGCCGCGGCTACG |
| GMA3-P1 | 98 | 10371-10467 | AAGAGACCACTTAGACAGGATGCAATCTAAAATGGCCGATGTATGGAGCAATTCGTATTTTGACGATTTTAGTGGGGA-CCTGTCTAAGTGGTCTTTT |
|  |  | 10467-10371 | AAAAGACCACTTAGACAGGTCCCCA-CTAAAATCGTCAAAATACGAATTGCTCCATACATCGGCCATTTTAGATTGCATCCTGTCTAAGTGGTCTCTT |
| GMA3-P2 | 66 | 52315-52378 | GCGATCCCCGATAATTGCTAGGCAC--TTGGGGCCTTAAAGGGGAATGACAATTATCGGGGATCGC |
|  |  | 52378-52315 | GCGATCCCCGATAATTGTCATTCCCCTTTAAGGCCCCAA--GTGCCTAGCAATTATCGGGGATCGC |
| GMA3-P3 | 51 | 58726-58774 | CCGAAGGCGCAGGCTTTGGCAGATTAAATCTGTTA--GCCTGCGCCTTCGG |
|  |  | 58774-58726 | CCGAAGGCGCAGGCTAA--CAGATTTAATCTGCCAAAGCCTGCGCCTTCGG |
| GMA3-P4 | 50 | 33805-33854 | TGCCAGTCCACGGTAAATCCCCTTTGCTGGGGGCATACCGTGGACTGGCA |
|  |  | 33854-33805 | TGCCAGTCCACGGTATGCCCCCAGCAAAGGGGATTTACCGTGGACTGGCA |
| GMA3-P5 | 48 | 13807-13853 | GGATTATCCCCATGTGCCAAG-TGCAGATTGGCACATGGGGATAATCC |
|  |  | 13853-13807 | GGATTATCCCCATGTGCCAATCTGCAC-TTGGCACATGGGGATAATCC |
| GMA3-P6 | 45 | 36860-36904 | CAAAAGGGCGTCGTACGGACATTTTACTCGTACGACGCCCTTTTG |
|  |  | 36904-36860 | CAAAAGGGCGTCGTACGAGTAAAATGTCCGTACGACGCCCTTTTG |
| GMA3-P7 | 44 | 67284-67327 | ACAAAAAATCCCCTACCGCGTGGATGCGGTAGGGGATTTTTTGT |
|  |  | 67327-67284 | ACAAAAAATCCCCTACCGCATCCACGCGGTAGGGGATTTTTTGT |
| GMA3-P8 | 44 | 51946-51987 | TAAGGCTAAGCCGGGAGAAATTAAT--CTCCCGGCTTAGCCTTA |
|  |  | 51987-51946 | TAAGGCTAAGCCGGGAGA--TTAATTTCTCCCGGCTTAGCCTTA |
| GMA3-P9 | 42 | 27139-27180 | ATCCTGGTCGGAGGAAATGTTAATGTTTCCTCCGACCAGGAT |
|  |  | 27180-27139 | ATCCTGGTCGGAGGAAACATTAACATTTCCTCCGACCAGGAT |
| GMA3-P10 | 39 | 39354-39392 | AAAAATACCCGGCACCATGAATTGGTGCCGGGTATTTTT |
|  |  | 39392-39354 | AAAAATACCCGGCACCAATTCATGGTGCCGGGTATTTTT |
| GMA3-P11 | 36 | 71487-71522 | ATGAATGCATATGCATCACTATGCATATGCATTCAT |
|  |  | 71522-71487 | ATGAATGCATATGCATAGTGATGCATATGCATTCAT |
| GMA3-P12 | 36 | 68183-68218 | GTCGACCAGCGAAATTTCCTGTTTTCGCTGGTCGAC |
|  |  | 68218-68183 | GTCGACCAGCGAAAACAGGAAATTTCGCTGGTCGAC |
| GMA3-P13 | 26 | 72043-72068 | GTACCGATAACACTTGTTATCGGTAC |
|  |  | 72068-72043 | GTACCGATAACAAGTGTTATCGGTAC |
| GMA3-P14 | 26 | 72532-72557 | AGAAAAAAATATATATATTTATTCCT |
|  |  | 72557-72532 | AGGAATAAATATATATATTTTTTTCT |
| GMA3-P15 | 16 | 6982-6997 | CTCGTTTATAAACGAG |
|  |  | 6997-6982 | CTCGTTTATAAACGAG |
| GMA3-P16 | 16 | 20089-20104 | TTGACAATATTGTCAA |
|  |  | 20104-20089 | TTGACAATATTGTCAA |
| GMA4-P1 | 56 | 22342-22395 | TGAACG--GAAATGCGCCCCAACCTCTTCGGAGGTTGGGGCGCATTTCTGCGTTCA |
|  |  | 22395-22342 | TGAACGCAGAAATGCGCCCCAACCTCCGAAGAGGTTGGGGCGCATTTC--CGTTCA |
| GMA4-P2 | 46 | 7033-7075 | CGCGTCGAACCA---GGTGCCCGCGGGCACCGCGTGGGTCGTCGCG |
|  |  | 7075-7033 | CGCGACGACCCACGCGGTGCCCGCGGGCACC---TGGTTCGACGCG |
| GMA4-P3 | 22 | 8283-8304 | CGCGGCGATCATGATCGCCGCG |
|  |  | 8304-8283 | CGCGGCGATCATGATCGCCGCG |
| GMA4-P4 | 20 | 10274-10293 | CGAACTCGTCGACGAGTTCG |
|  |  | 10293-10274 | CGAACTCGTCGACGAGTTCG |
| GMA4-P5 | 16 | 14873-14888 | TCGGCGGATCCGCCGA |
|  |  | 14888-14873 | TCGGCGGATCCGCCGA |
| GMA4-P6 | 16 | 33433-33448 | GCTGGCCGCGGCCAGC |
|  |  | 33448-33433 | GCTGGCCGCGGCCAGC |
| GMA5-P1 | 42 | 8066-8107 | CCGCCGCAGCGGCCCCGGCCGCCGCCGGGGCCGCTCAGGCGG |
|  |  | 8107-8066 | CCGCCTGAGCGGCCCCGGCGGCGGCCGGGGCCGCTGCGGCGG |
| GMA5-P2 | 41 | 12951-12990 | GACGCCCCGCGCCA-TCCTTGGGGGAGTGGCGCGGGGCGTC |
|  |  | 12990-12951 | GACGCCCCGCGCCACTCCCCCAAGGA-TGGCGCGGGGCGTC |
| GMA5-P3 | 37 | 15912-15947 | GCCGTTGAGGACCGCCGTGACG-CGATCCTCAACGGC |
|  |  | 15947-15912 | GCCGTTGAGGATCGC-GTCACGGCGGTCCTCAACGGC |
| GMA5-P4 | 32 | 14428-14459 | CACGCCCCGTCACCATCCGGTGACGGGGCGTG |
|  |  | 14459-14428 | CACGCCCCGTCACCGGATGGTGACGGGGCGTG |
| GMA5-P5 | 30 | 6072-6101 | ACCCGGCGACCGTTGACGCGGTCGCCGGGT |
|  |  | 6101-6072 | ACCCGGCGACCGCGTCAACGGTCGCCGGGT |
| GMA5-P6 | 16 | 16425-16440 | CTCAGCGGCCGCTGAG |
|  |  | 16440-16425 | CTCAGCGGCCGCTGAG |
| GMA5-P7 | 14 | 1507-1520 | GTCGCGATCGCGAC |
|  |  | 1520-1507 | GTCGCGATCGCGAC |
| GMA5-P8 | 14 | 3061-3074 | GGACGACGTCGTCC |
|  |  | 3074-3061 | GGACGACGTCGTCC |
| GMA5-P9 | 14 | 6311-6324 | GCGACGTACGTCGC |
|  |  | 6324-6311 | GCGACGTACGTCGC |
| GMA5-P10 | 14 | 6580-6593 | AACGGCATGCCGTT |
|  |  | 6593-6580 | AACGGCATGCCGTT |
| GMA5-P11 | 14 | 13965-13978 | GCCGCGATCGCGGC |
|  |  | 13978-13965 | GCCGCGATCGCGGC |
| GMA6-P1 | 58 | 58978-59034 | TGAGTAAAGCCGGGGTAGGTG-CGCGTGGGGATGCGTAATCTACTCCGGCTTTACTCA |
|  |  | 59034-58978 | TGAGTAAAGCCGGAGTAGATTACGCATCCCCACGCGCAC-CTACCCCGGCTTTACTCA |
| GMA7-P1 | 90 | 62906-62992 | TCAATAGATCAGGTGGCCGGTGGGAAATGCT---AACGGTTACCAGCGAAAGCATTTCGATCAGTGCCCACCGGCCACCTGATATATTGA |
|  |  | 62992-62906 | TCAATATATCAGGTGGCCGGTGGGCACTGATCGAAATGCTTTCGCTGGTAACCGTT---AGCATTTCCCACCGGCCACCTGATCTATTGA |
| GMA7-P2 | 83 | 14335-14413 | TAATACACATAAAGGGTAGTGCTTG-AATAGCACTACCCTTTATGTGTATT---ATTGGCATCACTACCCTTTATGTGTATTA |
|  |  | 14413-14335 | TAATACACATAAAGGGTAGTGATGCCAATAA---TACACATAAAGGGTAGTGCTATTCA-AGCACTACCCTTTATGTGTATTA |
| GMA7-P3 | 52 | 38198-38246 | ACCAACGGGTAGCGTATCA---TGCCGACACATTCATACGCTACCCGTTGGT |
|  |  | 38246-38198 | ACCAACGGGTAGCGTATGAATGTGTCGGCA---TGATACGCTACCCGTTGGT |
| GMA7-P4 | 51 | 14335-14385 | TAATACACATAAAGGGTAGTGCTTGAATAGCACTACCCTTTATGTGTATTA |
|  |  | 14385-14335 | TAATACACATAAAGGGTAGTGCTATTCAAGCACTACCCTTTATGTGTATTA |
| GMA7-P5 | 49 | 10130-10178 | CAAGAAATGCCCCGCCTGGACTACCAAAGCCCAGTCGGGGCATTTCTTG |
|  |  | 10178-10130 | CAAGAAATGCCCCGACTGGGCTTTGGTAGTCCAGGCGGGGCATTTCTTG |
| GMA7-P6 | 48 | 53858-53904 | AAAAATCAGGCACGAGCAGTGATCCAT-ACTGCTCGTGCCTGATTGTT |
|  |  | 53904-53858 | AACAATCAGGCACGAGCAGT-ATGGATCACTGCTCGTGCCTGATTTTT |
| GMA7-P7 | 47 | 64164-64210 | TGCATTCGTTCGTGAACTAACTATAACTCGGTCCACGAACGAATGCA |
|  |  | 64210-64164 | TGCATTCGTTCGTGGACCGAGTTATAGTTAGTTCACGAACGAATGCA |
| GMA7-P8 | 38 | 47553-47590 | CAATTGAAGGGGCTGCATCGGTGCAGCCCCTTCAATTG |
|  |  | 47590-47553 | CAATTGAAGGGGCTGCACCGATGCAGCCCCTTCAATTG |
| GMA7-P9 | 38 | 63072-63109 | GTGCACCTCCCCCAACCCCGAGAATAGGGGAGGTGCAC |
|  |  | 63109-63072 | GTGCACCTCCCCTATTCTCGGGGTTGGGGGAGGTGCAC |
| GMA7-P10 | 36 | 3460-3494 | GCGAACTAGCGGTGTGCTAG-ACACCGCTAGTTCGC |
|  |  | 3494-3460 | GCGAACTAGCGGTGT-CTAGCACACCGCTAGTTCGC |
| GMA7-P11 | 36 | 65620-65655 | CACTAACTACATGGTACCACGCCCATGTAGTTAGTG |
|  |  | 65655-65620 | CACTAACTACATGGGCGTGGTACCATGTAGTTAGTG |
| GMA7-P12 | 32 | 30692-30723 | CCCCAGCACCGATCCCAGAATCGGTGCTGGGG |
|  |  | 30723-30692 | CCCCAGCACCGATTCTGGGATCGGTGCTGGGG |
| GMA7-P13 | 30 | 236-264 | AAACTTTC-ACGGATATCCGTGGAAAGCTT |
|  |  | 264-236 | AAGCTTTCCACGGATATCCGT-GAAAGTTT |
| GMA7-P14 | 26 | 69409-69434 | CGGACACCAGCTCGAGCTGTCGTCCG |
|  |  | 69434-69409 | CGGACGACAGCTCGAGCTGGTGTCCG |
| GMA7-P15 | 24 | 7474-7497 | CTCTGGCGAAACGTTTCGGCAGAG |
|  |  | 7497-7474 | CTCTGCCGAAACGTTTCGCCAGAG |
| GMA7-P16 | 16 | 24291-24306 | GCCTTGTCGACAAGGC |
|  |  | 24306-24291 | GCCTTGTCGACAAGGC |
| GMA7-P17 | 16 | 63264-63279 | CACCCGAGCTCGGGTG |
|  |  | 63279-63264 | CACCCGAGCTCGGGTG |
| GMA7-P18 | 16 | 66284-66299 | CGGATGATATCATCCG |
|  |  | 66299-66284 | CGGATGATATCATCCG |
| GRU3-P1 | 34 | 14234-14267 | ACATGCCCCGTCACCATCCGGTGACGGGGCATGT |
|  |  | 14267-14234 | ACATGCCCCGTCACCGGATGGTGACGGGGCATGT |
| GRU3-P2 | 14 | 9980-9993 | CGTCGAGCTCGACG |
|  |  | 9993-9980 | CGTCGAGCTCGACG |
| GRU3-P3 | 14 | 13976-13989 | GCCGCTATAGCGGC |
|  |  | 13989-13976 | GCCGCTATAGCGGC |
| GTE6-P1 | 42 | 18413-18454 | CGAATGCCGCACGACCGGTTCACGCTGGTCGTGCGGCATTCG |
|  |  | 18454-18413 | CGAATGCCGCACGACCAGCGTGAACCGGTCGTGCGGCATTCG |
| GTE6-P2 | 22 | 21210-21231 | CGAGGGCCACGCGTGGCGCTCG |
|  |  | 21231-21210 | CGAGCGCCACGCGTGGCCCTCG |
| GTE6-P3 | 20 | 41544-41563 | GTCAACCGGCGCCGGTTCAC |
|  |  | 41563-41544 | GTGAACCGGCGCCGGTTGAC |
| GTE8-P1 | 45 | 32958-33002 | CCCGGACCGGCAGGTGGACTCCCCTCATCCCCCTGCCGGTCCGGG |
|  |  | 33002-32958 | CCCGGACCGGCAGGGGGATGAGGGGAGTCCACCTGCCGGTCCGGG |
| GTE8-P2 | 40 | 63863-63900 | TAGCGCCGGGTGTCTGG-GTACGCCAG-CACCCGGCGCTA |
|  |  | 63900-63863 | TAGCGCCGGGTG-CTGGCGTAC-CCAGACACCCGGCGCTA |
| GTE8-P3 | 36 | 43435-43470 | AGCGGGTGGACCGACCCCTCAATCGGTCCACCCGCT |
|  |  | 43470-43435 | AGCGGGTGGACCGATTGAGGGGTCGGTCCACCCGCT |
| GTE8-P4 | 28 | 8229-8256 | CAATTGGTACGCGCGAACGTACCAATTG |
|  |  | 8256-8229 | CAATTGGTACGTTCGCGCGTACCAATTG |
| GTE8-P5 | 16 | 26433-26448 | GTTCACCGCGGTGAAC |
|  |  | 26448-26433 | GTTCACCGCGGTGAAC |
