# Supplementary material for: Lysis to Kill: Evaluation of the Lytic Abilities, and Genomics of Nine Bacteriophages Infective for Gordonia spp. and Their Potential Use in Activated Sludge Foam Biocontrol
Source: PLoS One. 2015 Aug 4;10(8):e0134512. doi: 10.1371/journal.pone.0134512 (PMC4524720; doi:10.1371/journal.pone.0134512)
Supplement: S4 Table — I indicates inverted repeat, D indicates direct repeat. (DOCX) [file pone.0134512.s004.docx]

**Table S4: Repeats in the genome sequences of 9 *Gordonia* spp. phages.** I indicates inverted repeat, D indicates direct repeat.

| Phage-Repeat number | Size (bp) | Coordinates | Sequence alignment |
| --- | --- | --- | --- |
| GMA2-I1 | 29 | 94100-94128 | CTTCACGGATGGCAGCGATGCCAGTCTTC |
|  |  | 26333-26305 | CTTCCTGGATGTCAGAGATGCCAGTCTTC |
| GMA2-I2 | 23 | 65331-65353 | AGCGCAAGATCGCTCTGAGAGGC |
|  |  | 43697-43675 | AGCGCTAGATCGCTCTGAGACGC |
| GMA2-I3 | 23 | 61804-61826 | TGAGCGCTGCAGTAGGCAGCATC |
|  |  | 50397-50376 | TGAG-GCTACAGTAGGCAGCATC |
| GMA2-I4 | 22 | 66679-66700 | AACCATCAGCTCAGTGACGGTG |
|  |  | 7193-7172 | AACGATCGGCTCAGTGACGGTG |
| GMA2-I5 | 21 | 77503-77523 | AGCGCGATCATTCGGTCTTCG |
|  |  | 10181-10161 | AGCGCGATCATTCCGTCTTCG |
| GMA2-I6 | 20 | 90351-90370 | GAATGTCTGCACAGCCAGCA |
|  |  | 84922-84903 | GAATGTCTGCACAGCCATCA |
| GMA2-I7 | 19 | 21963-21981 | CCAGGTGCTGAACTCGTCC |
|  |  | 1410-1392 | CCAGGTGATGAACTCGTCC |
| GMA2-I8 | 19 | 70949-70967 | ACATGATCGTGTCAACTAC |
|  |  | 4007-3989 | ACATGAACGTGTCAACTAC |
| GMA2-I9 | 16 | 102972-102987 | CGTTTGGCAGCTTCTT |
|  |  | 28481-28466 | CGTTTGGCAGCTTCTT |
| GMA2-I10 | 16 | 46675-46690 | ACGCGGATGCCGAAAT |
|  |  | 44178-44163 | ACGCGGATGCCGAAAT |
| GMA2-D1 | 89 | 30422-30510 | CTCAAGACTCCGCCAATTGACCGCAGCAAGCCACTTGAGCCTGAGGTTGTAGACCCAGACATCAAGGAGCCTAAGACTGACGATAAGAC |
|  |  | 29963-30051 | CTCAAGACTCCGCCAATTGACCGCAGCAAGCCACTTGAGCCTGAGGTTGTAGACCCAGACATCAAGGAGCCTAAGACTGACGATAAGAC |
| GMA2-D2 | 80 | 37181-37260 | GGAATTCAAGGGCCACCCGGTGAGCAGGGTGATGGTGGTCCGGCTGGACCACCCAACTCCCTTGACATCGGAACTGTAAC |
|  |  | 36629-36708 | GGAATCCAGGGGCCTCCTGGAGAACAGGGGGATGAGGGTCCATCAGGTCCACCAAATAGCCTGTCTGTTGGAACTGTAAC |
| GMA2-D3 | 63 | 27173-27235 | GTCATTGGCGACATCGTCATGTGGCTTTGGAACACTATTATCAGACCTGCATGGGACGGCATC |
|  |  | 27038-27100 | GTCGTTGGCGATGTCGTCATGTGGCTTTGGAATTCCGTGATGAAGCCAGCTTGGGAAGGCATC |
| GMA2-D4 | 60 | 59494-59553 | AGGATTTGAACCTCGGACCTTCGCCTTATCAGGGCGATGCTCTAACCAACTGAGCTAAAG |
|  |  | 58417-58474 | AGGATTTGAACCTAGGACCTACGGATTAAGAGTCCGCAGCTCTA--CCGCTGAGCTATAG |
| GMA2-D5 | 55 | 13294-13348 | TGTTCGCACTGCAGCTGGAGCTAAGAAGTATGGAGTGCCGATTGGATCTCCAATC |
|  |  | 8379-8433 | TGTTCGTACTGCAGCTGGAGCTAAGAAGTATGGAGTGCCGATTGGATCTCCGATC |
| GMA2-D6 | 37 | 36950-36986 | AAAGGCGACAAAGGCGACCAGGGCAATGTCGGACCAG |
|  |  | 36797-36833 | AAAGGCGACAAAGGCGACCAGGGAAATGCTGGTCCAG |
| GMA2-D7 | 36 | 89007-89042 | AGACTATAGTATCACAGTCTATCCCGAATGTAAACT |
|  |  | 50573-50608 | AGATTACTGTATCATAAACTATCTGGAATGTAAACT |
| GMA2-D8 | 35 | 88988-89022 | GGTGTTCGTTTCGTTGATAAGACTATAGTATCACA |
|  |  | 47883-47915 | GGTATTCGTTCCCGT--TACGACTATAGTATCACA |
| GMA2-D9 | 31 | 88997-89027 | TTCGTTGATAAGACTATAGTATCACAGTCTA |
|  |  | 79337-79367 | TTCGTTGTTGTGTCTACAGTATCACAGTCTA |
| GMA2-D10 | 31 | 97474-97504 | TTCGTTCCCGTTAGTAATACTATATCACACT |
|  |  | 47887-47917 | TTCGTTCCCGTTACGACTATAGTATCACACT |
| GMA2-D11 | 26 | 58732-58757 | CGTACCGCATACGGGAATCGAACCCG |
|  |  | 58075-58100 | CGTACCGGAGACGGGAATCGAACCCG |
| GMA2-D12 | 26 | 81998-82023 | GCGCTCACGGCTGCACCGCCTCGGCG |
|  |  | 81777-81802 | GCGCTCATGACTGCACCGCCTCGGCG |
| GMA2-D13 | 25 | 85019-85043 | GTCGAAGCATCCAGAACTTCTTTGG |
|  |  | 4747-4771 | GTCGAAGTTTCCAGAACTTCTTTGG |
| GMA2-D14 | 25 | 89253-89277 | AACTTCTGCCATGTATGTAGGCACC |
|  |  | 89039-89063 | AACTTCTGCCACGTATGTAGGCGCC |
| GMA2-D15 | 23 | 59640-59662 | AACGGGATTTGAACCCGTGACCT |
|  |  | 59001-59023 | AACGGGATTTGAACCCGTGATCT |
| GMA2-D16 | 21 | 56315-56335 | TCCCAGCGAGAACGCCACCAA |
|  |  | 7553-7573 | TCCCAGCGAGATCGTCACCAA |
| GMA2-D17 | 21 | 36953-36973 | GGCGACAAAGGCGACCAGGGC |
|  |  | 36791-36811 | GGCGACAAAGGCGACAAAGGC |
| GMA2-D18 | 21 | 73530-73550 | GTACGTCCACTGGACTTTCTT |
|  |  | 65254-65274 | GTCCGTACACTGGACTTTCTT |
| GMA2-D19 | 19 | 101384-101402 | GTCTAGACCGAGCTCTTCG |
|  |  | 50424-50442 | GTCTAGAGCGAGCTCTTCG |
| GMA2-D20 | 19 | 96844-96862 | TCGCGAGATCAAGAGCGTC |
|  |  | 54617-54635 | TCGCAAGATCAAGAGCGTC |
| GMA2-D21 | 19 | 60401-60419 | CGGGAATCGAACCCGGGTC |
|  |  | 58086-58104 | CGGGAATCGAACCCGCGTC |
| GMA2-D22 | 19 | 92459-92477 | CGGGCACTTTCTCAGTCAT |
|  |  | 80985-81003 | CGGTCACTTTCTCAGTCAT |
| GMA3-I1 | 35 | 77375-77409 | CTATTTTCAATTTCCTCTAGCTGGCATAGCTAGGC |
|  |  | 77275-77241 | CTATTTTCAATTTCCACTGGCTGGCACAGGTGGGC |
| GMA3-I2 | 29 | 56127-56155 | CCGACGAAACTTTGAGCCTTGGATTCAGA |
|  |  | 15148-15120 | CCAACGGAACGCTGAGCCTTGGATGCAGA |
| GMA3-I3 | 27 | 61835-61861 | CGCTTGCCGCATTCCGAGCAAAACCAA |
|  |  | 8902-8876 | CGCTTGCCGCATTCGGAGCAAGGCCAA |
| GMA3-I4 | 17 | 35821-35837 | AACGGAATACCATCTGA |
|  |  | 6246-6230 | AACGGAATACCATCTGA |
| GMA3-I5 | 17 | 72668-72684 | AAACCGCAGGTCAGAGC |
|  |  | 66060-66044 | AAACCGCAGGTCAGAGC |
| GMA3-I6 | 16 | 54618-54633 | AGAATCGGATGCAGTT |
|  |  | 25123-25108 | AGAATCGGATGCAGTT |
| GMA3-I7 | 16 | 37540-37555 | AATTCACCAGTGATGG |
|  |  | 32009-31994 | AATTCACCAGTGATGG |
| GMA3-I8 | 16 | 67051-67066 | GCGCCACTCAGCGACC |
|  |  | 66457-66442 | GCGCCACTCAGCGACC |
| GMA3-D1 | 102 | 66167-66266 | TGCCAAATTTCAAAAG-TCTCTGACTTGAAAATAATCTGCCAAATTTCAAAAG-TCTCTGACTTGAAAATAATCTGCCAAATTCTGCCAAATTTTGACTTGA |
|  |  | 66061-66158 | TGCCAAATTCCGAACGGTCT-TGACTTGAAA-TAATCTGCCAAATTCCGAACGATCT-TGACTTGAAA-TAATCTGCCAAATCCCGAACGATCTTGACTTGA |
| GMA3-D2 | 73 | 72303-72373 | TCAAATATACCCACCAAAAAATCAAATCATATTGTGACTGGCATATTTGTGTAAACGTCGAG--ACACAAGAT |
|  |  | 72231-72303 | TCAAATATACCCACCAAAAAATCAAATCATATTGTGACTGGCATATTTATGTAAACCTTGTGTAACACAATAT |
| GMA3-D3 | 39 | 32260-32298 | GGCCCAAAGGGCGACAAGGGCGATAAGGGCGATCCTGGA |
|  |  | 31519-31557 | GGCCCGAAGGGCGACAAAGGAGACAAGGGCGATACCGGA |
| GMA3-D4 | 36 | 63522-63556 | CATCGATCTTGTTGACGATATCCGCCG-CCTCTTCG |
|  |  | 29918-29952 | CATCGATCTTGGTGACGAGAT-AGCCGACTTCTTCG |
| GMA3-D5 | 35 | 64516-64550 | ATATCCTTGGCGATTCGCATGATGCTATCGAGTAT |
|  |  | 2095-2128 | ATATCTTTGGCGATTCGATTGATCCTA-CGAGCAT |
| GMA3-D6 | 33 | 32266-32298 | AAGGGCGACAAGGGCGATAAGGGCGATCCTGGA |
|  |  | 32113-32145 | AAGGGGGATAAGGGTGACCAGGGCGATCCTGGA |
| GMA3-D7 | 32 | 16543-16574 | CGCCGATGCAAAGTCGTGCAGCGCAGAATGCG |
|  |  | 10673-10704 | CGTCGACGCTAATTCGTGCAGCGCAGGACGCG |
| GMA3-D8 | 30 | 52532-52561 | CGGTGCCGGTGGTGCGGCGGGTGCCGCCGG |
|  |  | 52445-52474 | CGGTGCCGCAGGAGCGGCGGGTGCTGCCGG |
| GMA3-D9 | 28 | 70585-70612 | TTTTCTCTCCCGTGTTCGTGTGGCTGGT |
|  |  | 69094-69121 | TTTTCTCTCCAGTGATTGTGTGGCTGGT |
| GMA3-D10 | 26 | 29842-29867 | GATGGATCGAGACGCTTGTCGACAAT |
|  |  | 13265-13288 | GATGG-TCGA-ACGCTTGTCGACAAT |
| GMA3-D11 | 24 | 32076-32099 | AAAGGGCGCAAAGGGTGATCAGGG |
|  |  | 31704-31727 | AAAGGGTGACAAGGGTGATCAGGG |
| GMA3-D12 | 23 | 56641-56663 | TCAGTCATTTTTCTCTCCTGTGA |
|  |  | 56217-56239 | TCAGTCATTTTTCTCTCCTGTGA |
| GMA3-D13 | 21 | 69644-69664 | CATGTCATGTACTCCTGTGTG |
|  |  | 68083-68103 | CATGTCATTTACTCCTGTGTG |
| GMA3-D14 | 21 | 34848-34868 | AGCCAACGAAGTATATGGACA |
|  |  | 25612-25632 | AGCCACCGATGTATATGGACA |
| GMA3-D15 | 19 | 31857-31875 | CAAGGGTGATCAGGGAAAT |
|  |  | 31713-31731 | CAAGGGTGATCAGGGAAAT |
| GMA3-D16 | 19 | 56640-56658 | GTCAGTCATTTTTCTCTCC |
|  |  | 40829-40847 | GTCAGTCATTTTTTTCTCC |
| GMA3-D17 | 19 | 65083-65101 | ATTGCGGCGGCGCGGTACA |
|  |  | 41894-41912 | ATTTCGGCGGCGCGGTACA |
| GMA3-D18 | 19 | 48447-48465 | TAATTACGATTCTGGTTTC |
|  |  | 41979-41997 | TAACTACGATTCTGGTTTC |
| GMA4-I1 | 38 | 36221-36258 | GACCCGCAACACCCGCGATGCCGCCAGTGCGGCACGCC |
|  |  | 28933-28898 | GAGCCGCATCACG-GCGA-GCGGCCATTGCGGCACGCC |
| GMA4-I2 | 32 | 19528-19559 | CCGCCGACACCGACCCGCACACCGGGCACCGT |
|  |  | 10963-10933 | CCGCCGACACCGACAGCTTCACC-GGCACCGT |
| GMA4-I3 | 30 | 37813-37840 | CCGCCG--GTTCGTCCACGCCCACGGCCAC |
|  |  | 37338-37309 | CCGCCGTCGTTCTTCCACGCCCACAGCGAC |
| GMA4-I4 | 29 | 15992-16019 | CCGCGTCGA-CCCGCCGGTCGGTGCGTCG |
|  |  | 6135-6107 | CCGCGACGGTCCCGCCGATCGGTGCGTCG |
| GMA4-I5 | 27 | 29132-29157 | TGACCA-GTTCCTCACCGTCGACGACG |
|  |  | 18417-18391 | TGACCACGATCGACACCGTCGACGACG |
| GMA4-I6 | 26 | 31579-31604 | CCGTCAGGTCGGGGCGCGGGTTCGAC |
|  |  | 24349-24324 | CCGGCAGGTCGGGGCGGGTGCTCGAC |
| GMA4-I7 | 25 | 32240-32264 | CGTTCCTGCTCGGAGACCTGCAGAA |
|  |  | 2162-2138 | CGTTCCTCCTCGCGGACCTGCAGAA |
| GMA4-I8 | 25 | 38567-38591 | CGCCTGTACCGCGACCCGTTCGAGT |
|  |  | 19754-19730 | CGCCTGTACCGCCACGCGTTCGTGT |
| GMA4-I9 | 24 | 33319-33342 | TCATCGACGTCGTCCTCGCCGAGG |
|  |  | 31851-31828 | TCATAGGCGTCGTCCTCGCCGAGG |
| GMA4-I10 | 24 | 23437-23460 | CCTCGACCGAGGTCACGATCGTGT |
|  |  | 4053-4030 | CCTCGACAGCGGCCACGATCGTGT |
| GMA4-I11 | 24 | 22266-22289 | GTCCGCCTGGATCGTCAGGATGCG |
|  |  | 12597-12574 | GTTCGCCAGGATCGTCAGGGTGCG |
| GMA4-I12 | 23 | 23639-23661 | ACGCCGTCGCGCTCGATGCGGTC |
|  |  | 1645-1623 | ACGCCGTCAACCTCGATGCGGTC |
| GMA4-I13 | 22 | 25243-25264 | CGAGGATCTCCGACGCGTCCCC |
|  |  | 7566-7545 | CGAGCATCTCCGACGCGTCACC |
| GMA4-I14 | 22 | 29583-29604 | CATCGAGGACGCCCTCGGCGAC |
|  |  | 26684-26663 | CATCGAGGACGCGCTCGCCGAC |
| GMA4-I15 | 21 | 26571-26590 | GACGGCGGCGGCGTG-GGCGC |
|  |  | 8421-8401 | GACGGCGGCGGCGTGCGGCGC |
| GMA4-I16 | 21 | 36326-36346 | ACGACGATGCCCGCCGGAGAC |
|  |  | 21379-21359 | ACGACGATGCCCGACGGCGAC |
| GMA4-I17 | 21 | 43089-43109 | GGGCACCGGTGCCGCCGACGC |
|  |  | 30302-30282 | GGGCACCGGTGCCGACCACGC |
| GMA4-I18 | 20 | 29699-29718 | CCGAACTCGTCGAAGAGTTC |
|  |  | 10294-10275 | CCGAACTCGTCGACGAGTTC |
| GMA4-I19 | 20 | 22172-22191 | GCTCGTCGAGACGATCGGTG |
|  |  | 10584-10565 | GCTCGCCGAGACGATCGGTG |
| GMA4-I20 | 20 | 26665-26684 | CGGCGAGCGCGTCCTCGATG |
|  |  | 17491-17472 | CGGCGAGGGCGTCCTCGATG |
| GMA4-I21 | 20 | 43446-43465 | GATCACCGTCGGCACCACCA |
|  |  | 23682-23663 | GATCACCGTCGGCACCGCCA |
| GMA4-I22 | 20 | 17579-17598 | CCGTCATCGTCGATGCGGTC |
|  |  | 1642-1623 | CCGTCAACCTCGATGCGGTC |
| GMA4-I23 | 20 | 21822-21841 | CGGCGGCGCGGGCAACGTCC |
|  |  | 20844-20825 | CGTCGGCGCGGGCAGCGTCC |
| GMA4-I24 | 19 | 25157-25175 | ATCCATAGTTATCCACAGG |
|  |  | 25104-25086 | ATCCAAAGTTATCCACAGG |
| GMA4-I25 | 18 | 8419-8436 | GTCGAGTTCGGCAACCGA |
|  |  | 1172-1155 | GTCGAGTTCGGCGACCGA |
| GMA4-I26 | 18 | 16785-16802 | GCCGCGACGTGCTCGACG |
|  |  | 7347-7330 | GCCGCGACGTGCTCGTCG |
| GMA4-I27 | 18 | 19706-19723 | CGGTCGGCACCGAGCTCG |
|  |  | 9172-9155 | CGGTCGGCACCGAACTCG |
| GMA4-I28 | 18 | 37485-37502 | CCGCCCTCACCACGCGCA |
|  |  | 18387-18370 | CCGCCCACACCACGCGCA |
| GMA4-I29 | 17 | 27181-27197 | CGGCACGTCCGACAGTC |
|  |  | 14769-14753 | CGGCACGTCCGACAGTC |
| GMA4-I30 | 16 | 38239-38254 | CGACGGGGACGCCGAG |
|  |  | 29638-29623 | CGACGGGGACGCCGAG |
| GMA4-I31 | 15 | 26218-26232 | TGAGTTCAGCGACGG |
|  |  | 17473-17459 | TGAGTTCAGCGACGG |
| GMA4-D1 | 264 | 19327-19584 | CGCACCGTCCAGGTCGCCGCCGGCAGTGCACTCGTCTGCGGTGTGTCCACCGTCGAAA--CGGCGGCCCGACA--GCTGCAGCTCGCAGCGAACACCGGCAGC-CAGGTCCGCCTCGATCTGGTGGTCCTGCGACTGGTGTGGGCGGGCCTCGGTGCGTCGACGGCTGTC-CTCGACATCAAGCAGGGCACGCCGGGCGCGGTGAACCCGCCGACACCGACCCGCACACCGGGCACCGTCTACGAGGCTCCGCTCGCCGTCGTG |
|  |  | 18249-18500 | CGTACCGTGTCGGTCGCCGCCGGTACCGCACAGGTATGCGGCGTG---ACCGT-GAAGTCCGACGCAGCGACGTCGCTGACGTTCGCCGCAAACTCGGGTGGCACA----CGACTCGATGTCGTCGTGCTGCGCGTGGTGTGGGCGGG---CGCGTCGTCGACGG-TGTCGATCGTGGTCAAGCAGGGAACGTCCGGGTCGAGCACCGTCCCGACGCTCACCCGATCGGCAGGCGCGATGTACGAGATGCCGCTCGCGGTCGTG |
| GMA4-D2 | 49 | 44526-44574 | CGTCACCTCCACCGACCTCGTCACCCTCGGAGTCGGTGACGCCCGATGG |
|  |  | 7469-7509 | CGCCACAACCACCGACCTCGTC-------GA-TCGGTGGCGCCCGATGG |
| GMA4-D3 | 35 | 43232-43266 | CCATCGACGGTGTCGCCGTCGTCGCAGGCGACCGC |
|  |  | 36780-36814 | CCATCAACGAGGTCGCCGGCCCCACAGGCGACCGC |
| GMA4-D4 | 35 | 19526-19560 | ACCCGCCGACACCGACCCGCACACCGGGCACCGTC |
|  |  | 17550-17583 | ACTCGCCGACTCCGGCGT-CGCACCGGGCACCGTC |
| GMA4-D5 | 31 | 42196-42223 | CCGCGCTCGTCGACGGACC---CCACCTCCG |
|  |  | 18196-18226 | CCGCACTCGTCGACGGCCCGAACGACCTCCG |
| GMA4-D6 | 27 | 37008-37033 | ACGC-CGCCACCATCAACGACCCCGAC |
|  |  | 35749-35775 | ACGCACGCCACCATCACCGGCCCCAAC |
| GMA4-D7 | 26 | 30366-30391 | CGGAGTTCCCGGTCGTCGTCCGCGAG |
|  |  | 6579-6604 | CGGAGTTCCCGATCGTCGACTTCGAG |
| GMA4-D8 | 26 | 35889-35914 | CGTCGTCCGCACCGAACTCCGCATCG |
|  |  | 17370-17395 | CGTCGTCCGCACCGGCGTCCCCATCG |
| GMA4-D9 | 26 | 26794-26819 | GCGCGTCGACGAGCATGTCGATCGTG |
|  |  | 18388-18413 | GCGCGTCGTCGACGGTGTCGATCGTG |
| GMA4-D10 | 24 | 18611-18634 | CGCCGACGGCGCAACCGGCGCCGA |
|  |  | 17669-17692 | CGCCGACGGCGCACACGGCGACGA |
| GMA4-D11 | 24 | 25940-25963 | CTCGCCGTCGTACGCGTCGTGCCG |
|  |  | 19573-19596 | CTCGCCGTCGTGCGCGTAGCGCCG |
| GMA4-D12 | 23 | 13347-13369 | CCTCGGCCCGATGATGTCGATGG |
|  |  | 869-891 | CCTCGGCCCGATGCTGTCGAAGG |
| GMA4-D13 | 23 | 29583-29605 | CATCGAGGACGCCCTCGGCGACG |
|  |  | 17472-17494 | CATCGAGGACGCCCTCGCCGCCG |
| GMA4-D14 | 23 | 34027-34049 | CGGGGAGGCCGACCGTGGCTGAC |
|  |  | 33568-33590 | CGGGGAGGCCGATCGTGACTGAC |
| GMA4-D15 | 23 | 41042-41064 | CGACCTCGCCAAGACCGAGGACG |
|  |  | 11769-11791 | CGAACTCGCCAAGACCAAGAACG |
| GMA4-D16 | 21 | 42245-42265 | CGTCGACACCATCGCCGACCG |
|  |  | 37078-37098 | CGTCGACACCATCGCCGACCG |
| GMA4-D17 | 21 | 33039-33059 | CTGCACCGCCTCGTCCCCGAG |
|  |  | 32707-32727 | CTGCACCGCCTTGTCCCCGAG |
| GMA4-D18 | 21 | 19488-19508 | TGTCCTCGACATCAAGCAGGG |
|  |  | 12366-12386 | TGTCCTCGACATGACGCAGGG |
| GMA4-D19 | 21 | 42553-42573 | CGACAACCCACTCGGCCGCCA |
|  |  | 36572-36592 | CGACAAAACACTCGGCCGCCA |
| GMA4-D20 | 20 | 38780-38799 | GACGGCGCCGCCCGCGCCCT |
|  |  | 1101-1120 | GACGGCGCCGCGCGTGCCCT |
| GMA4-D21 | 20 | 20912-20931 | GGCGTCGAAGGCGTGGATCG |
|  |  | 3779-3798 | GGCGTCGAAGGTGCGGATCG |
| GMA4-D22 | 20 | 9773-9792 | GTCTGCGAGACGCCGACCGC |
|  |  | 5979-5998 | GTCTCCGCGACGCCGACCGC |
| GMA4-D23 | 20 | 21491-21510 | CGCGACGTCCAGCAGGACGA |
|  |  | 9219-9238 | CGCGACGTCCAGAAGGAGGA |
| GMA4-D24 | 19 | 29700-29718 | CGAACTCGTCGAAGAGTTC |
|  |  | 10274-10292 | CGAACTCGTCGACGAGTTC |
| GMA4-D25 | 19 | 13019-13037 | AGGCACTGACGCCGATCCT |
|  |  | 12695-12713 | AGGCTCTGACGCCGATCCT |
| GMA4-D26 | 19 | 42153-42171 | GCGACACTGCTCCTCACGT |
|  |  | 28283-28301 | GCGACGCTGCTCCTCACGT |
| GMA4-D27 | 18 | 3654-3671 | TCGACGCAGCTGGCAACC |
|  |  | 2120-2137 | TCGACGCAGCTCGCAACC |
| GMA4-D28 | 18 | 25776-25793 | GTCGAACGCGGTCGGCCG |
|  |  | 8080-8097 | GTGGAACGCGGTCGGCCG |
| GMA4-D29 | 17 | 29584-29600 | ATCGAGGACGCCCTCGG |
|  |  | 7788-7804 | ATCGAGGACGCCCTCGG |
| GMA4-D30 | 16 | 31405-31420 | ACCGTGGACCGCCGAG |
|  |  | 2196-2211 | ACCGTGGACCGCCGAG |
| GMA4-D31 | 16 | 17473-17488 | ATCGAGGACGCCCTCG |
|  |  | 7788-7803 | ATCGAGGACGCCCTCG |
| GMA4-D32 | 16 | 36908-36923 | CGCGCCGTCATCGACG |
|  |  | 33312-33327 | CGCGCCGTCATCGACG |
| GMA4-D33 | 15 | 26151-26165 | AGGTCACCGGACTGC |
|  |  | 7212-7226 | AGGTCACCGGACTGC |
| GMA4-D34 | 15 | 29092-29106 | GCGGTGTCGGCATGA |
|  |  | 28838-28852 | GCGGTGTCGGCATGA |
| GMA4-D35 | 15 | 33857-33871 | CATCCGCGCACTCGA |
|  |  | 29007-29021 | CATCCGCGCACTCGA |
| GMA4-D36 | 15 | 33076-33090 | CGCTGCGTGCCGCGC |
|  |  | 29681-29695 | CGCTGCGTGCCGCGC |
| GMA4-D37 | 15 | 38773-38787 | GATCGTCGACGGCGC |
|  |  | 30139-30153 | GATCGTCGACGGCGC |
| GMA4-D38 | 15 | 41035-41049 | ACCTCATCGACCTCG |
|  |  | 30411-30425 | ACCTCATCGACCTCG |
| GMA4-D39 | 15 | 33331-33345 | TCCTCGCCGAGGCGG |
|  |  | 32858-32872 | TCCTCGCCGAGGCGG |
| GMA4-D40 | 15 | 34021-34035 | ACTCGACGGGGAGGC |
|  |  | 33866-33880 | ACTCGACGGGGAGGC |
| GMA5-I1 | 45 | 5827-5871 | GCAGATTCTGTGGCGTGACCAGGTCCCGGCGGGAACGCTCATCGC |
|  |  | 2308-2264 | GCAGATGATGCCGCGTGCCCGTTTGACGGCCGGAACGCTCATCGC |
| GMA5-I2 | 41 | 8067-8107 | CGCCGCAGCGGCCCCGGCCGCCGCCGGGGCCGCTCAGGCGG |
|  |  | 5092-5052 | CGACGCGGCGGCCGACTCTGCGGCCGGGGCCGCTGCGGCGG |
| GMA5-I3 | 32 | 8068-8099 | GCCGCAGCGGCCCCGGCCGCCGCCGGGGCCGC |
|  |  | 5064-5033 | GCCGCTGCGGCGGCGGGTGCCGCCGGGGCGGC |
| GMA5-I4 | 22 | 13867-13888 | CCAGCGGGTCTACGACCTCGGC |
|  |  | 261-240 | CCATCGGGCCGACGACCTCGGC |
| GMA5-I5 | 20 | 14957-14976 | CTCGTCGTCCTCGCCGTCGA |
|  |  | 7922-7903 | CTCGTCGTCCTCGTCGGCGA |
| GMA5-I6 | 19 | 8161-8179 | CTCGGTGCCGTCCTCGCCG |
|  |  | 2578-2560 | CTCGGTGCCGTTCTCGTCG |
| GMA5-I7 | 17 | 4161-4177 | TCGCCTCGGCGGTCGGC |
|  |  | 2805-2789 | TCGCCTCGGCGGGCGGC |
| GMA5-I8 | 17 | 4244-4260 | TGCGCCGCCGTCGCCGG |
|  |  | 3157-3141 | TGCGCCGCCGTCGTCGG |
| GMA5-I9 | 17 | 11509-11525 | CCTCGAACTCGAACGGC |
|  |  | 5477-5461 | CCTCGAACTCGTACGGC |
| GMA5-I10 | 17 | 9761-9777 | GCGACGGCGGCCTCGGT |
|  |  | 8882-8866 | GCGACGGCGGCCACGGT |
| GMA5-I11 | 16 | 11808-11823 | CGACCTCGAACGGCAT |
|  |  | 6601-6586 | CGACCTCGAACGGCAT |
| GMA5-I12 | 15 | 7345-7359 | CGCACCGGCCGCCTG |
|  |  | 5608-5594 | CGCACCGGCCGCCTG |
| GMA5-I13 | 14 | 17300-17313 | TGCCCTCGGTCGTC |
|  |  | 2993-2980 | TGCCCTCGGTCGTC |
| GMA5-D1 | 425 | 9274-9690 | TGGAACGGCATCAAAGCGGCCGTGATGCTCGTCATCGACGGCATCCGCCTCTACATCGAGCTATGGGCGACGATCATCACCGCGATCTGGAACGGCATCAAAGCCGCCGCCGTCGCTGTGTGGAACGGCATACAGATC-GCAGTCCAGGTCGTCGTGACCGTCATCCAGACCATCATCACGACACTCGGC-TCGATCATCACCGCCACATGGAACGGCGTCAAAGCGGTCGCCGAGGCTGTATGGAACGGTATCCAGTCGGTCGTCGACACCGTCGCAGGCGTCATCCGGTCCGCCATCACGACCGCCG--TCGA----CACCGTCATATCGATCTTCAACCGGGTCAAGGGCGTCGCCGAGACGGTATGGGGCGGCATCCAAGGATTCATCGACAACGTCGGCAGCGCCGTCCAGTCCGTCATC |
|  |  | 9154-9570 | TGGAACGGCATCAAAGCCGCCGCAATGTTCGTGCTCAAGCTCATCGTCGCCTACATCACCGTGTGGAAGACGATCATCCTCGCCGTCTGGAACGCCATCAAAGCCGCCGCCGTCGCCGTGTGGAACGGCAT-CAAAGCGGCCGTGATGCTCGTCATCGACGGCATCCGCCTCTACATCGAG-CTATGGGCGACGATCATCACCGCGATCTGGAACGGCATCAAAGCCGCCGCCGTCGCTGTGTGGAACGGCATACAGATCGCAGTCCAGGTCGTCGTGACCGTCATCCAGACCATCATCACGACACTCGGCTCGATCATCACCGCCACATGG------AACGGCGTCAAAGCGGTCGCCGAGGCTGTATGGAACGGTATCCAGTCGGTCGTCGACACCGTCGCAGGCGTCATCCGGTCCGCCATC |
| GMA5-D2 | 46 | 8774-8819 | TCGCATCGCTGGTCGCCTCGCTCGTCTCCGGCCTCGGCCCCGCCCT |
|  |  | 8405-8450 | TCGCATCCAAGGGCGCAGAGTTCGTCACCCGCCTCGGCCCCGGCCT |
| GMA5-D3 | 41 | 9842-9882 | CCGGCCTCGGCCGCCTCGGGTCCGGCCGAGGAGCGATCACC |
|  |  | 8273-8313 | CCGGGTTCGGCCGCCTCGGCGGTGTCCTCGGCGCGATCACC |
| GMA5-D4 | 38 | 8228-8261 | CCGCGTTCAAAGCGGCGAT----GTCGTCGAGCTTCGC |
|  |  | 1688-1725 | CCGCGTTCACGGCCGCGTTCGACGCCGTCGAGCTTCGC |
| GMA5-D5 | 29 | 8059-8087 | GCACTCGCCGCCGCAGCGGCCCCGGCCGC |
|  |  | 5045-5073 | GCACCCGCCGCCGCAGCGGCCCCGGCCGC |
| GMA5-D6 | 28 | 7890-7917 | CCGCCGTCCGCCCTCGCCGACGAGGACG |
|  |  | 1528-1555 | CCGGCGGCCACCGTCGCCGACGAGGCCG |
| GMA5-D7 | 27 | 9357-9383 | GATCTGGAACGGCATCAAAGCCGCCGC |
|  |  | 9150-9176 | GATCTGGAACGGCATCAAAGCCGCCGC |
| GMA5-D8 | 27 | 10737-10763 | TTCGGCGACGACGCCGCGACGTACGTC |
|  |  | 6296-6322 | TTCGGCGACCTCGGCGCGACGTACGTC |
| GMA5-D9 | 27 | 11818-11844 | CGGCATCATCGGCGACGCGATCCCCGC |
|  |  | 8604-8630 | CGGCATCATCGGCGCAGCGATCACCGC |
| GMA5-D10 | 25 | 9239-9263 | TCTGGAACGCCATCAAAGCCGCCGC |
|  |  | 9152-9176 | TCTGGAACGGCATCAAAGCCGCCGC |
| GMA5-D11 | 24 | 12128-12151 | TCGGCCCCGGCCACTACCCGAACA |
|  |  | 8438-8461 | TCGGCCCCGGCCTCGACACGATCA |
| GMA5-D12 | 24 | 16533-16556 | TCGAAGTCGCCGCCGTCGAAGTGT |
|  |  | 9251-9274 | TCAAAGCCGCCGCCGTCGCCGTGT |
| GMA5-D13 | 24 | 16533-16556 | TCGAAGTCGCCGCCGTCGAAGTGT |
|  |  | 9371-9394 | TCAAAGCCGCCGCCGTCGCTGTGT |
| GMA5-D14 | 23 | 9995-10017 | CCCGATGACCGTCAACCCCGCCC |
|  |  | 6663-6684 | CCCGATGACCGTC-ACGCCGCCC |
| GMA5-D15 | 22 | 17513-17534 | GTCACGTTGTGCGTGTGCTCGC |
|  |  | 2455-2476 | GTCACTGTGGGCGTGTGCTCGC |
| GMA5-D16 | 21 | 8663-8683 | CCCTCGGTCCCGTCCTCGCCG |
|  |  | 8159-8179 | CCCTCGGTGCCGTCCTCGCCG |
| GMA5-D17 | 20 | 4522-4541 | CCGCCCCGGCGGCACCCGCC |
|  |  | 5034-5053 | CCGCCCCGGCGGCACCCGCC |
| GMA5-D18 | 20 | 12837-12856 | CACCGACTACGGCAACACGT |
|  |  | 3207-3226 | CACCGTCTACGGCAACACGT |
| GMA5-D19 | 20 | 11942-11961 | CTCAACGGCGGCAAGGGCGC |
|  |  | 3601-3620 | CTCAACGGCGGCACGGCCGC |
| GMA5-D20 | 17 | 7662-7678 | CGATGTGGGCCGCCGCC |
|  |  | 2778-2794 | CGAAGTGGGCCGCCGCC |
| GMA5-D21 | 17 | 9572-9588 | CGACCGCCGTCGACACC |
|  |  | 4341-4357 | CGACGGCCGTCGACACC |
| GMA5-D22 | 15 | 13708-13722 | TCGCCAACAGCGCCG |
|  |  | 5739-5753 | TCGCCAACAGCGCCG |
| GMA5-D23 | 15 | 7744-7758 | CACCGCACCGGCAGG |
|  |  | 6025-6039 | CACCGCACCGGCAGG |
| GMA5-D24 | 14 | 16722-16735 | ACGACCTCGTCGAC |
|  |  | 1067-1080 | ACGACCTCGTCGAC |
| GMA5-D25 | 14 | 9258-9271 | CGCCGCCGTCGCCG |
|  |  | 4246-4259 | CGCCGCCGTCGCCG |
| GMA5-D26 | 14 | 8780-8793 | CGCTGGTCGCCTCG |
|  |  | 5400-5413 | CGCTGGTCGCCTCG |
| GMA5-D27 | 14 | 13545-13558 | TCCTCGACGTGCTC |
|  |  | 7586-7599 | TCCTCGACGTGCTC |
| GMA5-D28 | 14 | 17048-17061 | TACGGCACCGTCCA |
|  |  | 16577-16590 | TACGGCACCGTCCA |
| GMA6-I1 | 23 | 21011-21033 | CCGCCGGACATGAACCTGGCAGA |
|  |  | 14457-14437 | CCGCCGGACATGAAC--GGCAGA |
| GMA6-I2 | 22 | 48594-48615 | AAATGCGTAAGTTCATCGGAGC |
|  |  | 1859-1838 | AAGTGCGTAAGTTCATCGAAGC |
| GMA6-I3 | 16 | 6792-6807 | AGACCGTTCAGAAAAC |
|  |  | 5349-5334 | AGACCGTTCAGAAAAC |
| GMA6-D1 | 85 | 31393-31477 | GCGGGTGAAGAGGGTTATCTGCTTTACGAATCCCTGAACACGTTGTACTTCGCCACCCCGCAGTGGCTCTTCGACAAGCAGCCGA |
|  |  | 30019-30103 | GCCGGGGAAGAAGGCTATATCGCGTACGAGTGCCTGAACACCCTGTACTTCGCGTCCCCTAAGTGGCTCTTCGAGAACCGCCCGA |
| GMA6-D2 | 59 | 27470-27528 | TGGAACATCATCAAGATGGTTGCCTCGGTAGTCTTCAACGCCATCGCCGCAGTCATCCG |
|  |  | 27350-27408 | TGGAACACCATCAAGACTGTCTTTATGGCGGTGTGGAACGCGATCCTCGCAGTCATCCG |
| GMA6-D3 | 57 | 10091-10147 | GTTCGGGACGCTGACTACTGGGGTGCGCCCGTGGGCACTCCGATCGTGGCTGGGATG |
|  |  | 8952-9008 | GTTCGGGACGCTGAGTATTGGGGTATGCCGGTCGGTACTCCGATCACGCCTGGCATG |
| GMA6-D4 | 54 | 50274-50322 | TCGGCATCG-AGCC--TTCGCA-GTTGTTCGGCAAGTTCGTGCCCATGC-CGAA |
|  |  | 24473-24526 | TCGGCATCGCAGCTCGTCCGCAAGTTGTTCGGCAACGACGGGCACATGCTCGAA |
| GMA6-D5 | 41 | 27470-27510 | TGGAACATCATCAAGATGGTTGCCTCGGTAGTCTTCAACGC |
|  |  | 26924-26964 | TGGGACACCATCAAGGCGATCGCGATGGGAGTCTTCAACGC |
| GMA6-D6 | 35 | 51064-51098 | GGAATGGACCGACGAGGACGACGACTCCGACGACG |
|  |  | 21945-21978 | GGAATCGACCGACGAGGACGAC-ACCCCGTCGCCG |
| GMA6-D7 | 31 | 53930-53960 | ATCGGCGCACAGATCAAAGAGCGCCAGGACG |
|  |  | 18388-18418 | ATCGGCGCACAGCTCTACGATCGTCAGGTCG |
| GMA6-D8 | 27 | 67062-67088 | AGAAGCCCCCAAGAAGAAGGTAGGCGC |
|  |  | 54076-54102 | AGAAGCGCTGAAGAAGAAGGTCGGCGC |
| GMA6-D9 | 26 | 61643-61668 | CTCGGCAAGGAGTACGGGGATGACCT |
|  |  | 6749-6774 | CTCAGCAAGTCGTTCGGGGATGACCT |
| GMA6-D10 | 24 | 51136-51159 | CGATGACGACGACGAAGACGACGA |
|  |  | 57590-57613 | CGATGACGACGAGGACGAGGACGA |
| GMA6-D11 | 24 | 51137-51160 | GATGACGACGACGAAGACGACGAG |
|  |  | 58116-58139 | GACGAAGACGCCGAAGACGACGAG |
| GMA6-D12 | 22 | 55185-55206 | GAGCACTACCGCGACGACCTCC |
|  |  | 49811-49832 | GAGCACACCCGCGACGACCTCC |
| GMA6-D13 | 21 | 24267-24287 | GCTCGATCATCGGCAAGGCAC |
|  |  | 11469-11489 | GCTCGATCATCGGCAGCGCAC |
| GMA6-D14 | 21 | 34531-34551 | GGACGATCAACGTATCGATCG |
|  |  | 28497-28516 | GGAC-ATCAACGTATCGATCG |
| GMA6-D15 | 19 | 60365-60383 | GGTTGGGACATCGCGGAAG |
|  |  | 1201-1219 | GGTTGGTACATCGCGGAAG |
| GMA6-D16 | 19 | 41140-41158 | GCGTCGACATCGTGGTCTC |
|  |  | 20527-20545 | GCGTCGACATCGTTGTCTC |
| GMA6-D17 | 19 | 63891-63909 | CCGAAGCGCCGTTCACGAG |
|  |  | 46714-46732 | CCGAAGCGCCGGTCACGAG |
| GMA6-D18 | 18 | 69839-69856 | CAGAGCGCAATGGGCGAG |
|  |  | 17326-17343 | CAGAGCGCAATGGGCGAG |
| GMA6-D19 | 16 | 54208-54223 | GACTACGCCGCGTTCA |
|  |  | 50759-50774 | GACTACGCCGCGTTCA |
| GMA6-D20 | 16 | 61821-61836 | AGCCGGAAGACGAAGA |
|  |  | 58108-58123 | AGCCGGAAGACGAAGA |
| GMA7-I1 | 21 | 71407-71427 | CGACGTCCTCCTCGGCCTGCT |
|  |  | 12052-12032 | CGCCGTCCTTCTCGGCCTGCT |
| GMA7-I2 | 21 | 53040-53060 | CTGCCCCTGAAGTTTGAAGTA |
|  |  | 17729-17709 | CTGCCCCTGAAGCTTGGAGTA |
| GMA7-I3 | 16 | 68898-68913 | CGTTGAGCTTCTCGTA |
|  |  | 7102-7087 | CGTTGAGCTTCTCGTA |
| GMA7-I4 | 16 | 58715-58730 | CCGAGAAGATCTCGCG |
|  |  | 7270-7255 | CCGAGAAGATCTCGCG |
| GMA7-I5 | 16 | 22596-22611 | GACAAGCAGGCCGACG |
|  |  | 16877-16862 | GACAAGCAGGCCGACG |
| GMA7-D1 | 242 | 19701-19940 | GGAAACATTCTCGGATGGCTCGGCAATCTCGGAGGCAAGCTGCTTGAGTGGATGGGGGCTGCGTGGCAATGGCTCGTAGACAATGGCCCAACCATGCTTGCGAAGCTTATTGTGTGGCT--TGCGTCTCTGCCCGCCAAGTTTATCGGCTGGCTCGGCGATATTGGCGGCAAGCTTCTCGAATGGCTCAGGGCGGGTTGGGATTACCTCAAGGACAACTGGCCTATCATTCTCGCCAAGTTT |
|  |  | 19569-19808 | GGAAAGATTCTCGGATGGCTCGGCGACCTCGGCGGCAAGCTGCTCGAGTGGATGGGCGCTGCGTGGAACTGGCTTGTCGAGAATGGGCCGACGATGCTCCTGAATCTCATGACATGGCTCATGGGTATC--GCTGGAAACATTCTCGGATGGCTCGGCAATCTCGGAGGCAAGCTGCTTGAGTGGATGGGGGCTGCGTGGCAATGGCTCGTAGACAATGGCCCAACCATGCTTGCGAAGCTT |
| GMA7-D2 | 50 | 63890-63936 | ACGGTGCCGGTGCTGGGGTCCGAGGCCTCC---ACGGCCCCGTCCACGAC |
|  |  | 47815-47863 | ACGGTGCCGGTGCCGGGG-CGGATGCCCACGGGTCGGAACCGTCCACGAC |
| GMA7-D3 | 48 | 19836-19883 | AAGTTTATCGGCTGGCTCGGCGATATTGGCGGCAAGCTTCTCGAATGG |
|  |  | 19572-19619 | AAGATTCTCGGATGGCTCGGCGACCTCGGCGGCAAGCTGCTCGAGTGG |
| GMA7-D4 | 30 | 66361-66390 | CGTGCCCTACGTAGGACTCGAACCTACGCC |
|  |  | 66016-66045 | CGTGCCCTACGTAGGACTCGAACCTACGCC |
| GMA7-D5 | 28 | 34428-34455 | CCGCCCAGACTGTAGTTGAGGCTTTCGC |
|  |  | 16009-16034 | CCGCCCAGACTGTAGT--CGGCTCTCGC |
| GMA7-D6 | 24 | 67186-67209 | CGTTGTAGTACTAGTCTAACACGT |
|  |  | 66941-66964 | CGTTCTAGTACTAGTCTATCACGT |
| GMA7-D7 | 22 | 51618-51639 | CCAGCTTTCACTGTCCCACGAG |
|  |  | 35317-35338 | CCAGCTTTCACTTTCCCTCGAG |
| GMA7-D8 | 21 | 29185-29205 | CGACGCGATCATCAAGGGTAT |
|  |  | 22607-22627 | CGACGCGATCATCAAGGGCAT |
| GMA7-D9 | 21 | 72861-72881 | CCGGCTCGGGAAAGAATGCGT |
|  |  | 37682-37702 | CCGGCCCTGGAAAGAATGCGT |
| GMA7-D10 | 21 | 62876-62896 | TGATTCTTCCCCTTTCGGTGG |
|  |  | 62356-62376 | TGATTGATCCCCTTTCGGTGG |
| GMA7-D11 | 21 | 67189-67209 | TGTAGTACTAGTCTAACACGT |
|  |  | 67005-67025 | TGTAGTACTAGTCTAGCATGT |
| GMA7-D12 | 19 | 64731-64749 | GTCCCGTCGTTGTCGATCT |
|  |  | 44094-44112 | GTCTCGTCGTTGTCGATCT |
| GMA7-D13 | 17 | 13690-13706 | CGAGGGCGGCAAGGCTG |
|  |  | 6352-6368 | CGAGGGCGGCAAGGCTG |
| GMA7-D14 | 16 | 26359-26374 | ACCTGGACGGCGACCT |
|  |  | 5573-5588 | ACCTGGACGGCGACCT |
| GRU3-I1 | 33 | 14343-14373 | GCCTCGGTCTCGTAGTTGA--AGATCGTTCGTC |
|  |  | 2805-2773 | GCCTCGGTCTCGTCGATGACGAGGTCGTTGGTC |
| GRU3-I2 | 30 | 8990-9019 | TCGTCCTCGCCGTGATCGCCGTCGTCGCCG |
|  |  | 1186-1157 | TCATCGTCGCCGGGGTCGACGTCGTCGCCG |
| GRU3-I3 | 29 | 7668-7696 | CGACGGCACCGAACACAACGACGTCGTCG |
|  |  | 1446-1418 | CGACGGCGGCGACGATCACGACGTCGTCG |
| GRU3-I4 | 27 | 15140-15164 | GGCGA--AGGTTGACCGCGTTGCCGAC |
|  |  | 2722-2696 | GGCGAGCAGGTTGACCGCGTGGCGGAC |
| GRU3-I5 | 20 | 12572-12591 | CGGCGTACCCGACGCGCTCG |
|  |  | 3367-3348 | CGGCGTACCCGCCGCGCACG |
| GRU3-I6 | 19 | 9721-9739 | GCCGGTCTCGGTGTCGTGG |
|  |  | 3160-3142 | GCCGGTCTCGGCGTCGAGG |
| GRU3-I7 | 19 | 11767-11785 | CGGCACCGGCCGCCGCAAC |
|  |  | 8203-8185 | CGACACCGGCCGCCGCTAC |
| GRU3-I8 | 19 | 17410-17428 | GGGCGGTCGTCCGGCGGCG |
|  |  | 9795-9777 | GGACGGGCGTCCGGCGGCG |
| GRU3-I9 | 18 | 15348-15365 | CATGTCGGGTTCCTCTCG |
|  |  | 97-80 | CATGTCGGGTTCCTTTCG |
| GRU3-I10 | 18 | 14787-14804 | GATGACGACCGCCGAGGC |
|  |  | 4228-4211 | GATGCCGACCGCCGAGGC |
| GRU3-I11 | 17 | 17410-17426 | GGGCGGTCGTCCGGCGG |
|  |  | 1317-1301 | GGGCGGTCGTCGGGCGG |
| GRU3-I12 | 16 | 7134-7149 | TCGAGTTCGGCGGCGA |
|  |  | 1597-1582 | TCGAGTTCGGCGGCGA |
| GRU3-I13 | 14 | 8799-8812 | CGACGTCGTCGCCG |
|  |  | 1170-1157 | CGACGTCGTCGCCG |
| GRU3-I14 | 14 | 6104-6117 | GCGATGACCGCGCC |
|  |  | 2541-2528 | GCGATGACCGCGCC |
| GRU3-I15 | 14 | 6336-6349 | CGGGCGGCGTCGAG |
|  |  | 3218-3205 | CGGGCGGCGTCGAG |
| GRU3-I16 | 14 | 12379-12392 | ACAGCCAGGGCGGC |
|  |  | 9227-9214 | ACAGCCAGGGCGGC |
| GRU3-D1 | 158 | 9223-9380 | GCTGTCTGGAATGCGATAAAGGCCGTCGTTCAGTTCGTGATCGATGCGCTGCTCGCCTACATCCAAGTGTGGTCGATGACCATTACCGCGATCTGGAACGCGATCAAGTTCGTAGCGCTCGCGGTCTGGACCGGAATTCAGATCGCCGTGCAGGTCGT |
|  |  | 9103-9260 | GCGGTGTGGAACGCGATCAAGACCGTCGCCGCCGTCGTGATCGCCGCCGTCACCGCCTACGTCAAGGCATGGCAGGCGGTCATTGTCGCCATCTGGAACGCGATAAAGACTGCCGCCCTGGCTGTCTGGAATGCGATAAAGGCCGTCGTTCAGTTCGT |
| GRU3-D2 | 78 | 14151-14228 | CGTCGCGCCCTTCACGCCGCTGTGACCAGCGAAAGCAACTCATCAGCGGGTTCGGGGTTCGAGTCCCTGATGGCGCAC |
|  |  | 14074-14151 | CGTCGCGCCCTTCACGCCGCTGTGACCAGCGAAAGCAACTCATCAGCGGGTTCGGGGTTCGAGTCCCTGATGGCGCAC |
| GRU3-D3 | 45 | 5011-5055 | TGTCGACGCGGTCACCGCCGACGACCTGTCGACCGCCCCGGTAGA |
|  |  | 1622-1666 | TGTCGACGCGGTCGGCGACGCTGACTTGTCGGCGGCCACGGTCGA |
| GRU3-D4 | 30 | 1412-1441 | CGACCGCGACGACGTCGTGATCGTCGCCGC |
|  |  | 412-441 | CGCCCGCGACGACGCCGCGAGCGTCGACGC |
| GRU3-D5 | 30 | 9309-9338 | CGCGATCTGGAACGCGATCAAGTTCGTAGC |
|  |  | 9102-9131 | CGCGGTGTGGAACGCGATCAAGACCGTCGC |
| GRU3-D6 | 29 | 9123-9151 | GACCGTCGCCGCCGTCGTGATCGCCGCCG |
|  |  | 1413-1440 | GACCG-CGACGACGTCGTGATCGTCGCCG |
| GRU3-D7 | 29 | 9125-9153 | CCGTCGCCGCCGTCGTGATCGCCGCCGTC |
|  |  | 8831-8859 | CCGTCATCGCCGACCTCGTCGCCGCCGTC |
| GRU3-D8 | 28 | 9123-9150 | GACCGTCGCCGCCGTCGTGATCGCCGCC |
|  |  | 1430-1457 | GATCGTCGCCGCCGTCGAGAACGCGGCC |
| GRU3-D9 | 27 | 9377-9403 | TCGTCGTCAACATCATCACCGCGATCA |
|  |  | 8672-8698 | TCCTCGGCCAGATCATCACCGCGATCA |
| GRU3-D10 | 26 | 9012-9036 | CGTCGCCGCGATC-GTGCTGCTGGCG |
|  |  | 3969-3994 | CGTCGCCGCGATCGGTGCCGCTGTCG |
| GRU3-D11 | 24 | 9135-9158 | CGTCGTGATCGCCGCCGTCACCGC |
|  |  | 8997-9020 | CGCCGTGATCGCCGTCGTCGCCGC |
| GRU3-D12 | 24 | 9196-9219 | TGGAACGCGATAAAGACTGCCGCC |
|  |  | 9109-9132 | TGGAACGCGATCAAGACCGTCGCC |
| GRU3-D13 | 23 | 11758-11780 | GGCGGCCAACGGCACCGGCCGCC |
|  |  | 7392-7414 | GGCCGCCAAGCGCACCGGCCGCC |
| GRU3-D14 | 22 | 13567-13588 | ATCGCCGCGCACCCTGGCTATC |
|  |  | 1194-1214 | ATCGCCGCGCACCC-GGCCATC |
| GRU3-D15 | 22 | 8381-8400 | AGATG--CAAGGCGTGGGCCGC |
|  |  | 2811-2832 | AGATGGTCAAGGCGTGGGCCGC |
| GRU3-D16 | 22 | 8930-8951 | TCGCCGCCGCCGCAACCGCGCT |
|  |  | 8126-8147 | TCGCCGCCGCCGCGATCCCGCT |
| GRU3-D17 | 22 | 12868-12889 | GGGTGTTCAACGGCAAGACCGC |
|  |  | 8411-8432 | GGGTGTTCAACGGCCTGGCCGC |
| GRU3-D18 | 21 | 8846-8866 | TCGTCGCCGCCGTCGTCAACG |
|  |  | 1432-1452 | TCGTCGCCGCCGTCGAGAACG |
| GRU3-D19 | 20 | 9006-9025 | CGCCGTCGTCGCCGCGATCG |
|  |  | 8799-8818 | CGACGTCGTCGCCGCGATCG |
| GRU3-D20 | 20 | 8799-8818 | CGACGTCGTCGCCGCGATCG |
|  |  | 3963-3982 | CGACACCGTCGCCGCGATCG |
| GRU3-D21 | 19 | 11349-11367 | GCCGACCTCGTCACGATCG |
|  |  | 951-969 | GCCGACCTCGTCGTGATCG |
| GRU3-D22 | 19 | 10778-10796 | CGCCGTCACGACCGCCGAC |
|  |  | 4384-4402 | CGCCGCGACGACCGCCGAC |
| GRU3-D23 | 19 | 13555-13573 | GTCTTCGACGTGATCGCCG |
|  |  | 8992-9010 | GTCCTCGCCGTGATCGCCG |
| GRU3-D24 | 19 | 15716-15734 | TCGCCCGCGCCGCTGAACA |
|  |  | 11365-11383 | TCGCCCGCGCCGCCGAGCA |
| GRU3-D25 | 18 | 8796-8813 | CGCCGACGTCGTCGCCGC |
|  |  | 517-534 | CGCCGAGGTCGTCGCCGC |
| GRU3-D26 | 18 | 8932-8949 | GCCGCCGCCGCAACCGCG |
|  |  | 3704-3721 | GCCGCCGCCGCATCCGCG |
| GRU3-D27 | 18 | 9836-9853 | TCACCGTCGACAACCGCG |
|  |  | 6036-6053 | TCACCGTCGACAACCCCG |
| GRU3-D28 | 17 | 15560-15576 | GCCGGACGCCACCGCCG |
|  |  | 7210-7226 | GCCGGACGCCACCGCCG |
| GRU3-D29 | 17 | 5761-5777 | GTCCTCGGGCACGCTCC |
|  |  | 2683-2699 | GTCCTCGGGCACGGTCC |
| GRU3-D30 | 17 | 12241-12257 | TCGGCCCCATCGCCAAC |
|  |  | 8603-8619 | TCGGCCCCATCGTCAAC |
| GRU3-D31 | 15 | 8120-8134 | CGTCGATCGCCGCCG |
|  |  | 1576-1590 | CGTCGATCGCCGCCG |
| GRU3-D32 | 15 | 11140-11154 | ACGCCGCCGCCCTCG |
|  |  | 2934-2948 | ACGCCGCCGCCCTCG |
| GRU3-D33 | 15 | 6151-6165 | CGCCGGGTGGCTCGG |
|  |  | 5332-5346 | CGCCGGGTGGCTCGG |
| GRU3-D34 | 15 | 9126-9140 | CGTCGCCGCCGTCGT |
|  |  | 8847-8861 | CGTCGCCGCCGTCGT |
| GRU3-D35 | 14 | 15564-15577 | GACGCCACCGCCGC |
|  |  | 209-222 | GACGCCACCGCCGC |
| GRU3-D36 | 14 | 11990-12003 | GGCTCACTCGCACT |
|  |  | 1128-1141 | GGCTCACTCGCACT |
| GRU3-D37 | 14 | 14907-14920 | CTTCGACCGCGACG |
|  |  | 1409-1422 | CTTCGACCGCGACG |
| GRU3-D38 | 14 | 6814-6827 | CCCACCGGTCGCCC |
|  |  | 1880-1893 | CCCACCGGTCGCCC |
| GRU3-D39 | 14 | 17356-17369 | AGACCGGCGGCCGA |
|  |  | 3941-3954 | AGACCGGCGGCCGA |
| GRU3-D40 | 14 | 10046-10059 | CGACGACCTGTCGA |
|  |  | 5029-5042 | CGACGACCTGTCGA |
| GRU3-D41 | 14 | 6519-6532 | CCAACCCGGCCGAC |
|  |  | 5793-5806 | CCAACCCGGCCGAC |
| GRU3-D42 | 14 | 9140-9153 | TGATCGCCGCCGTC |
|  |  | 7921-7934 | TGATCGCCGCCGTC |
| GTE6-I1 | 56 | 39696-39751 | ACCGCCGGACGGCGAGCAAGTCGAGCAGCTGATCGCGCTCGCGCAGGATCTCGTCG |
|  |  | 7842-7795 | ACCGC-GGTCGCCG-GCAGGTCGA-CACC-GATCGCGC----GCAGGATCTCGACG |
| GTE6-I2 | 55 | 10343-10394 | GCTCGCCGAACTGCAGGGCCTCGACCTCGAAC-AGCTGCGCCG--GCGCGAGTTC |
|  |  | 9690-9639 | GCTCGCCGAACTGCA---CCTCGATCTCATCCGAGTCACGCCGGCGCGCGAGTTC |
| GTE6-I3 | 51 | 48160-48208 | CGCGGCCGCGATCGCCGAGCACG-CAGCC-GGCCGCGACCAGCGCAGCAAC |
|  |  | 37923-37875 | CGCGGCCGCGATCG--TAGGACGCCTGCCGGGCGGCGATCAGCTTTGCAAC |
| GTE6-I4 | 42 | 53601-53640 | CCGGCCGG--CGTCGCCGCGGCCGGCAGCATTGCCTACACCG |
|  |  | 9099-9058 | CCTGCCGGATCTTCGCCGCGGCCAGCTGCCCGGCCGACACCG |
| GTE6-I5 | 38 | 38196-38233 | CGCCGAGGCTCGCGCCGCCGGCGAACTGCTGTACCTGC |
|  |  | 23824-23790 | CGCCGAGGATCGGGCCG---GCGAACTGCACCACCTGC |
| GTE6-I6 | 37 | 54932-54968 | CGGCGGTGGAAACACGAGCAGGCGCGCAGCGGTCCCG |
|  |  | 31987-31951 | CGGCGGCGTGATCATGAGGATGCGCGCAGCGGTGCCG |
| GTE6-I7 | 35 | 52080-52114 | CGCCCGGATCTGAACGACGACGACGAGACGGGCAA |
|  |  | 43835-43804 | CGCCCGGATCT---CGTCGACGACGCGGCGCGCAA |
| GTE6-I8 | 35 | 50812-50846 | GCGCGCGCTGATCGCCGAGATCGAGGTCGACGACG |
|  |  | 33984-33950 | GCGCAGGTTGATCGCCGAGATCCGGCCCGACGCCG |
| GTE6-I9 | 33 | 54279-54311 | TCGACCCGCTCGTCGTCGACAAGCACCTCGACC |
|  |  | 12264-12232 | TCGACCGGCTCGTCGTCGAACTGCTCGTCGACC |
| GTE6-I10 | 31 | 42277-42307 | CTCGCCGGGCACCTCGAAAACGGCGGCCGGC |
|  |  | 35880-35850 | CTCGCCGTGCAGCCAGAACCCGGCGGCCGGC |
| GTE6-I11 | 28 | 3940-3967 | TCGACCCGGCCGAAGAGAGCGAGTAGCC |
|  |  | 1331-1304 | TCGACCCGGCCGTCGCGCGCGAGTAGCC |
| GTE6-I12 | 28 | 36470-36497 | CCGGCATCGCCGGCACGCTCGCGCTCGT |
|  |  | 20125-20098 | CCGTCATCGCCGGCAGGCTCGCTATCGT |
| GTE6-I13 | 28 | 33924-33951 | CGCCACGATCGCGGCCACCGGCTCGCCG |
|  |  | 8453-8428 | CGCCCCGATCGCGGCCA--GGGTCGCCG |
| GTE6-I14 | 28 | 50817-50844 | CGCTGATCGCCGAGATCGAGGTCGACGA |
|  |  | 30635-30609 | CGGTGATCGCCGAGATC-ACGTCGCCGA |
| GTE6-I15 | 28 | 52742-52769 | TCGTCGGCGCGATCGACGACAACCCGCC |
|  |  | 9406-9379 | TCGTCGGCGCGGTCGGCGTCATCCGGCC |
| GTE6-I16 | 28 | 37149-37176 | GCTGCCCGGCGTGTTCGGAGCGGCTGGC |
|  |  | 26700-26673 | GCTGCCCGGCGTGGTCGTGGTGGCCGGC |
| GTE6-I17 | 27 | 23533-23559 | TTCTTCCAGTCGACGGCGGCCGCGGTC |
|  |  | 11688-11662 | TTCTGCGCGTCGTCGGCGGCCGCGGTC |
| GTE6-I18 | 26 | 22386-22411 | TTCGGCGGTGTCGGTGACGATGACCG |
|  |  | 2363-2338 | TTCGGCGACGTTGGTGACGATGACCG |
| GTE6-I19 | 26 | 49588-49613 | GCCTGAACGGCGAGTTCGCCGCGCTC |
|  |  | 288-263 | GCCTGGTCGGCGAGTTCGGCGCGTTC |
| GTE6-I20 | 26 | 21895-21920 | GCTGATCGGCCGGCAACTCGTCGGCG |
|  |  | 9870-9845 | GCGGATCGGCCGGCACCACGTCGCCG |
| GTE6-I21 | 25 | 4751-4772 | CGGCACGCCGGCCG---AGGCGATC |
|  |  | 3159-3135 | CGGCACGCCGGCCGGATAGGCGATC |
| GTE6-I22 | 25 | 50866-50890 | CACGCTCGGCGCGACCGGCCGCCGG |
|  |  | 19591-19567 | CACGCTCGGCGCATCCTGCGGCCGG |
| GTE6-I23 | 24 | 36165-36188 | CGTCGCCGACCTCGCCGAGCGCGC |
|  |  | 10724-10702 | CGTCGCCCAC-TCGCCGAGCGCGC |
| GTE6-I24 | 24 | 55094-55117 | ACCACGACGACGAGCAGATCGTCG |
|  |  | 9856-9833 | ACCACGTCGCCGGGCAGATCGTCG |
| GTE6-I25 | 24 | 40693-40716 | CGAGGCACCCGGCGTCGACGACGA |
|  |  | 26226-26203 | CGAGGTACCCGGCGTCGTCGTCGA |
| GTE6-I26 | 24 | 30865-30885 | CGGTGGT---GCGATCGCCGACGT |
|  |  | 3813-3790 | CGGTGGTCACGCGATCGCCGACGT |
| GTE6-I27 | 24 | 23604-23627 | CGGTACCGTCGCGCCGGCGATCGC |
|  |  | 22696-22675 | CGGTAGCG--GCGCCGGCGATCGC |
| GTE6-I28 | 23 | 34643-34665 | TGTTTCAGCAGCAGGTTCGGCAG |
|  |  | 683-661 | TGTTTCAGCAGCAGTTTCTGCAG |
| GTE6-I29 | 23 | 22748-22770 | GGTCGACGCTGGTCGCCGGCGCG |
|  |  | 16689-16667 | GGTCGAGGCTGGTCGCCGTCGCG |
| GTE6-I30 | 23 | 35104-35126 | GAGGTCGACGACGAGCACACGGT |
|  |  | 4085-4063 | GAGGTCGACGACGCGCGCAGGGT |
| GTE6-I31 | 23 | 13910-13932 | GCAGTCGGCGAGATCCGGCCCGA |
|  |  | 9398-9376 | GCGGTCGGCGTCATCCGGCCCGA |
| GTE6-I32 | 23 | 52447-52469 | CTCGTCGACGACGCCGGCCGCAA |
|  |  | 43826-43804 | CTCGTCGACGACGCGGCGCGCAA |
| GTE6-I33 | 22 | 31267-31288 | CACCGGCTCGACGGGCTGGTCG |
|  |  | 12271-12250 | CACCGGCTCGACCGGCTCGTCG |
| GTE6-I34 | 22 | 49598-49619 | CGAGTTCGCCGCGCTCGTCGCC |
|  |  | 13793-13772 | CGAGTTCGCCGCCCTCGGCGCC |
| GTE6-I35 | 22 | 54005-54026 | CCGACGCGCGGCGTCGAGGTGC |
|  |  | 43980-43960 | CCGA-GCGCGGCGTCGTGGTGC |
| GTE6-I36 | 21 | 33953-33973 | CGTCGGGCCGGATCTCGGCGA |
|  |  | 13934-13914 | CGTCGGGCCGGATCTCGCCGA |
| GTE6-I37 | 21 | 52209-52229 | GGCCGCCCGAGCGCATCGACC |
|  |  | 1105-1085 | GGCCACGCGAGCGCATCGACC |
| GTE6-I38 | 21 | 9892-9912 | GCTCGCAGGATCTCGACGCCG |
|  |  | 7812-7792 | GCGCGCAGGATCTCGACGGCG |
| GTE6-I39 | 21 | 37751-37771 | CGCACTCGGCGACCTGCTCGA |
|  |  | 9432-9412 | CGCGCTCGGCGACCTGCACGA |
| GTE6-I40 | 21 | 43241-43261 | CTGCCGAGCACCCGTCGGCCG |
|  |  | 10896-10876 | CTACCGAGCACCCGCCGGCCG |
| GTE6-I41 | 21 | 53608-53628 | GCGTCGCCGCGGCCGGCAGCA |
|  |  | 12314-12294 | GCGGCGCCTCGGCCGGCAGCA |
| GTE6-I42 | 21 | 31980-32000 | CGCCGCCGGCGACCGAGCCGG |
|  |  | 18696-18676 | CGCCGCCGGCGAGCGTGCCGG |
| GTE6-I43 | 21 | 39008-39028 | CGCATCGACCGCGACGAGGAT |
|  |  | 24138-24118 | CGCACCGACGGCGACGAGGAT |
| GTE6-I44 | 21 | 48482-48502 | CGTACTCGTCGCCGTCGACGA |
|  |  | 33457-33437 | CGGACACGTCGCCGTCGACGA |
| GTE6-I45 | 20 | 48187-48206 | CGGCCGCGACCAGCGCAGCA |
|  |  | 3085-3066 | CGGCCGCGGCCAGCGCAGCA |
| GTE6-I46 | 20 | 16879-16898 | AGGCGCCGGCGGCCGGGCAG |
|  |  | 4588-4569 | AGACGCCGGCGGCCGGGCAG |
| GTE6-I47 | 20 | 20958-20977 | ACCCGCAGCAGCGCACGAAC |
|  |  | 7056-7037 | ACCGGCAGCAGCGCACGAAC |
| GTE6-I48 | 20 | 26610-26629 | GACCTCGCAGGGCACGGTGC |
|  |  | 241-223 | GACCT-GCAGGGCACGGTGC |
| GTE6-I49 | 20 | 50827-50846 | CGAGATCGAGGTCGACGACG |
|  |  | 500-481 | CGCGATCGAGGTCGTCGACG |
| GTE6-I50 | 20 | 45151-45170 | TCGCCGCCCGACTCGTCGAC |
|  |  | 1661-1642 | TCGCCGCCCGACGGGTCGAC |
| GTE6-I51 | 20 | 44774-44793 | CGGCGGCCGCGTCCTGCGCA |
|  |  | 3088-3069 | CGGCGGCCGCGGCCAGCGCA |
| GTE6-I52 | 20 | 30112-30131 | GCGGCGAGGCTGTGCGCCGG |
|  |  | 4852-4833 | GCGGCGAGGCTGCGGGCCGG |
| GTE6-I53 | 20 | 28355-28374 | TCGACGAGTGCAACGACGAC |
|  |  | 6899-6880 | TCGACGAGTGCACCGACCAC |
| GTE6-I54 | 20 | 31465-31484 | CGCGCAGCTGCAGAACGCCG |
|  |  | 21573-21554 | CGCGCAGCTGCAGTTCGCCG |
| GTE6-I55 | 20 | 47451-47470 | GCTCGACCCGAACGTGCCGC |
|  |  | 27209-27190 | GCTCGACCCGAGCGTGACGC |
| GTE6-I56 | 19 | 26481-26499 | GATCGCGGCGAAGATCTTC |
|  |  | 24471-24453 | GATCGCGGCGAAGATCTTC |
| GTE6-I57 | 18 | 38745-38762 | TCGGCGTCGATCACTACG |
|  |  | 812-795 | TCGGCGTCGATCACTTCG |
| GTE6-I58 | 18 | 39562-39579 | ATTCCAGTCGCGCAGCGT |
|  |  | 1530-1513 | ATTCCAGTCGCGCAGGGT |
| GTE6-I59 | 18 | 33455-33472 | CCGGGTCGGCCGGCATCG |
|  |  | 9501-9484 | CCGGCTCGGCCGGCATCG |
| GTE6-I60 | 18 | 43612-43629 | CCGACGTGCAGATCGCCG |
|  |  | 11286-11269 | CCGACGGGCAGATCGCCG |
| GTE6-I61 | 18 | 54446-54463 | CTCGCCGAGGTCGACGAC |
|  |  | 13227-13210 | CTCGCCGAGGTGGACGAC |
| GTE6-I62 | 18 | 51371-51388 | AGTGCGACCAGTGCGGCG |
|  |  | 17068-17051 | AGTGCGACCAGCGCGGCG |
| GTE6-I63 | 18 | 32562-32579 | CGCCGACACGGTGGCCGC |
|  |  | 19498-19481 | CGCCGCCACGGTGGCCGC |
| GTE6-I64 | 18 | 39735-39752 | CGCGCAGGATCTCGTCGA |
|  |  | 30842-30825 | CGCGCAGGATCGCGTCGA |
| GTE6-I65 | 18 | 55101-55118 | CGACGAGCAGATCGTCGA |
|  |  | 50739-50722 | CGCCGAGCAGATCGTCGA |
| GTE6-I66 | 18 | 55089-55106 | CGATGACCACGACGACGA |
|  |  | 52579-52562 | CGGTGACCACGACGACGA |
| GTE6-I67 | 17 | 14475-14491 | TCGAACTGCTCGTCGAC |
|  |  | 12249-12233 | TCGAACTGCTCGTCGAC |
| GTE6-I68 | 17 | 53593-53609 | CCGGCGCACCGGCCGGC |
|  |  | 25837-25821 | CCGGCGCACCGGCCGGC |
| GTE6-I69 | 16 | 22111-22126 | TTCGACCGCATGGCCG |
|  |  | 8087-8072 | TTCGACCGCATGGCCG |
| GTE6-I70 | 16 | 34966-34981 | GGCAGCATCGGCACCG |
|  |  | 19815-19800 | GGCAGCATCGGCACCG |
| GTE6-I71 | 16 | 34383-34398 | ACCCACCGATCGAGGG |
|  |  | 21676-21661 | ACCCACCGATCGAGGG |
| GTE6-I72 | 15 | 55366-55380 | GACGATCGCCTCGCG |
|  |  | 1269-1255 | GACGATCGCCTCGCG |
| GTE6-I73 | 15 | 49958-49972 | ATCGGGCACAGCGAG |
|  |  | 1922-1908 | ATCGGGCACAGCGAG |
| GTE6-I74 | 15 | 4571-4585 | GCCCGGCCGCCGGCG |
|  |  | 2955-2941 | GCCCGGCCGCCGGCG |
| GTE6-I75 | 15 | 54195-54209 | TGAACGCGCCGTTCG |
|  |  | 8712-8698 | TGAACGCGCCGTTCG |
| GTE6-I76 | 15 | 33741-33755 | CTTCAACCGGTCCGA |
|  |  | 8756-8742 | CTTCAACCGGTCCGA |
| GTE6-I77 | 15 | 23549-23563 | CGGCCGCGGTCGGCA |
|  |  | 10814-10800 | CGGCCGCGGTCGGCA |
| GTE6-I78 | 15 | 15215-15229 | CTCGATCTGCCCGAG |
|  |  | 12751-12737 | CTCGATCTGCCCGAG |
| GTE6-I79 | 15 | 33386-33400 | CGGATCTCGCCGACT |
|  |  | 13926-13912 | CGGATCTCGCCGACT |
| GTE6-I80 | 15 | 32443-32457 | GCCGCCGGCGCCGCA |
|  |  | 18091-18077 | GCCGCCGGCGCCGCA |
| GTE6-I81 | 15 | 24337-24351 | GCCACGTTCACGGCG |
|  |  | 23369-23355 | GCCACGTTCACGGCG |
| GTE6-I82 | 15 | 41200-41214 | CGGCGCCGACGGCAC |
|  |  | 24685-24671 | CGGCGCCGACGGCAC |
| GTE6-I83 | 15 | 44737-44751 | CGATCGCCTGCCCGA |
|  |  | 29185-29171 | CGATCGCCTGCCCGA |
| GTE6-I84 | 15 | 52549-52563 | GCCGCCGGGCACGTC |
|  |  | 29846-29832 | GCCGCCGGGCACGTC |
| GTE6-I85 | 15 | 43357-43371 | GCGAACAGATCGCCG |
|  |  | 30786-30772 | GCGAACAGATCGCCG |
| GTE6-I86 | 15 | 56753-56767 | CCGACGATCGAGTCG |
|  |  | 39192-39178 | CCGACGATCGAGTCG |
| GTE6-I87 | 15 | 54669-54683 | CGTCGACCTCGGCGA |
|  |  | 54461-54447 | CGTCGACCTCGGCGA |
| GTE6-D1 | 110 | 19997-20093 | CGATCCGGCCGACGGCGAGTTCGACACCTCGAC-CGC---ACCG---ACGACCCCG----CCGG--CGCAGGCGCCGAGCGCCGGCAGCGACGACGCCGGCGAGGCCGAC |
|  |  | 2799-2905 | CGATC--GCCGACGGCGACCACGACACCGCGGCACGCGCTACCGCTCGCGACGCCGAGACCCGGCTCGCAGCCGCTCA-CGCCGGCATCCTCGCGATCGTCGAGGCCGAC |
| GTE6-D2 | 101 | 24673-24771 | GCCGTCGGCGCCGCGGTTATGTGGTTCTGGAACACCATTATCGCGCCGGCGTTCGCCGCGATCGGCGCGATCATCTCGGC--GTGGTGGACCGGCGTGCAG |
|  |  | 24541-24639 | GCCGTCGGCGCCGTGTTTACCTGGCTGTGGCAGACGATCATCGTGCCGGCGTTCACCGCGATACGGGCCGT--TTTCGACCTGTGGTGGGCCGGCGTGCAG |
| GTE6-D3 | 92 | 13584-13675 | ACCGGCTCGAAGAGGACCAGGTACCGGACACCGGCGGCGAGGGCGGCAAGCTCGTCGAGTTCGAGAACGGCGTCGCGAAGTACGACGACGGC |
|  |  | 13326-13417 | ACCCGATGGAAGAGGACGAGTCGCCCGACAAGGGTGCGCTCGGCGGCAAGCTGATCAGCTACGGCGACGGCCGCGCCGACTACGACGACGGC |
| GTE6-D4 | 85 | 5677-5761 | GCGGTGAAGAAAGCCGCCGAGTACATGGATTACTCGTCGTTCTCGCAGCAGGGCCTCGTCGATCAACTCGTGTTCGAGGGATTCA |
|  |  | 5536-5620 | GCCGTGAGCAAGGCCAAGGATTACCTCGAATACTCGGCGTTCTCCCGTTCGGGCCTGATCAAGCAACTCGCGTTCGAGGGATTCA |
| GTE6-D5 | 75 | 13772-13846 | GGCGCCGAGGGCGGCGAACTCGTGTCGTTCGGTGACGGCGTGGCCGTGTATGACGACGGCACCGAGACCGACGGC |
|  |  | 13616-13690 | GGCGGCGAGGGCGGCAAGCTCGTCGAGTTCGAGAACGGCGTCGCGAAGTACGACGACGGCACCGAAACGAACGGC |
| GTE6-D6 | 60 | 44292-44351 | CGCCCGCGCCAACACGCCGCCGTGGACGATCACGGCGTGGCTCGTCGTCGACCCGTCCGG |
|  |  | 1598-1655 | CGACCGCGCCGGCAC-CCGACGAGTTCCGTCGCGTCGTGA-TCGGCGTCGACCCGTCGGG |
| GTE6-D7 | 55 | 55032-55085 | CCGTGCACC-CCGAGGACGACTACACGATGACAGGAGTACCCACCGAATGACCGA |
|  |  | 49725-49779 | CCGTGCATCACCGAGCACGGCACCACCACCACGGAAGGATCGACCGAATGACCGA |
| GTE6-D8 | 54 | 17792-17840 | CTCGTGTCGATCGACGTGA--GCG---CCGAGTACGAGGACGGCACCGAGAACG |
|  |  | 13790-13841 | CTCGTGTCGTTCG--GTGACGGCGTGGCCGTGTATGACGACGGCACCGAGACCG |
| GTE6-D9 | 54 | 47895-47948 | CGCCGAACGTCACCCTGCACGGCGCCGACTGGGAGCCGATCAACCCGCTCGACC |
|  |  | 29827-29876 | CGCCGGACGTG-CCCGGC--GGCGGCGACCTGCCGCCGATCAACCTGC-CGACC |
| GTE6-D10 | 52 | 10011-10062 | GCCGAGGGCCGGCTGAACGAGGCGACGCAGGCTGCCGAGGTCGACGACGACG |
|  |  | 20057-20100 | GCCGAGCGCCGGCAG--CGA--CGACGCCGGC----GAGGCCGACGACGACG |
| GTE6-D11 | 52 | 46606-46656 | CCGGCAAGGGCGCCGGCGCCGGCACCGATAAGACCGGCGTC-GCCGCCGGCA |
|  |  | 3374-3425 | CCGGCGTGAGCGCGGTCGCCGGCACCGTGGCGATCATCGTCGGCCGCCGGCA |
| GTE6-D12 | 50 | 47664-47712 | CGACGCCGTGCACGAGGTGATCGACCG-GTTCGGGCAGCAGATCGCCGAC |
|  |  | 9905-9953 | CGACGCCGTGCAGGCCGAGTTCGACCGTGTGCAGG-ATGAGTTCGCCGAC |
| GTE6-D13 | 49 | 51782-51830 | CATCGACCGCGACGACGAGCACCCGGACCTCGACCCCGACGTGCCGGTC |
|  |  | 35100-35142 | CATCGAGGTCGACGACGAGCACACGGTGCT-GA-----ACGTGCCGGTC |
| GTE6-D14 | 48 | 35888-35935 | AGGCGTACGCCGCGCAGAAGTGGGAGCAGGGCGCGCTCGGCTGGCCGA |
|  |  | 35714-35761 | AGGCGTACGCCGCGCGCAACTGGGAGGCCGGCCCGCTCGGGTTCCCGA |
| GTE6-D15 | 47 | 45972-46016 | AGCCGAGGCGGATCTCGCCGCAGT--CGATCAGGCGCTCGCCGAGCT |
|  |  | 10310-10354 | AGCCGAGGCCGAAG--GCATCAGTTACGAGCAGGCGCTCGCCGAACT |
| GTE6-D16 | 46 | 44103-44147 | CGACGCACGCGGCAACCTGACCGTGC-CGACGCCGGCCGAACTCGA |
|  |  | 34536-34579 | CGACGCAGACGGCAACCTGACGTTGCGCAACCTCG--CGAACTCGA |
| GTE6-D17 | 45 | 15282-15325 | CGATCTACGCGGGCGGCATGGC-CGGCCGTGTGATGACCGAGGCG |
|  |  | 1826-1869 | CGAACTACGGCGGCGGCATGGCTCGGCAG-CTGATCACGCAGGCG |
| GTE6-D18 | 44 | 40882-40922 | CGACGC---ACCAGCCGACCCCGCAGACCTCACCGCCGACGAGG |
|  |  | 7926-7967 | CGACGCTCGACAAGCCGACCCCGCAGA--TCATCCCCGGAGAGG |
| GTE6-D19 | 41 | 50808-50848 | GCCGGCGCGCGCTGATCGCCGAGATCGAGGTCGACGACGAC |
|  |  | 9689-9728 | GCCGGAACACGCCGA-CGACGTGCTCGAAGTCGACGACGAC |
| GTE6-D20 | 40 | 50765-50804 | GCCGACGGCGTGCAGTTCCGGTTCGACATGCCGGTGACCG |
|  |  | 542-575 | GCCGACGGCGTGGA--TC----TCGACCTGCCGGTGACCG |
| GTE6-D21 | 38 | 53591-53625 | CACCGGCGCACCGGCCGG---CGTCGCCGCGGCCGGCA |
|  |  | 5376-5413 | CACCCGCGCAATCGCCGGACTCGTCGCCGCGGCCGGCA |
| GTE6-D22 | 38 | 54699-54736 | CGTGTTCGTCGTCGACGGCAACACCCCGCCGAAGGCGC |
|  |  | 39217-39254 | CGTGAACCTCGTCGACGGCGAGACCGCGGCCAAGGCGC |
| GTE6-D23 | 38 | 48464-48501 | ACTCGGCTACCCCGCCGGCGTACTCGTCGCCGTCGACG |
|  |  | 5394-5431 | ACTCGTCGCCGCGGCCGGCATCGTCGTCGCCGTCGCCG |
| GTE6-D24 | 37 | 30472-30505 | GCAGAACGTCGACCCCGACG---CGCTCGGCGCCGAG |
|  |  | 26696-26732 | GCAGCACGACGACCCCGACGACACCCTCGACGCCGAG |
| GTE6-D25 | 36 | 9328-9363 | TCGACGAGCAGGGCGGTAAGGGGCCGGTCGAGCCGG |
|  |  | 12234-12269 | TCGACGAGCAGTTCGACGACGAGCCGGTCGAGCCGG |
| GTE6-D26 | 36 | 43690-43722 | CGACCGTCGACGA---CAGTCGCGAGAACGCCGCAC |
|  |  | 477-512 | CGACCGTCGACGACCTCGATCGCGAGATCGCCGAAC |
| GTE6-D27 | 36 | 54166-54201 | GTGGGCCGCCGGCTACCTCGTGGCCGTGATGAACGC |
|  |  | 52545-52580 | GTGGGCCGCCGGGCACGTCGTCGTCGTGGTCACCGC |
| GTE6-D28 | 36 | 22768-22801 | GCGGGTGCCGGCATCGC--CGCGATCGGTGCGCTCG |
|  |  | 3662-3696 | GCCGGTGCCGGCATCGCGGGGCGA-CGTTGCCCTCG |
| GTE6-D29 | 36 | 19571-19603 | CCGCAGGATG-CGCCGA--GCGTGGTCGCGCTCGAC |
|  |  | 18329-18364 | CCGCCGGCTGACACCGACGGCGCGGTCGCGCTCGAC |
| GTE6-D30 | 35 | 36462-36496 | GCAGGCCGCCGGCATCGCCGGCACGCTCGCGCTCG |
|  |  | 31807-31841 | GCTGGCCGCCGGCAGCGCGGTCACGCTCGTGTTCG |
| GTE6-D31 | 35 | 18902-18936 | TCGGTGGCTGCGCCGCGTCGACGGGCTCGCGTGGC |
|  |  | 5161-5193 | TCGGCGACGGCGCC-CG-CGACGGGCTCGGGTGGC |
| GTE6-D32 | 35 | 52033-52067 | CTGCCGGCGACGGCGTGTTCCCCCTCGGCTCGATC |
|  |  | 23438-23469 | CTGCCGGCGACGGCAATTT---CCTCGGCTCGCTC |
| GTE6-D33 | 35 | 11698-11732 | GATCGCGCTCACTGCCCGCGACGTGAAGGCGCGCG |
|  |  | 4640-4674 | GACCGGGCTGACCGCGAGCAACGTGAAGGCGCTCG |
| GTE6-D34 | 35 | 20832-20866 | CGACCGACACCGGCGAGGATCGCGAGGTCGAGCGC |
|  |  | 14649-14683 | CGACCGACACCGGTGACCCTGCCAAGGTCGAGGGC |
| GTE6-D35 | 35 | 53694-53728 | ACCCCGCTCGGCGTCGTCATCTACAACACCGGCAA |
|  |  | 49913-49947 | ACCCCGCTCGGGTTCGACGTGCAGATCACCGGCAA |
| GTE6-D36 | 34 | 48297-48330 | AGTAGCATCACGCCGTACCCACCAAGTACCGACC |
|  |  | 46167-46197 | AGTAGCATCACCGTGTACCCACCA---ACCGACC |
| GTE6-D37 | 34 | 33945-33976 | CTCGCCGGCGTCGG-GCCG-GATCTCGGCGATCA |
|  |  | 30599-30632 | CTCGACGGCATCGGCGACGTGATCTCGGCGATCA |
| GTE6-D38 | 34 | 52731-52764 | CGAGTTCGAGATCGTCGGCGCGATCGACGACAAC |
|  |  | 13639-13672 | CGAGTTCGAGAACGGCGTCGCGAAGTACGACGAC |
| GTE6-D39 | 34 | 46298-46331 | CGACGGACTCAGCGACGCCGACCACGCCGCACTG |
|  |  | 38893-38923 | CGACGGAC--AGGTAC-CCGACCACGCCGAACTG |
| GTE6-D40 | 33 | 2648-2680 | CGACGACCTCGCTGACGAACTCGCCGAACGCGC |
|  |  | 484-516 | CGACGACCTCGATCGCGAGATCGCCGAACTCGC |
| GTE6-D41 | 32 | 46709-46740 | CGCGCTCGTGCTCGCCTCGATCGGCGCGGTCA |
|  |  | 24714-24745 | CGCGCCGGCGTTCGCCGCGATCGGCGCGATCA |
| GTE6-D42 | 30 | 32916-32945 | CGGCGGCAAGCTCGTCACGTCGCAGGTGCC |
|  |  | 12103-12132 | CGGCGGCAAGCTCGCCGCGTCGCTGCTGCC |
| GTE6-D43 | 30 | 33084-33113 | GACCGGCTGGCAGCAGGTCGCCTATCCGGC |
|  |  | 3121-3149 | GAGCGGCTAG-AGCAGATCGCCTATCCGGC |
| GTE6-D44 | 30 | 52677-52705 | CCGACCCG-ACGTGGACGTGAAGCGCGACA |
|  |  | 7985-8014 | CCGAACCGCACGTCGACGAGAAGCGCGACA |
| GTE6-D45 | 30 | 52511-52540 | CGAACGCGCAGGCGATCGCGTATTGCGTGC |
|  |  | 48598-48627 | CGCACGCGAAGGCGATCGCGAACGGCGTGC |
| GTE6-D46 | 30 | 39142-39171 | GCGGTACGAGCCGGCGCTCACTGCCCGAGA |
|  |  | 11689-11718 | GCGGGTCGAGATCGCGCTCACTGCCCGCGA |
| GTE6-D47 | 30 | 51856-51885 | CGGTCGAGCCGGTACTCACGCACGGCAAGA |
|  |  | 12258-12287 | CGGTCGAGCCGGTGCACACCGGCGGCATGA |
| GTE6-D48 | 29 | 47935-47960 | CAACCCGCTCGAC---CCGGTGCCGGCAT |
|  |  | 3647-3675 | CAACCCGCTCGACGAGCCGGTGCCGGCAT |
| GTE6-D49 | 29 | 47398-47426 | ACCCCGAGGTCGACACCGACGAGAAGGGC |
|  |  | 20658-20686 | ACCCCGACGTAGACACCGACGACATGGGC |
| GTE6-D50 | 29 | 33426-33453 | CGACGGC-ACGATCGTCGACGGCGACGTG |
|  |  | 33290-33318 | CGACGGCGAAGATCGTCGACGCCAACGTG |
| GTE6-D51 | 29 | 29418-29446 | GCCGGGCCGCCCGTACTGGCCGGGCGCAC |
|  |  | 2058-2086 | GCCGGGCAGCACGTGGTCGCCGGGCGCAC |
| GTE6-D52 | 28 | 12343-12370 | CGCCGAGCTACACCTCACGCTCGCGTTC |
|  |  | 10448-10475 | CGACGAGCTACACCGCCGGCTCGCGTTC |
| GTE6-D53 | 28 | 37577-37603 | CCACC-AGAGAGGCACGACCATGACCGA |
|  |  | 36038-36065 | CCACCTAGAAGGGCACGACCATGACCGA |
| GTE6-D54 | 28 | 53799-53825 | CAGCCGTG-CGACGCCGCCGGCACGATC |
|  |  | 4536-4563 | CAGCCGCGACGAGGCCGCCGGCACGTTC |
| GTE6-D55 | 28 | 11858-11885 | CTACTCGCTGCCGGCGTCGAGTCGGCCG |
|  |  | 8155-8181 | CTGCTCGCTGCCGGCGACGACT-GGCCG |
| GTE6-D56 | 28 | 30490-30515 | CGCGCT--CGGCGCCGAGGCGAACCTCG |
|  |  | 17146-17173 | CGCGCTGCCGGCGCCGAGGCGCATCTCG |
| GTE6-D57 | 28 | 24717-24744 | GCCGGCGTTCGCCGCGATCGGCGCGATC |
|  |  | 22774-22800 | GCCGGCAT-CGCCGCGATCGGTGCGCTC |
| GTE6-D58 | 28 | 14387-14414 | GAGGTCGACGCGCTGCTGAACGAGGCGA |
|  |  | 10008-10035 | GAGGCCGAGGGCCGGCTGAACGAGGCGA |
| GTE6-D59 | 28 | 49298-49325 | CGACACCCTCGAATCGAAGTACGACGAC |
|  |  | 26714-26741 | CGACACCCTCGACGCCGAGCACGACGAC |
| GTE6-D60 | 27 | 32385-32411 | GACGGCGACCGTGCCGGCGCTCGTCGA |
|  |  | 1593-1619 | GACGGCGACCGCGCCGGCACCCGACGA |
| GTE6-D61 | 27 | 9479-9505 | GCAGTCGATGCCGGCCGAGCCGGTCGA |
|  |  | 3174-3200 | GCCGTCGAAGCCGGCCGAGCAGTTCGA |
| GTE6-D62 | 27 | 9985-10011 | CCGAGGCGCTTGCACGGCTCGACGAGG |
|  |  | 4762-4788 | CCGAGGCGATCGCGTGGCTCGACGAGG |
| GTE6-D63 | 27 | 47691-47714 | GTTCGGGCAGCAG---ATCGCCGACCG |
|  |  | 11797-11823 | GTCCGGGCAGCAGCTGATCGCCGACCG |
| GTE6-D64 | 27 | 52444-52466 | CACCTCGTCGACGAC----GCCGGCCG |
|  |  | 29961-29987 | CACCTCGTCGACGACCAGGGCCGGCCG |
| GTE6-D65 | 27 | 30259-30283 | GGCTCGTCGACTCGGA-GCG-TGCCCG |
|  |  | 12531-12557 | GGCTCGTCGACTCGCATGCGCTGCCCG |
| GTE6-D66 | 27 | 55827-55853 | CCGACCCGCGAGATCGCCGAGACCGAG |
|  |  | 43612-43637 | CCGACGTGC-AGATCGCCGAGGCCGAG |
| GTE6-D67 | 26 | 18912-18937 | CGCCGCGTCGACGGGCTCGCGTGGCC |
|  |  | 1080-1105 | CGGCGGGTCGATGCGCTCGCGTGGCC |
| GTE6-D68 | 26 | 54440-54465 | CCGAAGCTCGCCGAGGTCGACGACGC |
|  |  | 7007-7032 | CCGAAGCTCGCCGCGCTGGCCGACGC |
| GTE6-D69 | 26 | 22671-22696 | CACCGCGATCGCCGGCGCCGCTACCG |
|  |  | 12703-12728 | CATCGCGATCGCCGGCGACGTGACCG |
| GTE6-D70 | 26 | 52235-52260 | CTGCTCGACGACGACCTCGCCGAGGA |
|  |  | 40310-40335 | CTGCTCGAATACGGACTCGCCGAGGA |
| GTE6-D71 | 26 | 25815-25837 | CACCGGGCCGGCCGGT---GCGCCGG |
|  |  | 16862-16887 | CACCGGGCCGGCCGATCAGGCGCCGG |
| GTE6-D72 | 25 | 45215-45239 | CCGCAGGCGATCGCCGGCACCGCAC |
|  |  | 16983-17007 | CCGCAGACGAACACCGGCACCGCAC |
| GTE6-D73 | 25 | 35489-35513 | TCGCGACCGCGCCGGCACCGATCAA |
|  |  | 19272-19296 | TCGTGACCGCGCCGGCGACGATCAA |
| GTE6-D74 | 25 | 32592-32616 | CGCGACGACCACGGCGAAGGGCGGC |
|  |  | 27588-27612 | CGCGACGACCAGGGCGACGGCCGGC |
| GTE6-D75 | 25 | 18916-18940 | GCGTCGACGGGCTCGCGTGGCCGAC |
|  |  | 3227-3249 | GCGTCGACGGGCT--CGTGCCCGAC |
| GTE6-D76 | 25 | 37761-37785 | GACCTGCTCGACAAGCTCAACACCG |
|  |  | 10440-10462 | GACCTGCTCGACGAGCT--ACACCG |
| GTE6-D77 | 25 | 48602-48626 | CGCGAAGGCGATCGCGAACGGCGTG |
|  |  | 529-553 | CGCGAAGGCGAAGGCCGACGGCGTG |
| GTE6-D78 | 25 | 40116-40140 | TCATGACGTGCGCACAGCTGGGCCT |
|  |  | 2299-2323 | TCAAGCCGGGCGCACAGCTGCGCCT |
| GTE6-D79 | 25 | 39783-39807 | CGCGCTACTCGCCGCCGACACCGAG |
|  |  | 11854-11878 | CGCGCTACTCGCTGCCGGCGTCGAG |
| GTE6-D80 | 25 | 17142-17166 | GCTGCGCGCTGCCGGCGCCGAGGCG |
|  |  | 11857-11881 | GCTACTCGCTGCCGGCGTCGAGTCG |
| GTE6-D81 | 25 | 51903-51927 | CGCCGGGCTCAACTCGACCAGCTGC |
|  |  | 44132-44155 | CGCCGGCCG-AACTCGACCAGCTGC |
| GTE6-D82 | 24 | 47177-47200 | CGCCGACGGGCACGCCGACCTCGC |
|  |  | 39346-39369 | CGCCGACGGGCACGCCGTCATCGC |
| GTE6-D83 | 24 | 45768-45791 | CCTCGACGGGCTGATCGACGCCGC |
|  |  | 39819-39842 | CCTCGACGGGCTGATCGAGGACGC |
| GTE6-D84 | 24 | 54113-54135 | GGCCGC-GACTACTCGGGCGGGCT |
|  |  | 51229-51252 | GGCCGCCGACTACTCGGGCGGGCT |
| GTE6-D85 | 24 | 17817-17840 | AGTACGAGGACGGCACCGAGAACG |
|  |  | 13662-13684 | AGTACGACGACGGCACCGA-AACG |
| GTE6-D86 | 24 | 30408-30431 | CACCCGGCGGCGGCACGCCGATCG |
|  |  | 16820-16842 | CACCCGGCT-CGGCACGCCGATCG |
| GTE6-D87 | 24 | 34518-34541 | CGTCGAGACCGCCGTGAACGACGC |
|  |  | 11872-11895 | CGTCGAGTCGGCCGTGAACGATGC |
| GTE6-D88 | 24 | 44802-44825 | ACCGGGATCGCCGCCGGCTACCGG |
|  |  | 34204-34227 | ACCGGTATGGGCGCCGGCTACCGG |
| GTE6-D89 | 24 | 50070-50093 | CGGCCGACGACGAGGCGCCGGTCG |
|  |  | 40752-40775 | CGGCCGACGACGAACCGCCGGCCG |
| GTE6-D90 | 24 | 11600-11620 | CTCGTCGCCGC---CGGCATCGTC |
|  |  | 5395-5418 | CTCGTCGCCGCGGCCGGCATCGTC |
| GTE6-D91 | 23 | 31762-31784 | CAAGCAGCCGTTCGAGTTCTACG |
|  |  | 19402-19424 | CAAGCCGCCGTTCGAGTTCTGCG |
| GTE6-D92 | 23 | 23772-23794 | GCTCGGGCAGCTGATCGCGCAGG |
|  |  | 1846-1867 | GCTCGG-CAGCTGATCACGCAGG |
| GTE6-D93 | 23 | 1320-1342 | CGGCCGGGTCGAAGAGGGCGGCG |
|  |  | 14607-14627 | CGGCCGG--CGAAGAGGGCGGCG |
| GTE6-D94 | 23 | 33456-33478 | CGGGTCGGCCGGCATCGCCGCGA |
|  |  | 22769-22789 | CGGGT--GCCGGCATCGCCGCGA |
| GTE6-D95 | 23 | 40090-40112 | CGAGGCGGCGACCGTGCTCGGCG |
|  |  | 32382-32403 | CGAGACGGCGACCGTGC-CGGCG |
| GTE6-D96 | 23 | 44247-44269 | GTGCCTGCTCGACCCCGAACCGT |
|  |  | 3608-3630 | GTGCCTGCTCGACGGCGAGCCGT |
| GTE6-D97 | 23 | 12094-12116 | GAACAAAGACGGCGGCAAGCTCG |
|  |  | 7162-7184 | GAACAAAGACGGCGAGAAGGTCG |
| GTE6-D98 | 23 | 22872-22894 | CGAGAACGCCGAGAAGTTCAACG |
|  |  | 7558-7580 | CGAGAACGCCGACGAGTTCAGCG |
| GTE6-D99 | 23 | 33762-33784 | CGGGCTCGGCGGCGACTGGTCGA |
|  |  | 31078-31100 | CGGGCTCGGCGCCGACGGGACGA |
| GTE6-D100 | 23 | 46389-46411 | GTCCGCCGGCGACGCGTCCAAGC |
|  |  | 34104-34126 | GTCCGCCGGCGACGTGTTCGAGC |
| GTE6-D101 | 23 | 49043-49065 | CGACCGAGGCAAGCGGGCCGTCG |
|  |  | 45948-45970 | CGACCAACGCAAGCGGGCCGACG |
| GTE6-D102 | 23 | 55182-55204 | CGGCGAGATCTACCCGTACAGCG |
|  |  | 47983-48005 | CGGCGCGATCCGCCCGTACAGCG |
| GTE6-D103 | 22 | 44355-44376 | CGTCGCCGCACACCGCGGCCAC |
|  |  | 25766-25787 | CGTCGTCGCACACCGCGGCCAC |
| GTE6-D104 | 22 | 46298-46319 | CGACGGACTCAGCGACGCCGAC |
|  |  | 5610-5631 | CGAGGGATTCAGCGACGCCGAC |
| GTE6-D105 | 22 | 53441-53462 | GATCGACGGCCGCCGGCTCGGC |
|  |  | 38728-38749 | GATCGTCGCCCGCCGGCTCGGC |
| GTE6-D106 | 22 | 49253-49274 | CGACGACGGGCTCGTGACCCGA |
|  |  | 3228-3248 | CGTCGACGGGCTCGTG-CCCGA |
| GTE6-D107 | 21 | 27252-27272 | CAGTGGGCCTATCAGGAGGGC |
|  |  | 7634-7654 | CAGTGGGCCTATCAGCAGGGC |
| GTE6-D108 | 21 | 7821-7841 | TCGACCTGCCGGCGACCGCGG |
|  |  | 558-578 | TCGACCTGCCGGTGACCGAGG |
| GTE6-D109 | 21 | 32516-32536 | CGGCCGCCGGCAGTGCCGACG |
|  |  | 3414-3434 | CGGCCGCCGGCAGTACCGCCG |
| GTE6-D110 | 21 | 15891-15911 | CGACCAGCGTGAAGGCGCTCG |
|  |  | 4654-4674 | CGAGCAACGTGAAGGCGCTCG |
| GTE6-D111 | 21 | 8841-8861 | GACCGTATCGGCCGTGTGCTG |
|  |  | 7721-7741 | GAACGTACCGGCCGTGTGCTG |
| GTE6-D112 | 21 | 50955-50975 | AGGCCGCGCTCGACGTGTACG |
|  |  | 10672-10692 | AGGCCGGGCTCGACGTGTTCG |
| GTE6-D113 | 21 | 47844-47864 | AGATCCCCGAGGATCTGTTCG |
|  |  | 15042-15062 | AGTTCCCCGAGGATCTGGTCG |
| GTE6-D114 | 21 | 50399-50418 | CCCCGAAGAGGTCGA-GCGCA |
|  |  | 15457-15477 | CCCCGAAGAGGTCGAGGCGCA |
| GTE6-D115 | 21 | 51147-51167 | TCGGCGCGATCGCGAACGCCG |
|  |  | 24014-24034 | TCGTCGCGCTCGCGAACGCCG |
| GTE6-D116 | 21 | 37574-37594 | ACCCCACCAGAGAGGCACGAC |
|  |  | 29686-29706 | ACCCCACCAGAGAGGACCGAC |
| GTE6-D117 | 21 | 41203-41223 | CGCCGACGGCACGTTCCTCGA |
|  |  | 33423-33443 | CGCCGACGGCACGATCGTCGA |
| GTE6-D118 | 21 | 42270-42290 | GCTCGCGCTCGCCGGGCACCT |
|  |  | 34769-34789 | GCTCGGGCTCGCCGGGCAGCT |
| GTE6-D119 | 20 | 30694-30713 | GTTCGGCAACCTGCTCGACG |
|  |  | 6763-6782 | GTTCGGCAACCTGCCCGACG |
| GTE6-D120 | 20 | 21748-21767 | CCGGCGCTGACGACATGGCT |
|  |  | 11430-11449 | CCGGCACTGACGACATGGCT |
| GTE6-D121 | 20 | 19366-19385 | GGCCGGTGTCCCTGGCCGTG |
|  |  | 14899-14918 | GGCCGGTGTCCGTGGCCGTG |
| GTE6-D122 | 20 | 33110-33129 | CGGCGGCACCGGTGTCGAGC |
|  |  | 15847-15866 | CGGCGGCACCGGTGTCGGGC |
| GTE6-D123 | 20 | 36473-36492 | GCATCGCCGGCACGCTCGCG |
|  |  | 18736-18755 | GCATCGCCGGCACGATCGCG |
| GTE6-D124 | 20 | 35949-35968 | CGCAGCACCCGAGTTCGGCA |
|  |  | 30317-30336 | CGCACCACCCGAGTTCGGCA |
| GTE6-D125 | 20 | 39219-39238 | TGAACCTCGTCGACGGCGAG |
|  |  | 1031-1050 | TGAACCTCGTCGCCGACGAG |
| GTE6-D126 | 20 | 56961-56980 | ACGCCGGCGACCAAACCGAC |
|  |  | 2407-2426 | ACGCCGGCGACCACGCCGAC |
| GTE6-D127 | 20 | 52242-52261 | ACGACGACCTCGCCGAGGAA |
|  |  | 2647-2666 | ACGACGACCTCGCTGACGAA |
| GTE6-D128 | 20 | 15568-15587 | GAACTTCACCGGCACGTTCG |
|  |  | 3842-3861 | GAATTTCACCGGCACGATCG |
| GTE6-D129 | 20 | 17199-17218 | GCCGGCGTTCGAGGTGCCCG |
|  |  | 4091-4110 | GCCGCCGTTCGAGGTGCACG |
| GTE6-D130 | 20 | 42489-42508 | GCGCCGGCAACGGGCTCGGG |
|  |  | 5170-5189 | GCGCCCGCGACGGGCTCGGG |
| GTE6-D131 | 20 | 34865-34884 | ACCTCGTGGCCGGCGACGTG |
|  |  | 6690-6709 | ACCACGAGGCCGGCGACGTG |
| GTE6-D132 | 20 | 17143-17162 | CTGCGCGCTGCCGGCGCCGA |
|  |  | 8155-8174 | CTGCTCGCTGCCGGCGACGA |
| GTE6-D133 | 20 | 25514-25533 | GGCTCGCGTCGAAGGATCTC |
|  |  | 8917-8936 | GGCTCGCGTCGCCGGATCTC |
| GTE6-D134 | 20 | 11266-11285 | CGACGGCGATCTGCCCGTCG |
|  |  | 9830-9849 | CGACGACGATCTGCCCGGCG |
| GTE6-D135 | 20 | 50720-50739 | CGTCGACGATCTGCTCGGCG |
|  |  | 9830-9849 | CGACGACGATCTGCCCGGCG |
| GTE6-D136 | 20 | 30953-30972 | GCCGACGCCGCGGCCGCGCA |
|  |  | 11327-11346 | GCCGTGGCCGCGGCCGCGCA |
| GTE6-D137 | 20 | 50046-50065 | CGGTCGCGTTCCTCGGCGAG |
|  |  | 12360-12379 | CGCTCGCGTTCCTCGGTGAG |
| GTE6-D138 | 20 | 36467-36486 | CCGCCGGCATCGCCGGCACG |
|  |  | 23267-23286 | CCGACGGCATCGCCGGCGCG |
| GTE6-D139 | 20 | 49026-49045 | TCGCTGACCTCGATCATCGA |
|  |  | 23476-23495 | TCACTGACCTCGATCAACGA |
| GTE6-D140 | 20 | 38697-38716 | TCGGGCGCGAGGTCGAGTCG |
|  |  | 26546-26565 | TCGGGCGCGAGGACGAGGCG |
| GTE6-D141 | 20 | 37811-37830 | CGACGTGAAGATCAAAAGCG |
|  |  | 28827-28846 | CGACGTGAAGAACAAGAGCG |
| GTE6-D142 | 20 | 32837-32856 | CGCACGCGACGACCAAGGCG |
|  |  | 32588-32607 | CGGACGCGACGACCACGGCG |
| GTE6-D143 | 20 | 48857-48876 | CACCATCGTCGACGGCGCCG |
|  |  | 33432-33451 | CACGATCGTCGACGGCGACG |
| GTE6-D144 | 20 | 46615-46634 | GCGCCGGCGCCGGCACCGAT |
|  |  | 35491-35510 | GCGACCGCGCCGGCACCGAT |
| GTE6-D145 | 20 | 52239-52258 | TCGACGACGACCTCGCCGAG |
|  |  | 36164-36183 | TCGTCGCCGACCTCGCCGAG |
| GTE6-D146 | 20 | 49883-49902 | GCCGCCACACTGCCCGGCGT |
|  |  | 37141-37160 | GCCGCCAAGCTGCCCGGCGT |
| GTE6-D147 | 20 | 46783-46802 | ACATGAACCTCGTCGCCGGC |
|  |  | 39216-39235 | ACGTGAACCTCGTCGACGGC |
| GTE6-D148 | 19 | 43754-43772 | CGCCGGCATCCTCGGGATC |
|  |  | 2875-2893 | CGCCGGCATCCTCGCGATC |
| GTE6-D149 | 19 | 53516-53534 | GCAGCTGGCCGCCGAGGAT |
|  |  | 3435-3453 | GCAGCTGGCCGCCGAGCAT |
| GTE6-D150 | 19 | 39673-39691 | TGGATGACCGACCCGAACG |
|  |  | 3493-3511 | TGGATGAACGACCCGAACG |
| GTE6-D151 | 19 | 37941-37959 | CTCGACGACGGCAGCCTGC |
|  |  | 4206-4224 | CTCGACGACGGCAGCGTGC |
| GTE6-D152 | 19 | 50812-50830 | GCGCGCGCTGATCGCCGAG |
|  |  | 4670-4688 | GCTCGCGCTGATCGCCGAG |
| GTE6-D153 | 19 | 12570-12588 | AGATCCTGCGCGCGCTCGG |
|  |  | 7800-7818 | AGATCCTGCGCGCGATCGG |
| GTE6-D154 | 19 | 9934-9952 | TGCAGGATGAGTTCGCCGA |
|  |  | 9316-9334 | TGCAGGATGAGTTCGACGA |
| GTE6-D155 | 19 | 11885-11903 | GTGAACGATGCGACCGGGC |
|  |  | 10572-10590 | GTGAACGATGCGACCGCGC |
| GTE6-D156 | 19 | 33843-33861 | CGTCGCGACCACCGACGGC |
|  |  | 11254-11272 | CGTCCCGACCACCGACGGC |
| GTE6-D157 | 19 | 46643-46661 | CGTCGCCGCCGGCATGGTC |
|  |  | 11602-11620 | CGTCGCCGCCGGCATCGTC |
| GTE6-D158 | 19 | 24171-24189 | CTCGGCGGTGCGCACCGCG |
|  |  | 22593-22611 | CTCGGCGGTGCGCGCCGCG |
| GTE6-D159 | 19 | 16619-16637 | GACGACCACCACGCCGGCG |
|  |  | 26655-26673 | GACGACGACCACGCCGGCG |
| GTE6-D160 | 19 | 30875-30893 | ATCGCCGACGTGATCTCGG |
|  |  | 30608-30626 | ATCGGCGACGTGATCTCGG |
| GTE6-D161 | 19 | 41197-41215 | GCTCGGCGCCGACGGCACG |
|  |  | 31081-31099 | GCTCGGCGCCGACGGGACG |
| GTE6-D162 | 19 | 35491-35509 | GCGACCGCGCCGGCACCGA |
|  |  | 31713-31731 | GCGACCCCGCCGGCACCGA |
| GTE6-D163 | 19 | 42269-42287 | TGCTCGCGCTCGCCGGGCA |
|  |  | 32996-33014 | TGCTCGCGCTCACCGGGCA |
| GTE6-D164 | 19 | 51789-51807 | CGCGACGACGAGCACCCGG |
|  |  | 34239-34257 | CGCGACGACGAGCGCCCGG |
| GTE6-D165 | 19 | 40696-40714 | GGCACCCGGCGTCGACGAC |
|  |  | 39106-39124 | GGCACTCGGCGTCGACGAC |
| GTE6-D166 | 19 | 47782-47800 | CGTCGACCCGAAGTGCGAC |
|  |  | 46679-46697 | CGTCGACCCGAAGGGCGAC |
| GTE6-D167 | 19 | 53399-53417 | CGACGACGCCGAGGTCGAC |
|  |  | 47393-47411 | CGACGACCCCGAGGTCGAC |
| GTE6-D168 | 19 | 50342-50360 | ATCGCCCGTCGCCGACGTG |
|  |  | 48015-48033 | ATCGCCCGTCGCCGCCGTG |
| GTE6-D169 | 18 | 21572-21589 | CGGGCTCGACGTGTTCGA |
|  |  | 10676-10693 | CGGGCTCGACGTGTTCGA |
| GTE6-D170 | 18 | 34363-34380 | TCGCTGGCTAGGGGCCGG |
|  |  | 14197-14214 | TCGCTGGCTAGGGGCCGG |
| GTE6-D171 | 18 | 34365-34382 | GCTGGCTAGGGGCCGGGC |
|  |  | 17626-17643 | GCTGGCTAGGGGCCGGGC |
| GTE6-D172 | 18 | 8781-8798 | GACATACGGCCGGAACTG |
|  |  | 637-654 | GACCTACGGCCGGAACTG |
| GTE6-D173 | 18 | 39793-39810 | GCCGCCGACACCGAGGGC |
|  |  | 1459-1476 | GCCGACGACACCGAGGGC |
| GTE6-D174 | 18 | 43162-43179 | GACCAACCGACATGACCG |
|  |  | 4463-4480 | GACCAACCGACATGATCG |
| GTE6-D175 | 18 | 44661-44678 | CTCGCCGGCTACAGCCTC |
|  |  | 4710-4727 | CTCGTCGGCTACAGCCTC |
| GTE6-D176 | 18 | 51438-51455 | GGCCGAGCACGGCGCCGC |
|  |  | 5772-5789 | GGCCGAGCACGGAGCCGC |
| GTE6-D177 | 18 | 12840-12857 | CGATCGAGGCCGCCGGCG |
|  |  | 8578-8595 | CGATCGAGGCCGCCGTCG |
| GTE6-D178 | 18 | 19019-19036 | GGCGGGGCAGCTGGCCGC |
|  |  | 9065-9082 | GGCCGGGCAGCTGGCCGC |
| GTE6-D179 | 18 | 10046-10063 | CGAGGTCGACGACGACGG |
|  |  | 9713-9730 | CGAAGTCGACGACGACGG |
| GTE6-D180 | 18 | 41294-41311 | GCGATCGACGACGACGAG |
|  |  | 9756-9773 | GCGAGCGACGACGACGAG |
| GTE6-D181 | 18 | 29514-29531 | GTACGGCATCGACACCGA |
|  |  | 17484-17501 | GTTCGGCATCGACACCGA |
| GTE6-D182 | 18 | 22818-22835 | GATCAAGACCGCGTTCGC |
|  |  | 18025-18042 | GATCGAGACCGCGTTCGC |
| GTE6-D183 | 18 | 41293-41310 | CGCGATCGACGACGACGA |
|  |  | 18851-18868 | CGCGATCGACGGCGACGA |
| GTE6-D184 | 18 | 55276-55293 | GCCGGCTACCGCGTGATG |
|  |  | 19084-19101 | GCCGGCTACCGCGTGGTG |
| GTE6-D185 | 18 | 39716-39733 | TCGAGCAGCTGATCGCGC |
|  |  | 23774-23791 | TCGGGCAGCTGATCGCGC |
| GTE6-D186 | 18 | 56065-56082 | CGCGGCCGGGCTGCAGAA |
|  |  | 24399-24416 | CGCTGCCGGGCTGCAGAA |
| GTE6-D187 | 18 | 55850-55867 | CGAGCACCGCGTCGGGCA |
|  |  | 25142-25159 | CGAGCGCCGCGTCGGGCA |
| GTE6-D188 | 18 | 46526-46543 | GGGCACCCTGCTCGACGG |
|  |  | 28863-28880 | GGGCACGCTGCTCGACGG |
| GTE6-D189 | 18 | 48152-48169 | GCCGACACCGCGGCCGCG |
|  |  | 30953-30970 | GCCGACGCCGCGGCCGCG |
| GTE6-D190 | 18 | 44495-44512 | CCGAGCGCGCGGCGTGCT |
|  |  | 37220-37237 | CCGAGCGCGCGGCGTCCT |
| GTE6-D191 | 18 | 53359-53376 | AACGACTCGCGAAGGTAC |
|  |  | 38162-38179 | AACGACTCGCGACGGTAC |
| GTE6-D192 | 18 | 51782-51799 | CATCGACCGCGACGACGA |
|  |  | 39010-39027 | CATCGACCGCGACGAGGA |
| GTE6-D193 | 18 | 44054-44071 | CGGCCGTCGACGCGCTGC |
|  |  | 40650-40667 | CGGCCGTCGACGCGCAGC |
| GTE6-D194 | 18 | 56104-56121 | GCAGCTGCCCGACAACCT |
|  |  | 45436-45453 | GCAGCTGCCCGACGACCT |
| GTE6-D195 | 17 | 35491-35507 | GCGACCGCGCCGGCACC |
|  |  | 1597-1613 | GCGACCGCGCCGGCACC |
| GTE6-D196 | 17 | 31515-31531 | CGCTGTGGCAGCCGGGC |
|  |  | 2048-2064 | CGCTGTGGCAGCCGGGC |
| GTE6-D197 | 17 | 38438-38454 | CCCACCAGCGAAGGACA |
|  |  | 5924-5940 | CCCACCAGCGAAGGACA |
| GTE6-D198 | 17 | 49448-49464 | GCTGCTCGCCGCGCTCG |
|  |  | 23922-23938 | GCTGCTCGCCGCGCTCG |
| GTE6-D199 | 17 | 50831-50847 | ATCGAGGTCGACGACGA |
|  |  | 35101-35117 | ATCGAGGTCGACGACGA |
| GTE6-D200 | 16 | 11466-11481 | GCTCGTCGCTGTCGGG |
|  |  | 1686-1701 | GCTCGTCGCTGTCGGG |
| GTE6-D201 | 16 | 22609-22624 | GCGAAGTCGATCGCCG |
|  |  | 2792-2807 | GCGAAGTCGATCGCCG |
| GTE6-D202 | 16 | 16881-16896 | GCGCCGGCGGCCGGGC |
|  |  | 2940-2955 | GCGCCGGCGGCCGGGC |
| GTE6-D203 | 16 | 54449-54464 | GCCGAGGTCGACGACG |
|  |  | 10044-10059 | GCCGAGGTCGACGACG |
| GTE6-D204 | 16 | 50833-50848 | CGAGGTCGACGACGAC |
|  |  | 10046-10061 | CGAGGTCGACGACGAC |
| GTE6-D205 | 16 | 11815-11830 | CGCCGACCGCCGGCGG |
|  |  | 10265-10280 | CGCCGACCGCCGGCGG |
| GTE6-D206 | 16 | 41018-41033 | GAGCAGATCAAGGGGC |
|  |  | 12029-12044 | GAGCAGATCAAGGGGC |
| GTE6-D207 | 16 | 17626-17641 | GCTGGCTAGGGGCCGG |
|  |  | 14199-14214 | GCTGGCTAGGGGCCGG |
| GTE6-D208 | 16 | 42111-42126 | ACGATCCGGCCGACGG |
|  |  | 19996-20011 | ACGATCCGGCCGACGG |
| GTE6-D209 | 16 | 36123-36138 | CGTGGCCGTCGCCGGC |
|  |  | 22695-22710 | CGTGGCCGTCGCCGGC |
| GTE6-D210 | 16 | 30568-30583 | CGGCGGGTTCGCGTCG |
|  |  | 25686-25701 | CGGCGGGTTCGCGTCG |
| GTE6-D211 | 16 | 44867-44882 | CCCGAACGACGGCATC |
|  |  | 34602-34617 | CCCGAACGACGGCATC |
| GTE6-D212 | 16 | 56494-56509 | CGACGGCAAGCCGATC |
|  |  | 48437-48452 | CGACGGCAAGCCGATC |
| GTE6-D213 | 15 | 39115-39129 | CGTCGACGACCTCGA |
|  |  | 481-495 | CGTCGACGACCTCGA |
| GTE6-D214 | 15 | 19021-19035 | CGGGGCAGCTGGCCG |
|  |  | 740-754 | CGGGGCAGCTGGCCG |
| GTE6-D215 | 15 | 46786-46800 | TGAACCTCGTCGCCG |
|  |  | 1031-1045 | TGAACCTCGTCGCCG |
| GTE6-D216 | 15 | 56466-56480 | TCAAGCCGGGCGCAC |
|  |  | 2299-2313 | TCAAGCCGGGCGCAC |
| GTE6-D217 | 15 | 25301-25315 | CGCACCTCGCCGGCG |
|  |  | 2757-2771 | CGCACCTCGCCGGCG |
| GTE6-D218 | 15 | 5084-5098 | CAAGGTGCGCCGCGA |
|  |  | 3039-3053 | CAAGGTGCGCCGCGA |
| GTE6-D219 | 15 | 19024-19038 | GGCAGCTGGCCGCCG |
|  |  | 3434-3448 | GGCAGCTGGCCGCCG |
| GTE6-D220 | 15 | 33429-33443 | CGGCACGATCGTCGA |
|  |  | 3851-3865 | CGGCACGATCGTCGA |
| GTE6-D221 | 15 | 43745-43759 | CACCGAGGCCGCCGG |
|  |  | 3914-3928 | CACCGAGGCCGCCGG |
| GTE6-D222 | 15 | 52612-52626 | GCGTGGCTCGACGAG |
|  |  | 4773-4787 | GCGTGGCTCGACGAG |
| GTE6-D223 | 15 | 40171-40185 | GCTGCCGTTCTGGGA |
|  |  | 8334-8348 | GCTGCCGTTCTGGGA |
| GTE6-D224 | 15 | 22126-22140 | GGCGCGTTCGAGGAC |
|  |  | 8769-8783 | GGCGCGTTCGAGGAC |
| GTE6-D225 | 15 | 9563-9577 | CGAGGAACGGCTCGC |
|  |  | 8909-8923 | CGAGGAACGGCTCGC |
| GTE6-D226 | 15 | 10048-10062 | AGGTCGACGACGACG |
|  |  | 9823-9837 | AGGTCGACGACGACG |
| GTE6-D227 | 15 | 35103-35117 | CGAGGTCGACGACGA |
|  |  | 10046-10060 | CGAGGTCGACGACGA |
| GTE6-D228 | 15 | 19563-19577 | CGAACCGGCCGCAGG |
|  |  | 10243-10257 | CGAACCGGCCGCAGG |
| GTE6-D229 | 15 | 45436-45450 | GCAGCTGCCCGACGA |
|  |  | 10958-10972 | GCAGCTGCCCGACGA |
| GTE6-D230 | 15 | 55432-55446 | GCCGAGGTCGAGGCG |
|  |  | 11642-11656 | GCCGAGGTCGAGGCG |
| GTE6-D231 | 15 | 25864-25878 | GCCGACGACGCGCAG |
|  |  | 11672-11686 | GCCGACGACGCGCAG |
| GTE6-D232 | 15 | 18082-18096 | CGCCGGCGGCAAGCG |
|  |  | 12850-12864 | CGCCGGCGGCAAGCG |
| GTE6-D233 | 15 | 32917-32931 | GGCGGCAAGCTCGTC |
|  |  | 13625-13639 | GGCGGCAAGCTCGTC |
| GTE6-D234 | 15 | 44059-44073 | GTCGACGCGCTGCTG |
|  |  | 14390-14404 | GTCGACGCGCTGCTG |
| GTE6-D235 | 15 | 41133-41147 | CCGAGGCCGGCGAGG |
|  |  | 14691-14705 | CCGAGGCCGGCGAGG |
| GTE6-D236 | 15 | 39277-39291 | CGACGGGCAGGTGCT |
|  |  | 15079-15093 | CGACGGGCAGGTGCT |
| GTE6-D237 | 15 | 40366-40380 | CGGCGCCCGAGGTGC |
|  |  | 16939-16953 | CGGCGCCCGAGGTGC |
| GTE6-D238 | 15 | 37909-37923 | ACGATCGCGGCCGCG |
|  |  | 18747-18761 | ACGATCGCGGCCGCG |
| GTE6-D239 | 15 | 53399-53413 | CGACGACGCCGAGGT |
|  |  | 19624-19638 | CGACGACGCCGAGGT |
| GTE6-D240 | 15 | 50855-50869 | CTCACCCTGTACACG |
|  |  | 20924-20938 | CTCACCCTGTACACG |
| GTE6-D241 | 15 | 26988-27002 | CGGCATGGACCCGAA |
|  |  | 22314-22328 | CGGCATGGACCCGAA |
| GTE6-D242 | 15 | 33854-33868 | CCGACGGCACGTACT |
|  |  | 26264-26278 | CCGACGGCACGTACT |
| GTE6-D243 | 15 | 35982-35996 | GCAGGACTTCGCCGG |
|  |  | 26799-26813 | GCAGGACTTCGCCGG |
| GTE6-D244 | 15 | 44833-44847 | CGCTCGGGCTCGGCA |
|  |  | 29195-29209 | CGCTCGGGCTCGGCA |
| GTE6-D245 | 15 | 30818-30832 | AACCTGCTCGACGCG |
|  |  | 30701-30715 | AACCTGCTCGACGCG |
| GTE6-D246 | 15 | 36676-36690 | CGTGACCGGCGACGG |
|  |  | 32220-32234 | CGTGACCGGCGACGG |
| GTE6-D247 | 15 | 44943-44957 | CTCGACGACTGGGCG |
|  |  | 34288-34302 | CTCGACGACTGGGCG |
| GTE6-D248 | 15 | 46428-46442 | CTGAAAGCCGGCACC |
|  |  | 35599-35613 | CTGAAAGCCGGCACC |
| GTE6-D249 | 15 | 53806-53820 | GCGACGCCGCCGGCA |
|  |  | 39629-39643 | GCGACGCCGCCGGCA |
| GTE6-D250 | 15 | 55628-55642 | CGACGACTGGGCCGA |
|  |  | 45924-45938 | CGACGACTGGGCCGA |
| GTE6-D251 | 15 | 51051-51065 | TCGCCGCGCTCGTCG |
|  |  | 49603-49617 | TCGCCGCGCTCGTCG |
| GTE6-D252 | 15 | 55921-55935 | CAGCGAGGACTCGAC |
|  |  | 49966-49980 | CAGCGAGGACTCGAC |
| GTE8-I1 | 45 | 61772-61816 | CGGTGATTGTTCGCGATGACGACGAGATCATCGACGACGACGACG |
|  |  | 43836-43795 | CGGTGATCGT---CGACGACGACGAGATCGTGGTGGACGACGACG |
| GTE8-I2 | 43 | 47400-47442 | CTTCTTGGCCTTCGGGACCTTGCGGTATCCCTCGGCGGGGCCG |
|  |  | 24416-24374 | CTTCTTGCCCTTCCGGACCTTGCGGCACTTGTCGACGCGGCCG |
| GTE8-I3 | 37 | 54497-54533 | CTCCCGGACGCCGAACGTCACGCCGTCGAGAGCGCAG |
|  |  | 12896-12860 | CTCCAGGACGCCGAACGGATCGACGTCGGGATCGGAG |
| GTE8-I4 | 37 | 39192-39228 | TGTCTCCGAGCAGGCCGACGTCGCCGTCGTCGATCGC |
|  |  | 28000-27965 | TGTCTCCGAGCTGGCCGACCTCTTCGTGGACG-TCGC |
| GTE8-I5 | 36 | 57386-57421 | CGGCCGTCGTCGCACCGACACCGGCGCCGCAACCGC |
|  |  | 262-227 | CGGACGCCGCCGGGCTGCCACCGCCGCCGCAACCGC |
| GTE8-I6 | 34 | 52495-52528 | TCGGTGTAGGTGACCTGGTCGCTGTCGGCGTCCG |
|  |  | 11360-11327 | TCGGCGTCGGTGTCGTCGTCCTTGTCGGCGTCCG |
| GTE8-I7 | 33 | 47695-47726 | GGCCGTCAATGAGCATCT-TGGTCAGACGCTCG |
|  |  | 38729-38697 | GGCCGTCAATGAGCACCTCCAGGCAGACGGTCG |
| GTE8-I8 | 31 | 52198-52228 | ATCGCCTCCATCTCCTCGCTCGGGAGATGGT |
|  |  | 28592-28562 | ATCGCCTCCATCTCGGCGCTCGTGATCTGGT |
| GTE8-I9 | 31 | 16481-16511 | GACCGGCGCCGTCGTCACTGCGACGACCGCG |
|  |  | 8143-8113 | GACCGGCGCCGTCGCCCCTTCGTTGATCGCG |
| GTE8-I10 | 31 | 22744-22774 | AGTTCGCGCTCGGCTTCGGCAAGAGCGCGCC |
|  |  | 11486-11457 | AGTTCGCGCTCGGCTTTGTC-GGCGCGCGCC |
| GTE8-I11 | 29 | 58642-58670 | CGCGGCCGACGCTCTCGCGCTGCTCGACG |
|  |  | 47891-47863 | CGCGGCCGACGACCTCGTGTTCCTCGACG |
| GTE8-I12 | 28 | 38027-38054 | CCCACTGCTTCAGCCGCTCGTCGGTGAT |
|  |  | 6815-6788 | CCCACTGGTCCCGCTGCTCGTCGGTGAT |
| GTE8-I13 | 27 | 40585-40611 | CAGGTCGGGGTACTTCGCGACGATCTC |
|  |  | 26994-26968 | CAGGTCGAGGATCTTCGCGACGATCTC |
| GTE8-I14 | 26 | 52147-52172 | TCGGGGACCGTCTTGCCGACGGTGGG |
|  |  | 11336-11311 | TCGGCGTCCGTCTTGCCGCCGTTGGG |
| GTE8-I15 | 24 | 39201-39224 | GCAGGCCGACGTCGCCGTCGTCGA |
|  |  | 10976-10953 | GCTGGCCGACGTCGCGGCCGTCGA |
| GTE8-I16 | 24 | 35248-35271 | CGACGTCGCCGAGCGCGTCGAGGA |
|  |  | 27697-27674 | CGAGGTCCCCGAGCGCGTCGACGA |
| GTE8-I17 | 23 | 20467-20489 | GCTCGCTGATCGGCGCCATCTTC |
|  |  | 18708-18686 | GCTCGTTGATCGGCGCCATCTTC |
| GTE8-I18 | 23 | 30580-30602 | GAACGCCGACCGCATCGCCATCG |
|  |  | 10771-10749 | GAACGCCGACCGGTTCGCCATCG |
| GTE8-I19 | 23 | 14014-14035 | TGTTCCTG-GGTCGGGCCTCTCG |
|  |  | 13917-13895 | TGGTCCTGTGGTCGGGCCTCTCG |
| GTE8-I20 | 22 | 46050-46071 | GCGACGATGTCGAAGGTGCCGT |
|  |  | 3703-3682 | GCGCCGACGTCGAAGGTGCCGT |
| GTE8-I21 | 22 | 63600-63621 | AAGATCGGTGAGCTGCGCAAGG |
|  |  | 37571-37550 | AAGATCGCCGAGCTGCGCAAGG |
| GTE8-I22 | 22 | 63123-63143 | GCGG-TCGCCGACGAGGTCGCG |
|  |  | 45551-45530 | GCGGATCGCCGACGAGGTCGCG |
| GTE8-I23 | 21 | 44915-44935 | TCCTCGACGCGCTCGGCGACG |
|  |  | 35271-35251 | TCCTCGACGCGCTCGGCGACG |
| GTE8-I24 | 21 | 47368-47388 | CTTCCAGATGGTGTGTGTCAC |
|  |  | 21377-21357 | CTTCCAGATGGTGTGCGTCAC |
| GTE8-I25 | 21 | 42828-42847 | TCGCGCTCGATCTC-CTGTCG |
|  |  | 7701-7681 | TCGCGCTCGATCTCGCTGTCG |
| GTE8-I26 | 21 | 22521-22541 | GTCACCCAGAACACCCCGTCG |
|  |  | 14245-14225 | GTCACCGAGAACACCCCGGCG |
| GTE8-I27 | 21 | 56230-56250 | ACTCGCCACCGGACGACACCA |
|  |  | 36306-36286 | ACCCGCCGCCGGACGACACCA |
| GTE8-I28 | 21 | 55279-55299 | CTCGACGCCGGGTGGCGCAGC |
|  |  | 36355-36335 | CTCGACGCCGGTTGGCACAGC |
| GTE8-I29 | 20 | 46950-46969 | ACCGCGGCCGGACGGCCATC |
|  |  | 40772-40753 | ACCGCGGCCGGACGGCCATC |
| GTE8-I30 | 19 | 29126-29144 | GCAGCGGTCCCGGCCGACG |
|  |  | 18526-18508 | GCCGCGGTCCCGGCCGACG |
| GTE8-I31 | 19 | 45893-45911 | CCGCTCGTTCTTGTGGATC |
|  |  | 32529-32511 | CCGCACGTTCTTGTGGATC |
| GTE8-I32 | 17 | 40587-40603 | GGTCGGGGTACTTCGCG |
|  |  | 7856-7840 | GGTCGGGGTACTTCGCG |
| GTE8-I33 | 17 | 45679-45695 | CGTCGACCTTCAGCTTC |
|  |  | 18024-18008 | CGTCGACCTTCAGCTTC |
| GTE8-I34 | 16 | 49028-49043 | GTCGAGGATCTTCTTG |
|  |  | 21623-21608 | GTCGAGGATCTTCTTG |
| GTE8-I35 | 16 | 51047-51062 | AGGTCGCCGAGCGACT |
|  |  | 37352-37337 | AGGTCGCCGAGCGACT |
| GTE8-I36 | 16 | 64179-64194 | AAGACCTGACCCCGGT |
|  |  | 46098-46083 | AAGACCTGACCCCGGT |
| GTE8-D1 | 141 | 29568-29707 | GCGAGCTTCGAGGGCACCGGCTCGTTCTCGGCCGTCGCAGCGGCGCACCTGCTGGCGTCGATGGAGGGGCTCGGGTCCTTCGCCGCCAGTC-AGCTCGCGCACCTCTTCGCGAGCTTCACTGGGACTGGGGCCTTCTCGGC |
|  |  | 29352-29491 | GCGAGCTTCGAGGGGACCGGCGACTTCCCGGCCTCCCTCGCTGCTCACCTGAACGCGACAGTCGAGGCGACCGGCTCCTTC-CCGGCAGCCGCGGTGGCCCACCTGCTCGCGACCCTCACTGCCACCGGCGCATTCTCGGC |
| GTE8-D2 | 98 | 19686-19777 | GCCGGGGTCAAGGAGGCCGTCCAGGCGCTCGCGCCGATCATCCAGATCG-TCGGCAG--CGTCCTGC---TCACCGTGCTCGGACCGGCGCTGACCGA |
|  |  | 19020-19117 | GCCGGGGTCAACCAGCTCGACCAGGCGATGGCGAAGCTCTCGCCGAACGCGCAGCAGTTCGTCCGGCAGATCCACGCGCTCGGCCCGGCCTGGACCGA |
| GTE8-D3 | 44 | 49508-49551 | GCCGCCGAGTTCCTTCAGCCGCTCCTTCATGACCTCGCGCTCGG |
|  |  | 38226-38269 | GCCGCCGAGCCGGATCAGCCGCTCCATCATGAACACGCCCGCGG |
| GTE8-D4 | 43 | 29665-29707 | CGCACCTCTTCGCGAGCTTCACTGGGACTGGGGCCTTCTCGGC |
|  |  | 29341-29383 | CGCAGCTCCTCGCGAGCTTCGAGGGGACCGGCGACTTCCCGGC |
| GTE8-D5 | 42 | 8499-8538 | CGGACGTCGC--AGGACCACGACACCGACGGTGACAAGGTCG |
|  |  | 11327-11368 | CGGACGCCGACAAGGACGACGACACCGACGCCGACAAGGACG |
| GTE8-D6 | 42 | 4707-4748 | CTCGTCGACAACCTCTCGATGAAGCTCCAGGCGCTCCTGGGC |
|  |  | 1534-1575 | CTCGCCGACGACGACCCGCTGATGTTCCAGGCGCTCCTGCGC |
| GTE8-D7 | 42 | 28331-28371 | ACGCCACC-TCGGCCAGCTTCGCCGCGCTCATCGGCGCCTCG |
|  |  | 18340-18380 | ACGCCGCCGTCGGCGGGCT-CACCGCGCTCATCGGTGCCGCG |
| GTE8-D8 | 40 | 58638-58675 | CCACCGCGGCCGACGCTCTCGCGCTGCTCGAC--GCTGCT |
|  |  | 32728-32767 | CCACCGCGGTCGGCGCTCTCGCGCTGATCGTCTGGCTGCT |
| GTE8-D9 | 36 | 64542-64574 | CGGCGATGGA-GA--TGATCGCGAAGATCGAAGCCG |
|  |  | 62422-62457 | CGGCGATGGATGACCTGCTCGCGCAGATCGAAGCCG |
| GTE8-D10 | 35 | 28974-29008 | GCGCAACAGCGCTGACGGCACCTTCGTCGCTGGCG |
|  |  | 3671-3702 | GCGCAACGGCG---ACGGCACCTTCGACGTCGGCG |
| GTE8-D11 | 33 | 61197-61229 | AAACGTACTCTGACCTGCTGCTTAACGAGGCGT |
|  |  | 61132-61163 | AAACATACTCTGACCTGC-GATTATCCAGGGGT |
| GTE8-D12 | 30 | 48894-48923 | TCGTTGAACAGGCCCATCTTGCCGGTGCGC |
|  |  | 42489-42518 | TCGTTGAACAGGCTCATCTTCGCGGTGCGC |
| GTE8-D13 | 29 | 52306-52334 | TCCGTATGGGCGGCACTATGGGCGGCACT |
|  |  | 52234-52262 | TCGGTATGGGCGGCACTATGGGCGGCACT |
| GTE8-D14 | 29 | 17989-18017 | CGAAGCTCGACGCCACCCGGAAGCTGAAG |
|  |  | 5439-5467 | CGAAGAACGCCGCCGCTCGGAAGCTGAAG |
| GTE8-D15 | 29 | 40704-40732 | CGAAGACCTCGGAGAAGTCGGCCAGCTCG |
|  |  | 27966-27994 | CGACGTCCACGAAGAGGTCGGCCAGCTCG |
| GTE8-D16 | 27 | 39203-39229 | AGGCCGACGTCGCCGTCGTCGATCGCG |
|  |  | 18016-18042 | AGGTCGACGTCGCCGTCGAGGCTCGCG |
| GTE8-D17 | 27 | 27896-27922 | TCATCCAGAACGTCATCGACACGATCC |
|  |  | 27098-27124 | TCATCCAGAACACCATCGGCACCATCC |
| GTE8-D18 | 27 | 30016-30039 | CGTCGACGATCACCG---CGTCGGTCG |
|  |  | 43822-43848 | CGTCGACGATCACCGGCTCCTCGGTCG |
| GTE8-D19 | 26 | 41300-41325 | GCGCCATCGAGCGCGGCGTCGCCCTC |
|  |  | 34292-34315 | GCGCCA--GAGCGCGGCGTCGCCGTC |
| GTE8-D20 | 26 | 63707-63732 | GGTCATGAAGGACGCCTACCTGCGCG |
|  |  | 32373-32398 | GGGCAAGAAGCTCGCCTACCTGCGCG |
| GTE8-D21 | 25 | 51831-51855 | GCCGCCGTCGCCGTCGACGATCGCG |
|  |  | 39205-39229 | GCCGACGTCGCCGTCGTCGATCGCG |
| GTE8-D22 | 24 | 31505-31528 | AAGGTCGCCGACGACTTCCTCGGC |
|  |  | 20805-20828 | AAGGCCGTCGACAACTTCCTCGGC |
| GTE8-D23 | 24 | 57260-57283 | CGTCGAGCGGGAGCGTCGAGGGTC |
|  |  | 54834-54857 | CGTCGAGCGGGACCGTTGCGGGTC |
| GTE8-D24 | 23 | 33046-33068 | CCTGGACTACCTCACCGGCGATC |
|  |  | 17247-17269 | CCTCGACTACTTCACCGGCGATC |
| GTE8-D25 | 23 | 58201-58223 | CCGTCGGCCGCTGCGCATCGCGA |
|  |  | 18055-18076 | CCGTCG-CCGCCGCGCATCGCGA |
| GTE8-D26 | 22 | 13368-13389 | CACGCGATCGCGATGCCGGTCC |
|  |  | 4278-4299 | CACGCGATCGCGATGTCGATCC |
| GTE8-D27 | 22 | 23668-23689 | CCGGATCAAGGTGCGCAACTTC |
|  |  | 13115-13136 | CCGGATCAAGGTGCGCGACATC |
| GTE8-D28 | 21 | 27568-27587 | CGGC-TCCAGGTCGAGGTCGG |
|  |  | 104-124 | CGGCATCCAGGTCGAGGTCGG |
| GTE8-D29 | 21 | 54652-54672 | GACCGCGCCGGCCGGATCGCC |
|  |  | 153-173 | GACCGGGCCCGCCGGATCGCC |
| GTE8-D30 | 21 | 13209-13229 | GCGGTGGCGGGGTCGAGCGAG |
|  |  | 5001-5021 | GCCGTGGCGGGGTCGGGCGAG |
| GTE8-D31 | 21 | 61622-61642 | GGATCACGATCCCGGACGCCG |
|  |  | 7548-7568 | GGCTCACGATCCCGGACGGCG |
| GTE8-D32 | 21 | 22446-22466 | TTCGGCCGCGAGGACGAGGCG |
|  |  | 17970-17990 | TTCGGCCGCGAGGCCCAGGCG |
| GTE8-D33 | 21 | 44914-44934 | CTCCTCGACGCGCTCGGCGAC |
|  |  | 27673-27693 | CTCGTCGACGCGCTCGGGGAC |
| GTE8-D34 | 21 | 32416-32436 | TGGAACGAGCTGGTCTACGAC |
|  |  | 28126-28146 | TGGAACGAGCTGTTCTACAAC |
| GTE8-D35 | 21 | 62916-62936 | ATCGCGAAGATCTCGGCGAAG |
|  |  | 40277-40297 | ATCCCGAAGATCTCGTCGAAG |
| GTE8-D36 | 20 | 41515-41534 | GGTGGGGATGCCGTCGGCCG |
|  |  | 14942-14961 | GGCGGGGATGCCGTCGGCCG |
| GTE8-D37 | 20 | 57281-57300 | GTCCCCGGCGCCGTCGGTGG |
|  |  | 28513-28532 | GTCCCCGGCGCCATCGGTGG |
| GTE8-D38 | 19 | 54292-54310 | CGACGACACCGGCGCCGAC |
|  |  | 11343-11361 | CGACGACACCGACGCCGAC |
| GTE8-D39 | 19 | 50660-50678 | CGTCGGCACCCAGAACACC |
|  |  | 22517-22535 | CGTCGTCACCCAGAACACC |
| GTE8-D40 | 19 | 50926-50944 | CTGGCGCAGCTCGGCGATC |
|  |  | 37551-37569 | CTTGCGCAGCTCGGCGATC |
| GTE8-D41 | 18 | 12495-12512 | TACGAGGCCGACACCGGC |
|  |  | 3435-3452 | TACGAGGCCGACACCGGC |
| GTE8-D42 | 18 | 67239-67256 | CGACCGAGACCATCCTGC |
|  |  | 21907-21924 | CGACCGAGACCATCCTGC |
| GTE8-D43 | 17 | 62595-62611 | AACCTGCTCACCGCGAC |
|  |  | 3906-3922 | AACCTGCTCACCGCGAC |
| GTE8-D44 | 17 | 60395-60411 | GCTGGTCATGGCCCTCC |
|  |  | 20192-20208 | GCTGGTCATGGCCCTCC |
| GTE8-D45 | 17 | 65256-65272 | GGATGCGCGACATGGTC |
|  |  | 21007-21023 | GGATGCGCGACATGGTC |
| GTE8-D46 | 17 | 36142-36158 | CGTCGTAGTCGTCGACG |
|  |  | 34837-34853 | CGTCGTAGTCGTCGACG |
| GTE8-D47 | 16 | 29756-29771 | CGTCGGCGCCTTCACC |
|  |  | 24983-24998 | CGTCGGCGCCTTCACC |
| GTE8-D48 | 16 | 47727-47742 | CGGTTGCCCGCGGTGA |
|  |  | 34998-35013 | CGGTTGCCCGCGGTGA |
