# Supplementary material for: Lysis to Kill: Evaluation of the Lytic Abilities, and Genomics of Nine Bacteriophages Infective for Gordonia spp. and Their Potential Use in Activated Sludge Foam Biocontrol
Source: PLoS One. 2015 Aug 4;10(8):e0134512. doi: 10.1371/journal.pone.0134512 (PMC4524720; doi:10.1371/journal.pone.0134512)
Supplement: S5 Table — (DOCX) [file pone.0134512.s005.docx]

**Table S5: Summary of *Gordonia* phage structural genes identified by mass spectroscopy**

| Phage | Gene containing motifs | Amino acid sequence | Coverage |
| --- | --- | --- | --- |
| GMA2 | *orf9* | VYTEWQDDAWGYYDAISEIK | 30.4 % |
|  |  | LYPALSIDADGVPISTSNY |  |
|  |  | GEQEKDIAGELSLPEEITDEVVK |  |
|  |  | HEELVAELFSGHGGQSSLLR |  |
|  |  | TVGDIELPR |  |
|  |  | ESDQFLVER |  |
|  |  | LLQGIDLPK |  |
|  |  | YSNAIQIDESLYK |  |
|  |  | TDPAASANAGYDNFAVSAATWR |  |
|  |  | GDVIAPTDR |  |
|  |  | NAQNDPVSPTESATTVNPGNPVGGR |  |
|  | *orf10* | KYGVPIGSPINAAK | 29.4 % |
|  |  | LSSNESNANSDLSW |  |
|  |  | TPSKDSVSAFIK |  |
|  |  | HYLLTGEK |  |
|  |  | QVTPDDLFEDPSWEHVK |  |
|  |  | HVVYETPHPDTK |  |
|  |  | QGSGSTFSESFQQYESAAK |  |
|  |  | KTLGDGTHPVVGAK |  |
|  |  | AIDADGNVGVITDVYQTYTK |  |
|  |  | SKLPDVLIGDSSHAWGTK |  |
|  |  | ERPMFFPSPDKHEK |  |
|  |  | YGLSLTPTQNK |  |
|  |  | ISDFTFGSSK |  |
|  |  | YNSHKPEYR |  |
|  |  | YGGFSGMSGIQMDK |  |
|  |  | VIEQINSNAGKEYYEPLVK |  |
|  |  | SSAVTAEVPGQFK |  |
|  |  | SEVGDYDAYTPHITAVTGQQAR |  |
|  | *orf16* | LDDLMSLDAGNREELR | 37.7 % |
|  |  | ADITAVYEEADADAPEVDEMIVAMR |  |
|  |  | ALNNFETMLETFER |  |
|  |  | SVATLIASIGEER |  |
|  |  | DALPTFGATR |  |
|  |  | YIAPPVLGAYNDAISLWTAANDANPTNPTK |  |
|  |  | FAERTLLNKISAASTK |  |
|  |  | VTTSWNQGAAR |  |
|  |  | DLLLAITR |  |
|  |  | HRIPRPVQLR |  |
|  |  | ALAPEWAR |  |
|  |  | ISWHMDDTFTSQSNNAALNDLPSSIK |  |
|  |  | DSDLVGTNDYMTFVETFEGIAK |  |
| GMA4 | *orf7* | IFANGGGTSGGAV | 30.0 % |
|  |  | VYDQLTK |  |
|  |  | GDRDVQQVAPGAEFPIVDFER |  |
|  |  | SAFQTVYGNQWK |  |
|  |  | DVLANWNVDMVASNQVPAGTAWVVAER |  |
|  |  | LEKPLSTETWR |  |
|  | *orf13* | KDGGFEFGADR | 24.6 % |
|  |  | DISETESLGYASPTRR |  |
|  |  | DVQKEDVTINFALQEFKR |  |
|  |  | SGPQETLFGR |  |
|  | *orf66* | SAAWMQLPQNLTSLEGTTK | 41.3 % |
|  |  | LDEFAAPTTAVSMGTQR |  |
|  |  | ITNLATGTGAADAVTK |  |
|  |  | AQLDAVAAVANAAASGIAIK |  |
|  |  | TNITLTGAQTIDGVAVVAGD |  |
|  |  | VLVAGQTSAAANGIYLAAAGAWS |  |
|  |  | MVAGSTGEIITAGNGLTK |  |
|  |  | ASGGITVDGTGIAVDSTIAR |  |
| GMA5 | *orf14* | RPGIYNVTAVWPWAANATGR | 9.90 % |
|  | *orf15* | TIWNVQHVNYPAR | 21.6 % |
|  |  | LIESKPGTFAVLGYSQGGAIASRIGQELLTGR |  |
|  |  | TAVEHSAFHLNYWGAR |  |
| GMA6 | *orf20* | DIYSGLLTGSVNPRGFGSVQFDPAVPR | 48.0 % |
|  |  | VRNLFPVAATSANLIDYFR |  |
|  |  | VLGFAENGGNGNAR |  |
|  |  | AAADGIAAPAGTATDTFGLKPK |  |
|  |  | FESAQAPVR |  |
|  |  | TIAHWEAAHR |  |
|  |  | SATLSVIANYPGTGFVLHPHDWEDIELQK |  |
|  |  | ANGDGQYMLVTNVAVGATTSVWR |  |
|  |  | QPVVETPAIAEGSWLTGAFGIGAQLYDR |  |
|  |  | IAEQHADFFVR |  |
|  |  | NAIAILAEER |  |
|  |  | LALAVKRPESFVKGTFV |  |
|  | *orf29* | NGPSTPLR | 60.9 % |
|  |  | SASGQAFFSGLAER |  |
|  |  | GPSDAAILIR |  |
|  |  | GLADYESVFGK |  |
|  |  | RPAYGYLYDTVK |  |
|  |  | TFFDEGGEQAYVTR |  |
|  |  | VVGPDATKGTIVLVDR |  |
|  |  | ATPTPANTLTFDAASAGAWSGDLK |  |
|  |  | IAVEDGSIADSVK |  |
|  |  | GEPVEVQNNLR |  |
|  |  | TPAQIAQR |  |
|  |  | RIALLSHNDGATK |  |
|  |  | TELAQTVTAVDDDSAGLFAPWIQVNDGAGGIR |  |
|  |  | SLSSDEQNYGFLSAR |  |
|  |  | LVVESEKR |  |
|  |  | LEDYVFAPIDSK |  |
|  |  | NQLLSAINAELVGIVEPMR |  |
|  |  | QAGGLYEQIDANGQQIDPGYMIETGNTVNSAQSLANNEVR |  |
|  |  | LSPTGALVSLDIVK |  |
|  | *orf43* | LPQWSSGTTDSPSR | 45.8 % |
|  |  | YYVDTDGVIYR |  |
|  |  | TEGNDPALWIDLPAGTTADAIR |  |
|  |  | SANASGVTVYGAMAQSGNLIETK |  |
|  |  | TSTSADLTQITASGDVNTLGR |  |
|  |  | LMAGSLTPQDAQLYIQNSGTTRPALLAR |  |
|  |  | TNTTLPNANTAVAVENQSGATQLLK |  |
|  |  | AINAADNVAVNSNYTATNSGTSSLIQAQLVFR |  |
|  |  | QQPGDTSAGSSLGVVAGTAGADGLAPER |  |
|  |  | FGVNDAK |  |
|  |  | FVANEPDWAPVVVR |  |
|  |  | GAVSQAADLLLAQDSDENKVAGINYR |  |
|  |  | SLATTGTGLNAFSGPITSAGEIQGTNLR |  |
|  |  | VIQGSGNNAGVLSQIK  INNNLELK |  |
|  |  |  |  |
| GRU3 | *orf4* | ATAMSVPAIKR | 33.7 % |
|  |  | GGTVLSDQPTWIDR |  |
|  |  | TNGPVSPYHR |  |
|  |  | GTGGAVIAADR |  |
|  |  | GENGGVAFTSNGVEVR |  |
|  |  | EHGTFDAHLLVDGR |  |
|  |  | VLSLPGSALDATVDK |  |
|  |  | ASLNYETQEGK |  |
|  |  | AADLIDYGLSAYMSPITAR |  |
|  | *orf6* | ALAVPGVEAWLR | 14.6 % |
|  |  | TVSGLVLPWNK |  |
|  |  | TTLGPLVIRPGGTR |  |
|  |  | SDGLWMDFAFAETPDADAAIAQVK |  |
|  |  | VTTLHAQHTK |  |
|  |  | ENNLMNAAAFIR |  |
|  |  | ALMAAGMSEADAR |  |
|  | *orf15* | TIWNVQHVNYPAR | 24.0 % |
|  |  | DTDSSIPGVALTGEGISGEPLGGFR |  |
|  |  | FGNSHTDYGNTSKR |  |
|  |  | TAVEHSAFHLNYWGAR |  |
| GTE6 | *orf14* | WQDEVWSLR | 18.1 % |
|  |  | VTEGVVGELAQQLFGNLPDVEQK |  |
|  |  | LVWSVHSSSELLGSQAGQYQITDGVTPR |  |
|  |  | LASGGGLLLVTQDVEILNK |  |
|  |  | ENliallsqfgqqpgaaveilr |  |
|  | *orf20* | ELLGESDRSEVDALLNEAIEK | 24.4 % |
|  |  | ADIASLQIQFPEDLVASGASDDGQVLRR |  |
|  |  | QVDFGAIYAGGMAGR |  |
|  |  | VNAISLAR |  |
|  |  | FKVVLPIWYKPTFR |  |
|  |  | TGVDNALAVTDAQIEGWFSDR |  |
|  |  | ALVYPEGTIVR |  |
|  |  | GRGDIINLEAVYDSVGLTTNDFLR |  |
|  |  | LFMEESLAIAWR |  |
|  | *orf23* | RWGYHLFPFIGAARL | 16.8 % |
|  |  | DGTGWGVGPYNVTLDDATTPAPAK |  |
| GTE8 | *orf18* | IPLFEEDRIR | 33.9 % |
|  |  | AVALQINLKR |  |
|  |  | LQFVGNGQNFPVDFGR |  |
|  |  | RVTDVPDGAVAPPSAIDALFGER |  |
|  |  | DINNQTIIKDLVPR |  |
|  |  | DGIIALDGEGDAAVAGSSELGSTMWGK |  |
|  |  | MWVSAHAIAMPVLVNYSSFMK |  |
|  | *orf24* | KPLAGIIGVAPEDLELDADFK | 28.9 % |
|  |  | SDGVVFSADTETSDVESWGALEPTR |  |
|  |  | SDQNWNSEDALVHGMTITAK |  |
|  | *orf31* | AFIEVTNGEGELLLPR | 30.3 % |
|  |  | LAPDLILEEDQDADITPQLPAGLSDVDR |  |
|  |  | GLAGDAILQANDFAAPEGGAEGMTDGTTLVWDSTVGK |  |
|  |  | IIAQITVPEQPFAWHPR |  |
|  |  | GPGHTISTFIDNYYPR |  |
